# Protein biomarkers of cardiac remodeling and inflammation associated with HFpEF and incident events

**DOI:** 10.1038/s41598-022-24226-1

**Published:** 2022-11-22

**Authors:** Jessica A. Regan, Lauren K. Truby, Usman A. Tahir, Daniel H. Katz, Maggie Nguyen, Lydia Coulter Kwee, Shuliang Deng, James G. Wilson, Robert J. Mentz, William E. Kraus, Adrian F. Hernandez, Robert E. Gerszten, Eric D. Peterson, Rury R. Holman, Svati H. Shah

**Affiliations:** 1 Duke Molecular Physiology Institute (DUMC), 300 N. Duke Street, Box 104775, Durham, NC 27701 USA; 2 Department of Medicine, Duke University, Durham, NC USA; 3 Division of Cardiovascular Medicine, Beth Israel Deaconess Medical Center, Boston, MA USA; 4 Duke Clinical Research Institute, Durham, NC USA; 5 Department of Medicine, University of Texas Southwestern, Dallas, TX USA; 6 Radcliffe Department of Medicine, University of Oxford, Oxford, UK

**Keywords:** Biomarkers, Cardiovascular biology

## Abstract

There is increasing evidence that HFpEF is a heterogeneous clinical entity and distinct molecular pathways may contribute to pathophysiology. Leveraging unbiased proteomics to identify novel biomarkers, this study seeks to understand the underlying molecular mechanisms of HFpEF. The discovery cohort consisted of HFpEF cases and non-HF controls from the CATHGEN study (N = 176); the validation cohort consisted of participants from the TECOS trial of patients with diabetes (N = 109). Proteins associated with HFpEF were included in a LASSO model to create a discriminative multi-protein model and assessed in the validation cohort. Survival models and meta-analysis were used to test the association of proteins with incident clinical outcomes, including HF hospitalization, mortality and HFpEF hospitalization in CATHGEN, TECOS and the Jackson Heart Study. In the derivation set, 190 proteins were associated with HFpEF in univariate analysis, of which 65 remained significant in the multivariate model. Twenty (30.8%) of these proteins validated in TECOS, including LCN2, U-PAR, IL-1ra, KIM1, CSTB and Gal-9 (OR 1.93–2.77, *p* < 0.01). LASSO regression yielded a 13-protein model which, when added to a clinical model inclusive of NT-proBNP, improved the AUC from 0.82 to 0.92 (*p* = 1.5 × 10^–4^). Five proteins were associated with incident HF hospitalization, four with HFpEF hospitalization and eleven with mortality (*p* < 0.05). We identified and validated multiple circulating biomarkers associated with HFpEF as well as HF outcomes. These biomarkers added incremental discriminative capabilities beyond clinical factors and NT-proBNP.

## Introduction

Heart failure with preserved ejection fraction (HFpEF) is a clinical syndrome of impaired diastolic function with symptoms of dyspnea, congestion and exercise intolerance with a preserved left ventricular ejection fraction (EF, > 45–50%)^1^. Patients with HFpEF comprise about 50% of HF patients and HFpEF is responsible for approximately half of HF hospitalizations^2^. As the global burden of obesity and diabetes increases, the prevalence of HFpEF is expected to continue to rise disproportionately to that of heart failure with reduced ejection fraction (HFrEF)^3^.

HFpEF continues to pose significant diagnostic and therapeutic challenges, in part because of marked heterogeneity in defining the clinical syndrome across studies, but also because non-cardiac causes of exertional dyspnea can be difficult to distinguish from HFpEF in compensated patients without invasive hemodynamic testing. Despite the common symptomatology and risk of adverse clinical outcomes in HFpEF and HFrEF, therapeutic strategies that improve morbidity and mortality in patients with HFrEF have not proven to be effective in HFpEF, suggesting that the molecular mechanisms of the two clinical entities may be distinct^4–6^. Importantly, the mechanisms underlying the development of diastolic dysfunction and progression of HFpEF also remain poorly understood.

Only BNP and NT-proBNP are routinely used in clinical practice as HF protein biomarkers for presence and severity of HF, agnostic of HF phenotype, in both the acute and ambulatory settings. Despite the identification of promising candidates, including markers of fibrosis (ST2, Gal-3), myocardial injury (cardiac troponins), inflammation (GDF-15) and kidney injury (LCN2) for both prediction of and prognosis in HF, these biomarkers have not yet been translated into clinical practice and have limited support specifically for HFpEF^7^. Previous studies have identified distinct profiles of inflammation and remodeling in HFpEF, but further investigation is needed to better understand the underlying pathophysiological mechanisms^8–10^.

Leveraging a high throughout proteomic profiling platform measuring 459 analytes, we investigated the association of protein biomarkers with HFpEF, validated these findings in an independent patient population and examined their prognostic utility in overall clinical events to identify novel molecular mechanisms that differentiate HFpEF from patients without HF.

## Methods

### Study populations

#### CATHGEN discovery cohort

The discovery cohort was comprised of individuals enrolled in the CATHGEN study^11^. HFpEF cases were defined as having a history of HF, EF ≥ 45% and diastolic dysfunction class ≥ 1 on echocardiogram; controls were those with no reported history of HF, EF ≥ 45% and no diastolic dysfunction^12,13^. HF hospitalizations were defined using HF-associated *International Classification of Diseases, Ninth Revision* (ICD-9) codes for emergency room visits or hospitalizations > 30 days from study enrollment (Supplemental Table S1). All-cause mortality was determined using the Social Security Death Index (SSDI) and National Death Index (NDI).

#### TECOS validation cohort

The validation cohort consisted of participants from the placebo arm of the Trial Evaluating Cardiovascular Outcomes with Sitagliptin (TECOS)^14^. Briefly, TECOS randomized participants with type 2 diabetes mellitus (DM) and established cardiovascular disease to the dipeptidyl peptidase 4 inhibitor sitagliptin or placebo. From a previously defined nested major adverse cardiovascular event (MACE) case–control subset of TECOS placebo-arm participants, HFpEF cases (defined as history of HF at enrollment and echocardiographic assessment available with EF ≥ 55%) and non-HF controls (defined as no history of HF, EF ≥ 55%) were identified; Twenty participants with HFrEF (EF < 40%) were also compared to non-HF controls; N = 440 participants had proteomic data available and were analyzed for incident events HF hospitalization and mortality.

#### Jackson heart study cohort

The Jackson Heart Study (JHS) is a prospective population-based cohort of Black adults residing in Jackson, MS, designed to investigate cardiovascular disease risk factors. Details of JHS have been published previously^15^. For the present analyses, patients with prevalent HF were excluded from analyses of time to first HFpEF hospitalization. In JHS, mortality and HF adjudication have been described previously^16^.

All methods were carried out in accordance with relevant guidelines and regulations. All study participants in CATHGEN, TECOS and JHS gave written informed consent for participation in the parent study and for use of their stored biospecimens for future use. The institutional review board at Duke University and Beth Israel Deaconess Medical Center approved the studies. A comparison of the three study cohorts included is shown in Supplemental Table S2 and additional details can be found in the Supplemental Methods.

### Proteomic profiling

Proteomic profiling was conducted in stored frozen plasma using the Olink platform, which combines an immunoassay with an oligonucleotide for greater specificity and multiplexing using the proximity extension assay (Olink Bioscience, Uppsala, Sweden)^17^. Five Olink panels were used (Cardiovascular II [CVII], Cardiovascular III [CVIII], Cardiometabolic, Metabolism and Development) which measured relative expression of a total of 459 unique proteins. CATHGEN and TECOS samples were run in the Shah Lab at the Duke Molecular Physiology Institute on the Olink 1200 platform. JHS samples were run in the Gerszten Lab at the Beth Israel Deaconess Medical Center on the Olink 1500 platform.

### Statistical analysis

**Figure 1:**
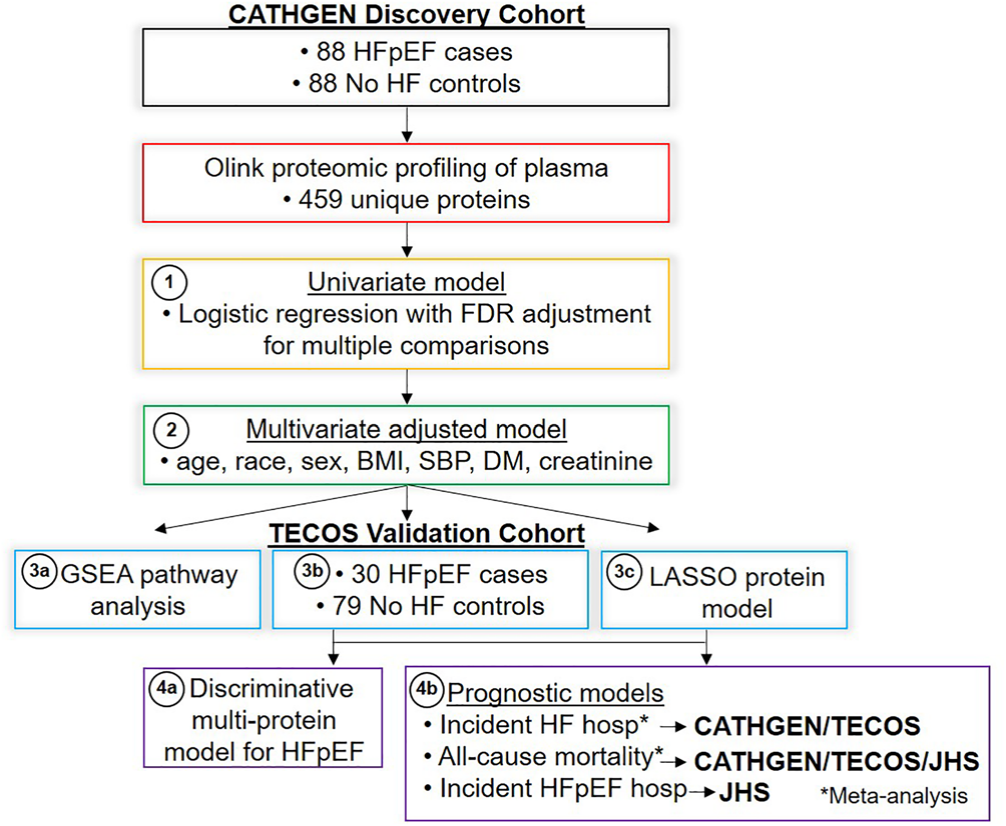
Methodologic approach to analysis of HFpEF-associated proteins. In step 1 univariate models with FDR adjustment were used to test associations between individual Olink proteins and HFpEF cases and non-HF controls in the CATHGEN discovery cohort. In step 2, significant proteins were tested in a multivariate adjusted model. In step 3, GSEA, validation in the TECOS cohort and LASSO were used to determine relevant HFpEF-associated proteins. In step 4, discriminative capabilities of multi-protein models were tested, as well as prognostic models for clinical outcomes. Abbreviations: Body Mass Index (BMI), Diabetes Mellitus (DM), False Discovery Rate (FDR), Gene Set Enrichment Analysis (GSEA), Heart Failure (HF), Heart Failure with Preserved Ejection Fraction (HFpEF), Hospitalization (hosp), Jackson Heart Study (JHS)**,** Systolic Blood Pressure (SBP), Trial Evaluating Cardiovascular Outcomes with Sitagliptin (TECOS).

The overall analysis plan is shown in Fig. 1. In step 1, the discovery aim, individual proteins were tested for association with HFpEF vs. non-HF status in the CATHGEN discovery cohort using univariate logistic regression models adjusted for multiple comparisons using a Benjamini–Hochberg false discovery rate (FDR *p* < 0.05)^18^. Significant proteins from the univariate model were then tested in step 2 using a multivariate adjusted model adjusted for age, race, sex, body mass index (BMI), systolic blood pressure (SBP), DM and creatinine (nominal *p* < 0.05). Sensitivity analyses included the addition of history of hyperlipidemia and enrollment serum hemoglobin to multivariate models.

Results from step 2 informed three parallel analyses in step 3: (a) pathway analysis using Gene Set Enrichment Analysis (GSEA); (b) individual protein validation for association with HFpEF in the TECOS cohort; and (c) creation of a sparse protein model for HFpEF discrimination in CATHGEN using an iterative LASSO-based approach, and validation of this LASSO model by logistic regression modeling of individual LASSO model proteins in TECOS (Supplemental Methods). Significant individual proteins were then tested in HFrEF compared to non-HF participants in TECOS to test specificity of protein associations for HFpEF.

Finally, in step 4, we evaluated the addition of the LASSO-derived protein model to clinical covariates from the multivariate model as well as NT-proBNP for prediction of HFpEF and association with time to clinical events in both CATHGEN and TECOS. Incremental improvement in prediction of HFpEF status was assessed using change in the AUC after addition of individual proteins identified in the LASSO model or HFpEF-associated proteins from the validation cohort. In exploratory analyses in CATHGEN, evaluation of the discriminatory performance of the LASSO model in sex-stratified analyses was also performed. Clinical events considered were any HF hospitalization (CATHGEN/TECOS), adjudicated HFpEF hospitalization (JHS), and all-cause mortality (CATHGEN/TECOS/JHS). Models for time to hospitalization were adjusted for the same clinical variables as previous models; Fine-Gray competing risk models were used in CATHGEN and TECOS, with all-cause mortality considered as a competing risk, while Cox proportional hazards models were used in JHS. Cox models were used to test for association with time to all-cause mortality and were adjusted for coronary artery disease, low density lipoprotein, history of hyperlipidemia and smoking history. The proportional hazards assumption was tested for all Cox models. Follow-up time in CATHGEN was truncated at 5 years to match the maximum follow-up time in TECOS. Results from survival models were combined using fixed effects inverse-variance weighted meta-analysis; proteins were considered significant at nominal *p* < 0.05. To understand effects independent of prevalent HF, models in CATHGEN and TECOS were also tested stratified by prevalent HF. Likelihood ratio tests (LRT) were used to compare model fit of the sparse protein model to a single protein with the strongest association with clinical outcomes. All analyses were performed using R versions 4.0.3 and 4.1.2.

## Results

### Baseline characteristics of HFpEF study populations

Baseline demographic and clinical characteristics of the CATHGEN (N = 176) and TECOS (N = 109) study populations are shown in Table 1. As expected from prior HFpEF studies^19,20^, in the CATHGEN discovery cohort HFpEF cases were older (mean age, years: 65 ± 11 vs. 53 ± 12), and had higher BMI (mean BMI, kg/m^2^: 32 ± 9 vs. 29 ± 8), SBP (mean mmHg: 151 ± 30 vs. 138 ± 25), creatinine (mean mg/dL, 1.4 ± 1.3 vs. 0.9 ± 0.2) and prevalence of DM (34% vs. 13%) compared with non-HF controls. In TECOS, similar patterns were observed (Table 1). However, unlike in CATHGEN, HFpEF cases in TECOS were not significantly older (mean age, 65 ± 8 vs. 66 ± 9) and had similar serum creatinine (mean mg/dL 1.1 ± 0.4 vs. 1.0 ± 0.3).

**Table 1:** Participant Characteristics in CATHGEN discovery cohort and TECOS validation cohort.

n	CATHGEN discovery cohort			TECOS validation cohort		
	Non-HF	HFpEF	*p*	Non-HF	HFpEF	*p*
	88	88		79	30	
Age (mean (SD))	53.1 (12.3)	64.7 (11.3)	< 0.001	65.6 (8.5)	64.5 (8.3)	NS
Female, n (%)	37 (42.0)	38 (43.2)	NS	16 (20.2)	11 (36.7)	NS
Non-white, n (%)	21 (23.9)	26 (29.5)	NS	18 (22.8)	7 (23.3)	NS
BMI, kg/m2 (mean (SD))	29.2 (7.7)	32.0 (8.5)	0.02	31.8 (19.8)	35.1 (6.8)	0.03
SBP, mmHg (mean (SD))	138.3 (25.1)	151.0 (29.7)	0.003	132.7 (19.8)	145.7 (17.4)	0.002
Creatinine, mg/dL (mean (SD))	0.93 (0.21)	1.38 (1.28)	0.001	1.04 (0.29)	1.1 (0.39)	NS
Diabetes mellitus, n (%)	11 (12.5)	30 (34.1)	0.001	79 (100)	30 (100)	NA
CAD, n (%)	48 (54.5)	46 (52.3)	NS	74 (93.7)	28 (93.3)	NS
LVEF (mean (SD))	57.9 (7.7)	57.3 (7.4)	NS	> 55 (0.0)	> 55 (0.0)	NA
Atrial fibrillation, n (%)	12 (13.6)	20 (22.7)	0.17	8 (10.01)	5 (16.7)	NS

Values represent mean and standard deviation for continuous variables; body mass index (BMI); coronary artery disease (CAD); left ventricular ejection fraction (LVEF); not significant (NS); not applicable (NA); systolic blood pressure (SBP).

### Proteins associated with HFpEF

**Figure 2:**
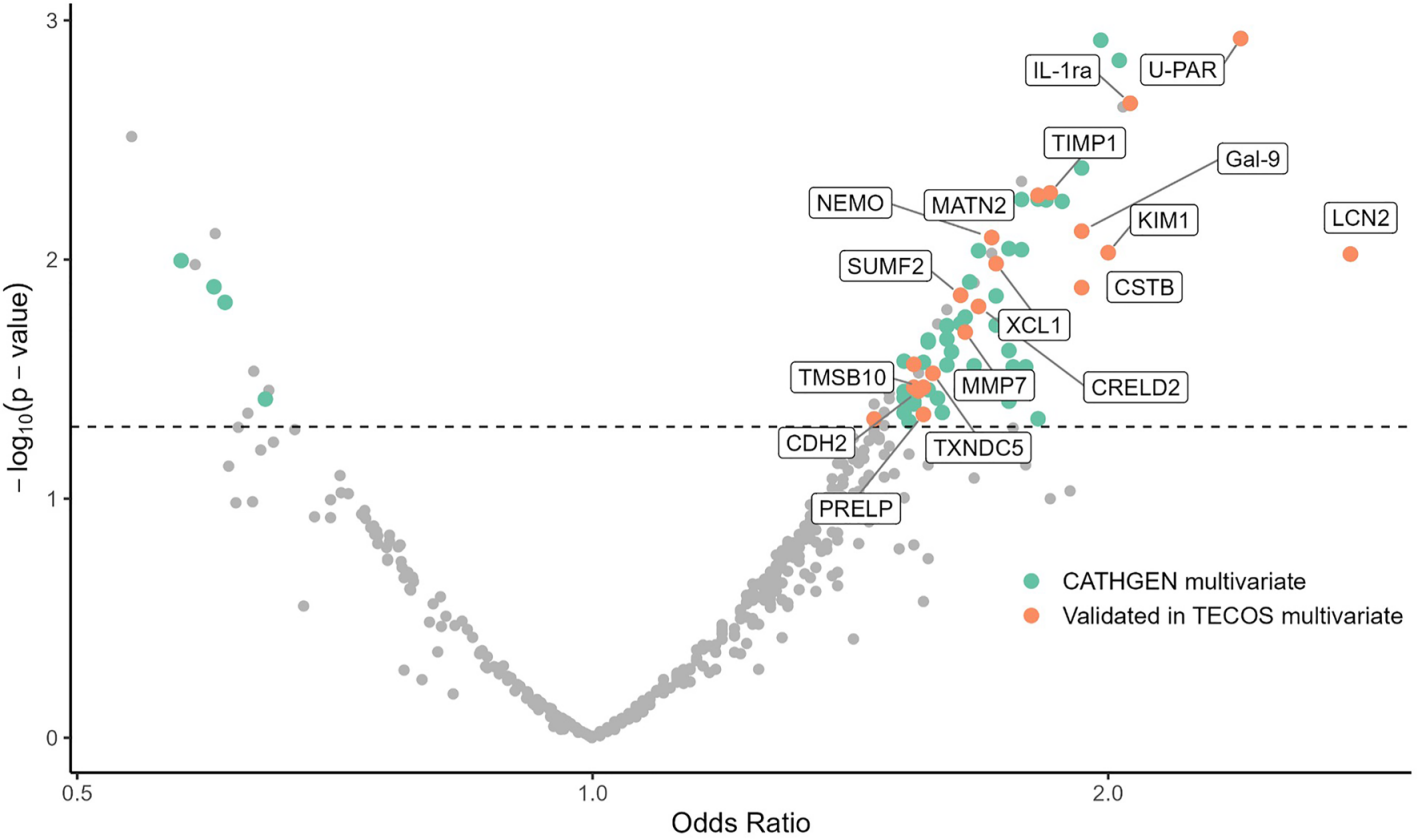
Proteins involved in cardiac remodeling, fibrosis and inflammation are associated with HFpEF. The volcano plot shows all 459 proteins analyzed in the study. Proteins with an odds ratio (OR) > 1.0 are upregulated in HFpEF cases compared to controls and proteins with an OR < 1.0 are downregulated. The 65 proteins significant in multivariate analyses in CATHGEN are shown in green. The 20 proteins that validated in the TECOS multivariate model are shown in orange.

Of 459 unique proteins assayed in CATHGEN, 446 had < 25% of values below level of detection (LOD) and thus were analyzed as continuous variables; 12 had 25–75% of values below LOD and were thus analyzed as binary traits; and one protein had > 75% of values below LOD and was removed from analysis. In univariate models, 190 proteins were associated with HFpEF (FDR *p* < 0.05, Supplemental Table S3. Of these, 65 proteins (34.2%) remained significant in the multivariate model including age, race, sex, BMI, SBP, DM, and creatinine (*p* < 0.05, Supplemental Table S3 Fig. 2). Of these 65 proteins, levels of 61 proteins were higher in HFpEF cases as compared with controls and four were lower (Fig. 2, Supplemental Figure S1). Sensitivity analyses including hyperlipidemia and serum hemoglobin identified the same 65 proteins as significant (Supplemental Table S4).

### Pathway analysis of HFpEF-associated proteins

To facilitate identification of relevant molecular pathways, GSEA was conducted using p-values from the multivariate model in CATHGEN. We restricted our tests to the 37 Hallmark gene sets and 70 KEGG pathways that included at least three proteins measured in this study. Four Hallmark gene sets and six KEGG pathways showed nominally significant enrichment in these results (*p* < 0.05, Supplemental Table S5), including enrichment of pathways of cytosolic DNA sensing and inflammation (U-PAR, TIMP1, NEMO) and fatty acid and amino acid metabolism (AOC3, CA6). No pathways were significant after adjustment for multiple comparisons.

### Validation of proteomics signatures of HFpEF

Of the 65 proteins significant in the CATHGEN discovery cohort multivariate analyses, 20 (30.8%) validated for association with HFpEF in TECOS multivariate analyses (*p* < 0.05, Table 2, Fig. 2). Of these, key proteins included those involved in cardiac remodeling, fibrosis and inflammation such as LCN2 (OR 2.58 [1.38–5.17], *p* = 4.4 × 10^–3^), U-PAR (OR 1.95 [1.06–3.85], *p* = 0.04), IL-1Ra (OR 2.17 [1.05–4.86], *p* = 0.05) and KIM1 (OR 2.46 [1.18–5.54], *p* = 0.02). Of note, four of these 20 proteins are also included in the LASSO proteomic model: KIM1, Gal-9 (OR 7.97 [1.77–42.90], *p* = 0.01), NEMO (OR 2.18 [1.29–3.93], *p* = 5.5 × 10^–3^) and SUMF2 (OR 2.89 [1.61–5.69], *p* = 9.1 × 10^–4^). In TECOS participants with HFrEF, five proteins (ANGPT2, CTSL1, NT-proBNP, THBS2 and U-PAR) were significant (Supplemental Table S6, *p* < 0.05).

**Table 2:** Proteins associated with HFpEF in both discovery CATHGEN and validation TECOS multivariate models.

Protein	CATHGEN discovery cohort				TECOS validation cohort	
	Univariate model		Multivariate model*		Multivariate model^†^	
	OR (95% CI)	*p*; FDR	OR (95% CI)	*p*	OR (95% CI)	*p*
LCN2	2.92 (1.59–5.42)	6.1 × 10^–4^; 3.6 × 10^–3^	2.77 (1.29–6.05)	0.01	2.58 (1.38–5.17)	4.4 × 10^–3^
U-PAR	3.06 (2.01–4.95)	1.1 × 10^–6^; 7.6 × 10^–5^	2.39 (1.44–4.18)	1.1 × 10^–3^	1.95 (1.06–3.85)	0.04
IL-1ra	1.92 (1.38–2.72)	1.7 × 10^–4^; 1.3 × 10^–3^	2.06 (1.32–3.35)	2.2 × 10^–3^	2.17 (1.05–4.86)	0.05
KIM1	2.86 (1.89–4.53)	2.6 × 10^–6^; 1.1 × 10^–4^	2.00 (1.21–3.49)	9.4 × 10^–3^	2.46 (1.18–5.54)	0.02
CSTB	2.86 (1.88–4.62)	4.8 × 10^–6^; 1.6 × 10^–4^	1.93 (1.17–3.35)	0.01	2.08 (1.15–4.02)	0.02
Gal-9	2.92 (2.01–4.44)	9.2 × 10^–8^; 4.2 × 10^–5^	1.93 (1.20–3.19)	7.6 × 10^–3^	7.97 (1.77–42.90)	0.01
TIMP-1	2.41 (1.67–3.63)	7.7 × 10^–6^; 2.1 × 10^–4^	1.85 (1.22–2.92)	5.3 × 10^–3^	1.93 (1.13–3.45)	0.02
MATN2	1.67 (1.22–2.33)	1.8 × 10^–3^; 7.6 × 10^–3^	1.82 (1.21–2.83)	5.4 × 10^–3^	2.51 (1.32–5.26)	8.2 × 10^–3^
XCL1	1.74 (1.26–2.47)	1.0 × 10^–3^; 5.3 × 10^–3^	1.72 (1.15–2.66)	0.01	2.80 (1.23–7.61)	0.03
NEMO	1.47 (1.08–2.02)	0.02; 0.04	1.71 (1.16–2.58)	8.1 × 10^–3^	2.18 (1.29–3.93)	5.5 × 10^–3^
CRELD2	1.82 (1.30–2.64)	8.3 × 10^–4^; 4.5 × 10^–3^	1.68 (1.12–2.62)	0.02	2.47 (1.45–4.55)	1.7 × 10^–3^
MMP7	2.03 (1.46–2.92)	5.4 × 10^–5^; 6.6 × 10^–4^	1.65 (1.09–2.54)	0.02	3.25 (1.23–11.40)	0.04
SUMF2	1.57 (1.15–2.18)	0.01; 0.02	1.64 (1.12–2.46)	0.01	2.89 (1.61–5.69)	9.1 × 10^–4^
TXNDC5	1.72 (1.25–2.44)	1.4 × 10^–3^; 0.01	1.58 (1.06–2.44)	0.03	2.56 (1.52–4.60)	7.5 × 10^–4^
PRELP	1.92 (1.34–2.92)	8.4 × 10^–4^; 4.5 × 10^–3^	1.56 (1.04–2.49)	0.04	53.52 (4.47–961.00)	3.5 × 10^–3^
TMSB10	1.70 (1.23–2.40)	1.7 × 10^–3^; 7.5 × 10^–3^	1.56 (1.04–2.38)	0.03	2.97 (1.65–6.03)	8.4 × 10^–4^
CDH2	1.75 (1.28–2.47)	8.2 × 10^–4^; 4.5 × 10^–3^	1.55 (1.04–2.37)	0.04	2.00 (1.18–3.63)	0.01
PARP-1	1.52 (1.11–2.15)	0.01; 0.03	1.54 (1.07–2.31)	0.03	2.59 (1.48–5.27)	2.6 × 10^–3^
SPINT2	1.50 (1.11–2.08)	0.01; 0.03	1.54 (1.04–2.31)	0.03	2.09 (1.22–3.87)	0.01
FCGR3B	1.44 (1.06–1.99)	0.02; 0.05	1.46 (1.01–2.16)	0.05	1.84 (1.04–3.51)	0.05

*CATHGEN multivariate models includes age, race, sex, body mass index (BMI), systolic blood pressure (SBP), type 2 diabetes mellitus (DM) and creatinine.

^†^TECOS multivariate model includes the same covariates except DM.

### LASSO proteomic models for HFpEF

Using the 65 HFpEF-associated proteins from multivariate analysis, an iterative Monte Carlo LASSO approach in CATHGEN yielded a 13-protein model (mean (SD) AUC: 0.81 (0.067), mean (SD) accuracy: 0.72 (0.068)). This model included metabolic markers (AOC3, CA5A, CA6, IGFBP3, SERPINA12, SUMF2), markers of angiogenesis (VEGFD, CLSTN2), and markers of inflammation (CCL16, Gal-9, KIM1, NEMO, PSGL-1). The AUC in TECOS of the 13-protein model was 0.80 (95% CI 0.70–0.90). This model yielded a leave-one-out cross-validation accuracy of 0.76 in CATHGEN and 0.70 in TECOS for discrimination of HFpEF.

### Incremental discriminative capabilities

**Figure 3:**
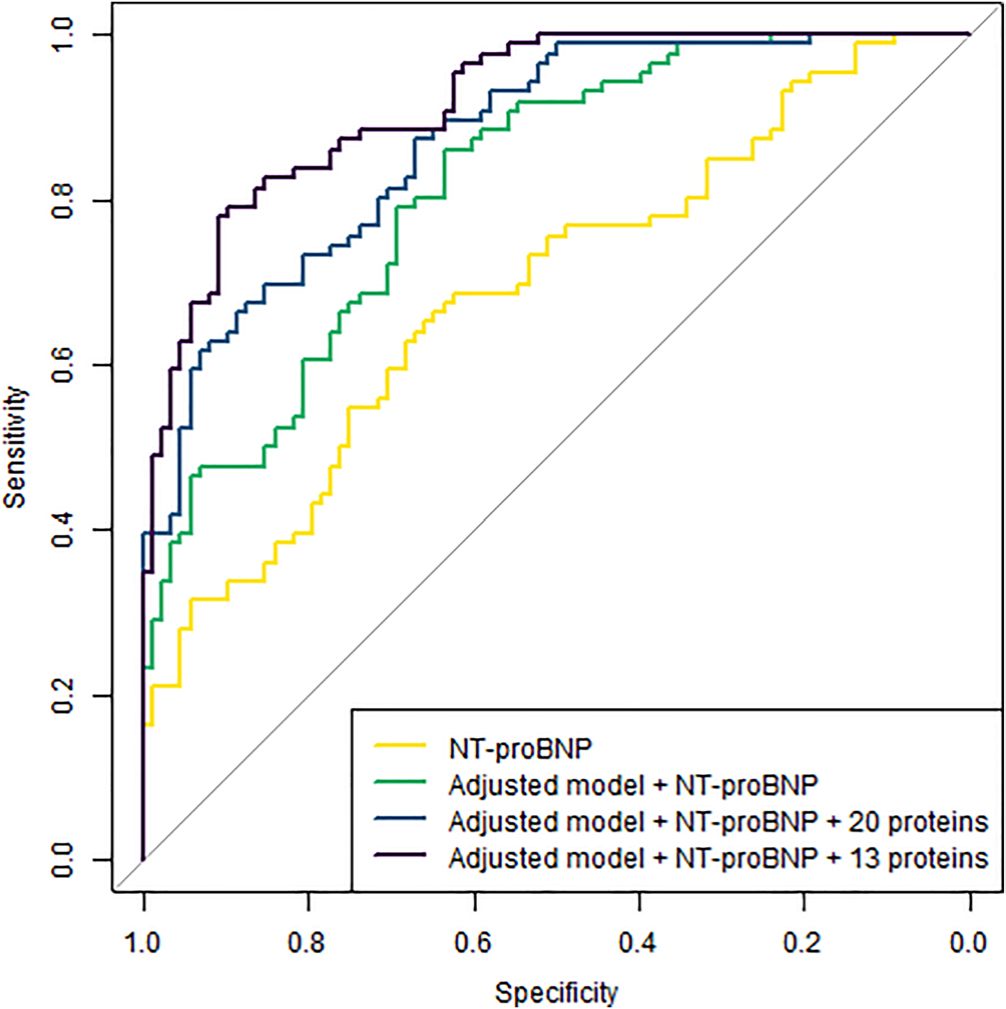
Receiver Operator Characteristic Curve for discrimination of HFpEF in CATHGEN. Shown are four Receiver Operator Characteristic (ROC) curves for discrimination of HFpEF with NT-proBNP alone, the addition of the clinical adjusted clinical model (age, race, body mass index (BMI), systolic blood pressure (SBP), type 2 diabetes mellitus (DM) and creatinine) and the addition of both the 20 validating protein model and the LASSO with Monte Carlo cross-validation 13-protein biomarker model. The 20 validating proteins are: LCN2, U-PAR, IL-1ra, KIM1, CSTB, Gal-9, TIMP-1, MATN2, XCL1, NEMO, CRELD2, MMP7, SUMF2, TXNDC5, PRELP, TMSB10, CDH2, PARP-1, SPINT2, FCGR3B. The 13 LASSO selected proteins were AOC3, CA5A, CA6, CCL16, CLSTN2 , Gal-9, IGFBP3, KIM1, NEMO, PSGL-1, SERPINA12, SUMF2, VEGFD. The addition of either the 20 or 13-protein model to the adjusted model and NT-proBNP improved the discriminative capabilities of the model.

The LASSO proteomic model, and the individual HFpEF-associated proteins that validated in TECOS, were carried forward to test discriminiative capabilities and prognostic utility. The 13 proteins identified by LASSO modeling improved the AUC when added to the adjusted model with NT-proBNP in both CATHGEN (0.92 vs. 0.82, *p* = 1.5 × 10^–4^) and TECOS (0.86 vs. 0.75, *p* = 0.01) (Fig. 3, Supplemental Table S7a). Similarly, addition of the 20 individual validating proteins to the adjusted model with NT-proBNP improved the AUC to 0.87 (*p* = 0.02) in CATHGEN and 0.94 (*p* = 4.7 × 10^–5^) in TECOS. In exploratory sex-stratified analyses, the selected set of proteins performed equally well in males and females in CATHGEN (Supplemental Table S7b).

### Prognostic capability of HFpEF proteins for incident adverse events

The median follow-up time was 1825 days in CATHGEN and 1054 days in TECOS, with 32 incident HF hospitalizations and 45 deaths, and 23 incident HF hospitalization and 54 deaths, respectively. The baseline characteristics of JHS participants are shown in Supplemental Table S8; 570 and 448 participants from JHS were included for mortality and HFpEF hospitalization analyses, respectively. In JHS, 193 individuals died and 38 individuals suffered incident HFpEF hospitalization.

**Figure 4:**
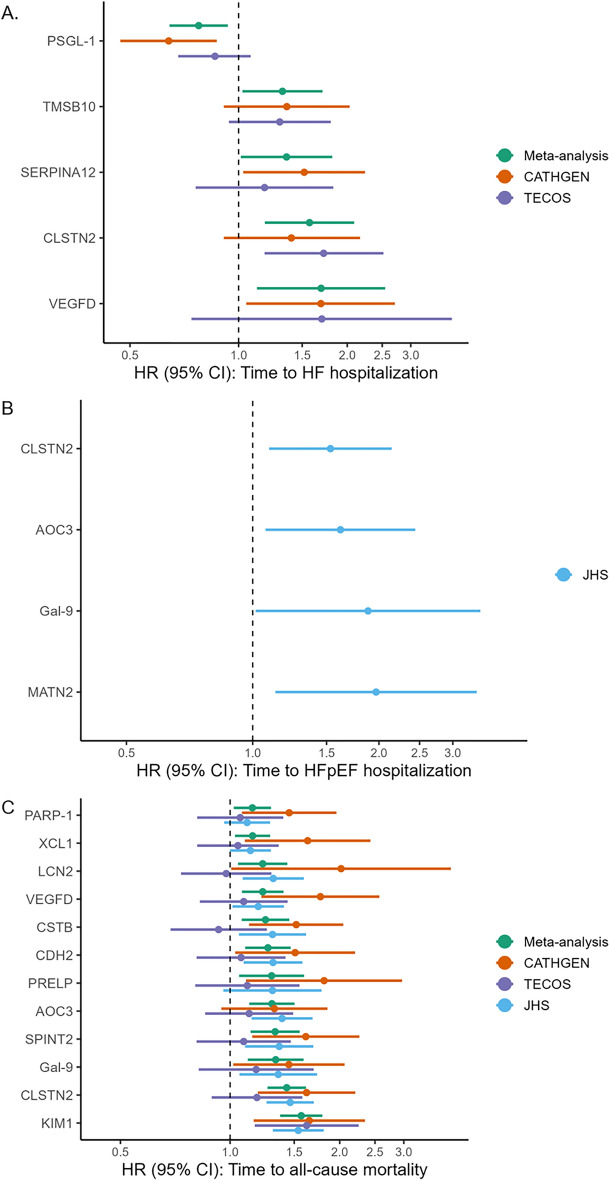
HFpEF-proteins associated with incident outcomes. The forest plots show the HR and 95% CI for the significant proteins associated with (**A**) incident HF hospitalization in CATHGEN and TECOs; (**B**) incident HFpEF hospitalization in JHS; (**C**) all-cause mortality in CATHGEN, TECOS and JHS.

**Figure 5:**
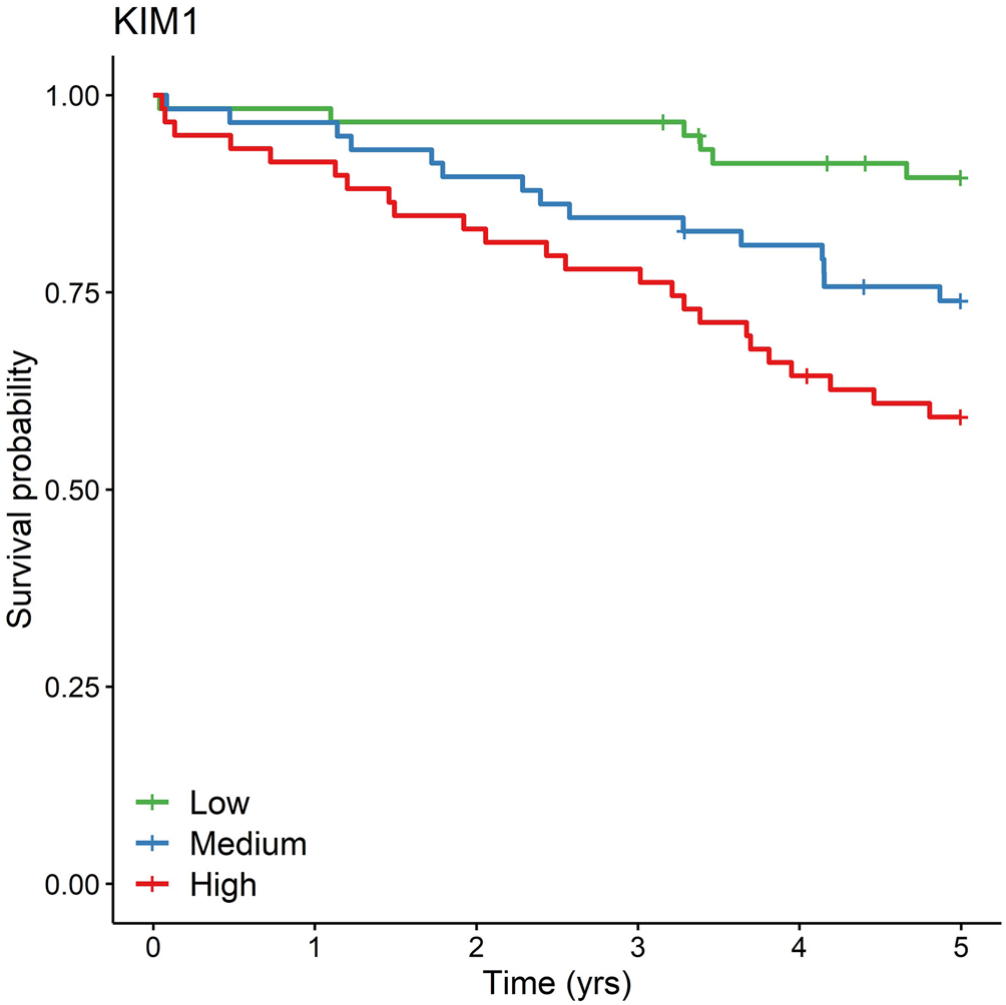
Kaplan–Meier Curve for All-Cause Mortality by KIM1 level in in CATHGEN. The Kaplan–Meier curve shows overall survival for CATHGEN participants by KIM1 levels. KIM1 was the protein most strongly associated in the meta-analysis for all-cause mortality.

Of the 29 HFpEF-associated proteins that were either selected by the LASSO model or validated in the TECOS cohort, five were significant in a meta-analysis of HF hospitalization in CATHGEN and TECOS (Fig. 4, Supplemental Table S9): PSGL-1, SERPINA12, TMSB10, CLSTN2 and VEGFD. HF hospitalization outcomes stratified by prevalent HF status are shown in Supplemental Table S10, demonstrating consistency of effects in both groups. For HF hospitalization in CATHGEN, we compared the 13-protein model to CLSTN2, the most significant protein in the meta-analyses, which gave a LRT *p*-value of 0.056, indicating a marginally improved fit after including the twelve additional proteins. For HF hospitalization in TECOS, the 13-protein model fit the data better than CLSTN2 (*p *= 0.046). HFpEF-specific hospitalization could only be evaluated in JHS, where four of the 29 HFpEF-associated proteins were significant (Supplemental Table S11): CLSTN2, AOC3, Gal-9 and MATN2 (*p* < 0.05). Eleven proteins were significantly associated with all-cause mortality (Supplemental Table S12): PARP-1, XCL1, CSTB, CDH2, PRELP, AOC3, SPINT2, Gal-9, and KIM1 (*p* < 0.05). Mortality outcomes stratified by prevalent HF status are shown in Supplemental Table S13, with most proteins showing consistency of effects in both groups, but with some proteins (Gal-9, KIM1, TIMP1, SPINT2, PARTP-1) showing stronger effects in individuals without prevalent HF. Two proteins were significantly associated with HF hospitalization as well as all-cause mortality (VEGFD and CLSTN2, *p* < 0.05). A Kaplan–Meier survival curve for KIM1 (the protein most strongly associated with mortality) in CATHGEN is shown in Fig. 5. Kaplan Meier curves for other proteins are shown in Supplemental Figures S2 and S3. Significant HFpEF-associated proteins across all analyses are shown in Supplemental Table S14. For all-cause mortality in CATHGEN, the 13-protein model fit the data better than KIM1 alone (*p* = 0.016). For all-cause mortality in TECOS, the 13-protein model did not significantly improve the model fit compared to KIM1 (*p* = 0.52).

## Discussion

**Figure 6:**
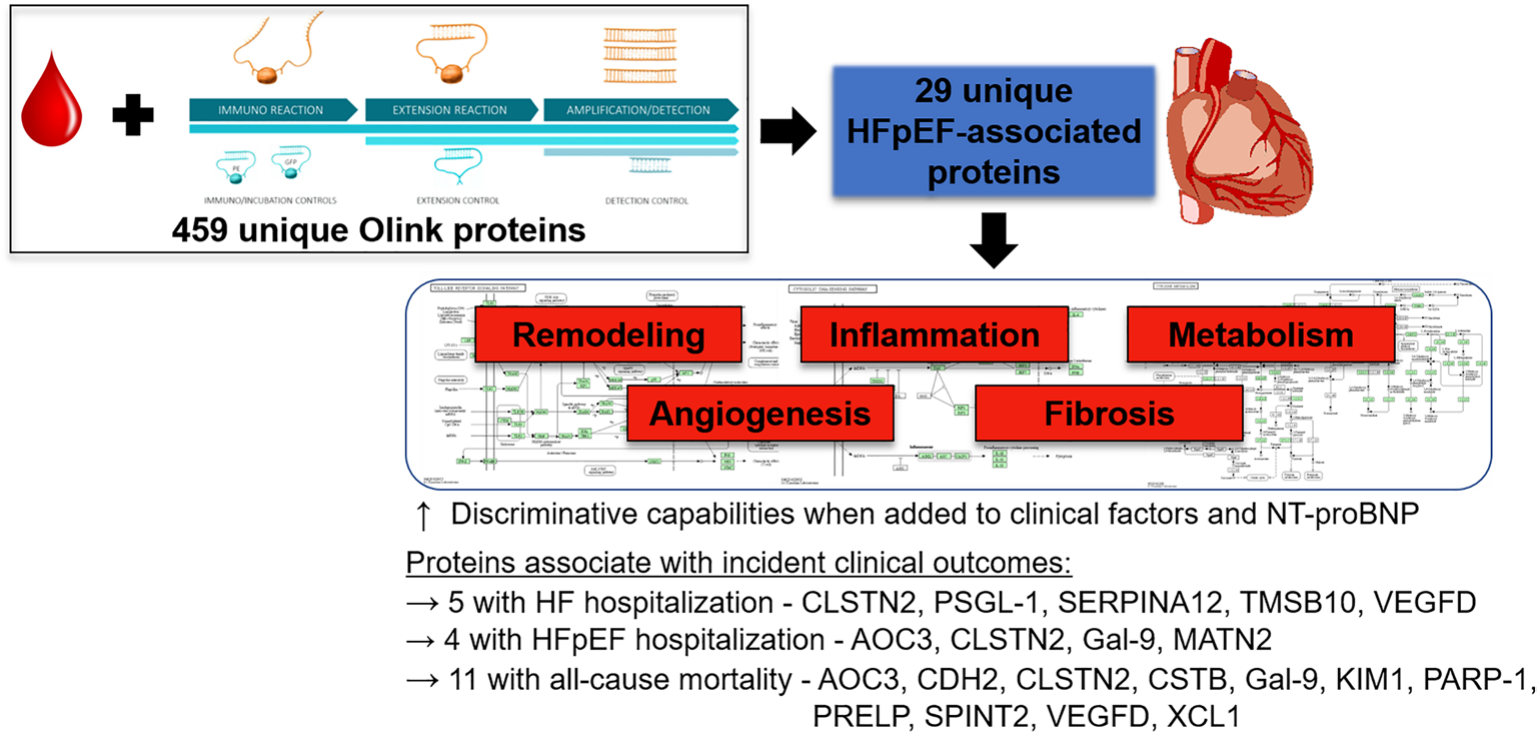
Circulating protein biomarkers associate with HFpEF, improve discriminative capabilities and predict incident events. Assessment of 459 unique Olink proteins in the CATHGEN discovery cohort followed by LASSO and validation in TECOS led to the identification of 29 HFpEF-associated proteins. These proteins were markers of cardiac remodeling, inflammation, metabolism, angiogenesis and fibrosis^22^. GSEA further identified pathways of inflammation and fatty-acid metabolism to be dysregulated. These proteins improved discriminative capabilities for HFpEF prediction and were associated with incident clinical outcomes.

Using a high-throughput platform for discovery proteomics, we have identified novel biomarkers related to fibrosis, angiogenesis, inflammation, kidney injury and fatty acid metabolism that are independently associated with HFpEF (Fig. 6). Importantly, these proteins improve discriminative capability for HFpEF when added to clinical variables and NT-proBNP, suggesting their potential value as components of multi-protein biomarker clinical tools, and a subset of these same proteins predict incident HF hospitalizations and all-cause mortality.

Specifically, we found higher levels of LCN2, a siderophore-associated protein for iron trafficking with roles in inflammation and kidney injury, to have the strongest association with prevalent HFpEF^23^. Other key inflammatory HFpEF-associated proteins were U-PAR, IL-1ra, KIM1 and Gal-9; markers of angiogenesis and remodeling (CLSTN2, VEGFD) and metabolism (AOC3, SERPINA12) as predictors of clinical outcomes; and KIM1, a marker of kidney and tubular injury with roles in immune function, was the strongest predictor of all-cause mortality even after adjustment for renal function. These protein biomarkers have potential for guiding more precise diagnostic and prognostic cardiovascular care for patients and highlight potential novel therapeutic targets by identifying relevant pathways dysregulated in human HFpEF.

The present data highlight the importance of biomarkers of fibrosis, angiogenesis and extracellular matrix remodeling in HFpEF. The mechanisms by which comorbidities may drive cardiac remodeling through endothelial dysfunction and inflammation have been proposed previously^24^. Differences in levels of remodeling proteins have recently been suggested to aid along with clinical phenogroups in early HFpEF^10^. A recent publication from the PROMIS-HFpEF study measured 248 proteins and identified inflammatory proteins to mediate comorbidity burden and cardiac structure and function in patients with HFpEF^25^. Key overlapping proteins between the PROMISE-HFpEF study and the present work include U-PAR and NEMO. Additionally, the KaRen study found that out of 98 biomarkers tested, inflammatory biomarkers were strongly associated with HFpEF severity and outcomes^26^. Here we extend the support of heightened inflammation as a pathophysiologic mechanism in HFpEF via the identification of inflammatory protein biomarkers, such as U-PAR and Gal-9. There is strong pathophysiologic overlap and feedback mechanisms linking inflammation to extracellular matrix remodeling and cardiac fibrosis. For example, in the TNFα pathways, NEMO was associated with HFpEF and was included in the sparse protein model, which may reflect not only pro-inflammatory signaling, but also myocardial remodeling^27,28^. Though the inflammatory biomarkers ST2 and Gal-3 have shown associations with HF including with pro-inflammatory comorbidities and HFpEF severity and outcomes^26,29^, these biomarkers were not associated with HFpEF in our study. Notably, we find distinct protein associations in HFrEF samples in the present analyses with U-PAR as the only protein with shared significance across HFpEF and HFrEF analyses.

Several specific remodeling and inflammatory proteins from our study warrant further investigation including LCN2, a neutrophil inflammatory lipoprotein and marker of tubular injury which is associated with cardiac hypertrophy and diastolic dysfunction, and predicts cardiovascular events in patients with chronic kidney disease^23^. LCN2 is also important for iron trafficking, which has implications for innate immune processes. Growing evidence of the value of iron repletion in HF patients may further support its importance in HF-relevant pathophysiologic processes^30,31^. KIM1, a transmembrane glycoprotein and marker of renal tubular damage that also plays a role in innate immunity, has previously been associated with adverse HF outcomes. The upregulation of both LCN2 and KIM1 in our study adds to the growing body of evidence around the importance of inflammation, comorbid chronic kidney disease and impaired renal sodium handling in HFpEF pathophysiology^32^. Gal-9, an inflammatory protein produced by the extracellular matrix that signals through regulatory T cells, is elevated in DM and chronic kidney disease^33^. Recent studies identified Gal-9 to be associated with incident HF hospitalizations^34,35^. The present findings highlight the complex interplay of multiple comorbidities and bring focus to Gal-9 as a shared biomarker reflecting inflammatory and remodeling mechanisms across disease states that may be key to HFpEF pathophysiology.

Assessment of the prognostic value of these HFpEF-associated proteins for clinical outcomes revealed markers of angiogenesis to be significant. CLSTN2 was associated with incident HF hospitalization, HFpEF hospitalization and mortality across our study cohorts. CLSTN2 is predominantly expressed in neurons and is involved in synaptic transmission, but is also expressed in adipose and cardiac tissue^36^. Through its role as a cadherin and adhesion molecule, CLSTN2 may contribute to extracellular matrix remodeling following pro-angiogenic stimuli and therefore warrants further evaluation in preclinical and clinical HFpEF research^37^. VEGFD, a secreted regulator of angiogenesis, was associated with HFpEF and incident HF in the present analyses. VEGFD stimulates cardiac fibrosis and has a growing body of support in HF and pulmonary hypertension^38^. Incorporation of extracellular matrix degradation pathway and matrix biomarker profiling into HF and HFpEF prognostication has been proposed previously and the results of the present study support these efforts^39^.

Several proteins associated with dysregulated fatty acid metabolism were significant in our study. Comorbid conditions of obesity, metabolic syndrome and diabetes are well described in HFpEF. Metabolomic analyses from the CATHGEN cohort have previously identified elevated levels of long-chain acylcarnitines, markers of impaired mitochondrial fatty acid oxidation, in HFpEF and HFrEF compared to controls^40^. In the present study, metabolic proteins including AOC3, IGFBP3 and SERPINA12 were selected in the sparse protein model for discriminative capability of predicting HFpEF and AOC3 and SERPINA12 were associated with risk of incident clinical outcomes. Adding support to the importance of dysregulated metabolism in HFpEF, AOC3, also known as vascular adhesion protein, is involved in leukocyte trafficking in response to inflammation and adipogenesis^41^. AOC3 has marked homology with copper-dependent semicarbazide-sensitive amine oxidases (SSAO), whose products cause endothelial damage and oxidative stress^42,43^. Circulating activity of AOC3/SSAO increase during biological stress in multiple pathologic states including HF, obesity, atherosclerosis, diabetes and inflammatory liver diseases, and are an independent marker of mortality in patients with chronic HF^44–48^. Here for the first time, we show elevated levels of AOC3 to be associated with prevalent HFpEF and to have discriminative capabilities in HFpEF prediction and risk of incident HFpEF hospitalization. Lower levels of IGFBP3 were associated with HFpEF in CATHGEN^49^, as well as incident HF hospitalization across CATHGEN and TECOS. In endothelial cells, IGFBP3 has antiproliferative and antiapoptotic effects via inhibition of VEGF^50^. The pleiotropic effects of IGFBP3 on cell growth and metabolism via both IGF-independent and IGF-mediated pathways warrant future investigation in HFpEF. The present work shows that higher levels of pro-angiogenic proteins including VEGF-D and CLSTN2 and lower levels of anti-angiogenic IGFBP3, are associated with HFpEF and incident outcomes. Taken together, these results suggest the opportunity for earlier therapeutic interventions targeting metabolic and angiogenic pathways to prevent HFpEF morbidity and mortality.

There are several strengths to our discovery proteomics approach to diagnostic and prognostic markers of HFpEF. We coupled high-throughput proteomics with a discovery and validation cohort and extended prognostic findings in a JHS, a third independent cohort; in fact, our study represents the largest number of proteins analyzed in HFpEF thus far. Further, we utilized traditional single-marker analyses adjusted for multiple comparisons, but also used pathway analyses given the large number of proteomic pathways represented. Limitations to the study include that Olink protein panels include prespecified proteins of interested compared to other proteomic platforms which allow for the simultaneous measurement of a larger number of proteins, and Olink is relative quantification. However, as evidence for robustness of our assays in orthogonal assessments, we note that five of the 13 proteins in the HFpEF proteomic-model are on the SomaScan platform and are highly correlated with Olink values^51^; and 11 of the proteins in the 13-protein model have known *cis* protein quantitative trait loci (pQTL)^51^ supporting the binding specificity of the assay for its cognate protein. Future work should validate levels of the protein findings here across alternative technologies and consider serial measurements in prospective studies. Diastolic dysfunction was assessed clinically in CATHGEN using the 2009 American Society of Echocardiographic guidelines^21^ given the time period of the cohorts, and further diastolic dysfunction was not available in TECOS. The appropriate cutoff for EF to study HFpEF is not well established and the data in the present study does not allow for ascertainment of whether some patients may have recovered EF from previously < 40%. In TECOS, only a subset of patients had an EF assessment available limiting our sample size in the validation cohort and EF was only reported as a binned value with cutoffs including 40–55% or > 55%. As all participants in the TECOS validation cohort have DM this may limit the ability to validate proteins that may play a role in HFpEF independent of DM and the generalizability of these data to patients with HFpEF without DM. Given the broad discovery proteomics approach, but limited number of HF events, we applied stringent adjustment for multiple comparisons and only tested proteins that either validated in TECOS or were selected by the LASSO model to limit type-1 error. Given the large number of proteins tested in our models there is mild overfitting in the LASSO models but similar to the clinical model (13-protein model calibration slope 0.73; clinical model slope 0.83), but we note that the primary goal of this discovery study was to identify potential HFpEF protein biomarkers as opposed to fitting sparse discriminative models. In CATHGEN, HF hospitalization was only able to be ascertained based on ICD diagnosis codes linked to hospitalizations, whereas events were adjudicated in TECOS and JHS, including HFpEF-specific hospitalization in JHS. These different methods for determination of HF outcomes may bias the results given limitations in specificity of the ascertainment in CATHGEN and TECOS. Only two proteins were significant across all three cohorts for HFpEF in CATHGEN and TECOS and HFpEF hospitalization in JHS. This suggests some proteins which discriminate HFpEF may not be prognostic and may reflect sample size limitations and cohort heterogeneity. However, overall the results of this study suggest the diagnostic and prognostic utility of these biomarkers and point to potential dysregulated biologic pathways in HFpEF pathophysiology. Finally, NT-proBNP was not significant in our validation cohort, likely due to the small sample size, however the direction of effect was as expected.

In conclusion, in a large discovery proteomics study of 459 proteins, we have identified circulating biomarkers of fibrosis, inflammation, kidney injury and fatty acid metabolism to be altered in HFpEF and to improve discriminative prediction when added to clinical variables and NT-proBNP. Our findings strengthen the importance of cardiac remodeling and systemic inflammation in HFpEF compared to patients without HF. We have also shown the prognostic value of these proteins for incident HFpEF hospitalization, HF hospitalization and all-cause mortality. These results suggest the importance of the relevant pathways to HFpEF pathophysiology that also contribute to adverse outcomes and may offer novel diagnostic tools and highlight future therapeutic targets.

## Supplementary Information

Supplementary Information 1.

Supplementary Information 2.

## Data Availability

Summary level and deidentified datasets generated analyzed during the current study are available from the corresponding author on reasonable request and with approval through established parent clinical trials and cohort study committees.

## Abbreviations

HFpEF: Heart failure with persevered ejection fraction
CATHGEN: Catheterization genetics
TECOS: Trial Evaluating Cardiovascular Outcomes with Sitagliptin
JHS: Jackson Heart Study
NT-proBNP: N-terminal-pro hormone B-type Natriuretic Peptide
