# Supplementary material for: Protein biomarkers of cardiac remodeling and inflammation associated with HFpEF and incident events
Source: Sci Rep. 2022 Nov 22;12:20072. doi: 10.1038/s41598-022-24226-1 (PMC9684116; doi:10.1038/s41598-022-24226-1)
Supplement: Supplementary file 2 — Supplementary Information 2. [file 41598_2022_24226_MOESM2_ESM.pdf]

Supplemental Methods

Study Populations

<u>CATHGEN Discovery Cohort.</u>

The discovery cohort was comprised of individuals enrolled in the CATHGEN study of 9334 patients who underwent cardiac catheterization at Duke University Medical Center (Durham, NC) between January 2001 and December 2010.[1] Femoral arterial blood samples were collected from participants who had been fasting for at least 6 hours prior to sample collection, prior to supplemental heparin if used. Samples were processed immediately to separate plasma and frozen at -80°C. Demographics and comorbidities were collected through medical record review at study enrollment, and yearly follow-up was conducted for events and vital status. These data were supplemented with electronic health records, including imaging data.

In CATHGEN, HFpEF cases were defined as having a history of HF, EF ≥45% and diastolic dysfunction class ≥1 on echocardiogram; controls with no reported history of HF, EF ≥45% and no diastolic dysfunction were also selected.[2, 3] History of HF was determined by the enrolling clinician at the time of study enrollment. Assessment of EF by ventriculogram or echocardiogram occurred within 365 days of cardiac catheterization and study inclusion. Patients were excluded from analysis if they had a history of cardiac transplantation or end-stage renal disease.

<u>TECOS Validation Cohort.</u>

The validation cohort consisted of participants from the placebo arm of the Trial Evaluating Cardiovascular Outcomes with Sitagliptin (TECOS).[4] Briefly, TECOS was a randomized controlled trial of sitagliptin, a dipeptidyl peptidase 4 inhibitor, vs. placebo in individuals with type 2 diabetes and established cardiovascular disease. Randomization occurred between December 2008 and July 2012 and the study concluded in March 2015. Participants were at least 50 years of age with a glycated hemoglobin level (HbA_{1c}) between 6.5 and 8.0%, and were followed for a median of 3.0 years.

For the present analyses, HFpEF cases and non-HF controls were selected from a previously defined nested major adverse cardiovascular event (MACE) case-control subset of TECOS placebo-arm participants. HFpEF cases were defined as having a history of HF at enrollment and an EF ≥55% and non-HF controls had no history of HF and an EF ≥55%. Availability of EF in TECOS trial participants was a limiting factor for HFpEF case-control definition. Twenty participants with HFrEF (EF<40%) were also compared to non-HF controls. 440 placebo-arm TECOS participants had proteomic data available and were included for incident event analyses in the present study. Incident events assessed included HF hospitalization and mortality as adjudicated by the parent TECOS trial.

<u>Jackson Heart Study Cohort.</u>

The Jackson Heart Study (JHS) is a prospective population-based cohort of Black adults residing in Jackson, MS, designed to investigate cardiovascular disease risk factors.[5] Briefly, participants were recruited between 2000-2004 with 2 follow-up examinations (2005-2008 and 2009-2012) and additional surveillance occurred via annual telephone follow-up and medical records review for adjudication of specified events. Participants for the present study were

selected based on those with Olink proteomic profiling available. All-cause mortality outcomes were collected during follow-up between 2005 and 2018[6]. Median follow-up time for participants included in all-cause mortality analyses was 15.5 years. Patients with prevalent HF were excluded from analyses of time to first HF hospitalization. HF adjudication began January 1, 2005 and extended through December 31, 2016 by review of medical records with ICD-9 codes for primary diagnoses of HF. Adjudication included review of clinical documentation, laboratory tests and imaging. HFpEF hospitalization adjudication was performed on a subset of participants by review of echocardiograms and other cardiac imaging with EF>50%. Patients with a HFrEF hospitalization (EF<50%) were excluded from these analyses. Median duration of follow-up was 12.0 years for participants included in HFpEF hospitalization analyses.

Proteomic Profiling

Proteomic profiling was conducted in stored plasma using the Olink platform, which combines an immunoassay with an oligonucleotide for greater specificity and multiplexing through the proximity extension assay (Olink Bioscience, Uppsala, Sweden).[7] Five Olink panels were used (Cardiovascular II [CVII], Cardiovascular III [CVIII], Cardiometabolic, Metabolism and Development) for a total of 459 unique proteins. Laboratory staff performing proteomic assays were blinded to clinical sample classification and clinical outcomes of participants. CATHGEN and TECOS samples were run in the Shah Lab at the Duke Molecular Physiology Institute on the Olink 1200 platform. JHS samples were run in the Gerszten Lab at the Beth Israel Deaconess Medical Center on the Olink 1500 platform. Spearman correlation of significant proteins across Olink 1200 and 1500 platforms for 369 JHS participants with measurements on both platforms is shown in **Supplemental Table 15**. All samples passed quality control. The Olink platform produces a $\log_2$-based relative measure of protein expression (NPX); NPX values are calculated for all samples, even if the NPX value is below a given assay's limit of detection (LOD). In CATHGEN and TECOS, proteins for which ≥75% of samples had NPX values below the LOD were excluded in analysis, in accordance with recommendations from Olink, and proteins with 25-75% of samples below the LOD were treated as binary (detected vs. undetected). NT-proBNP was measured on two different panels (CVIII and Metabolism) with >99% correlation; we removed the CVIII assay from analysis because it had slightly more values below LOD. K-nearest neighbor (kNN) imputation was used for TECOS data from five panels that were missing at random.[8]

Statistical Analysis

Step 3a: For GSEA, genes were ranked based on the protein's p-value from the CATHGEN multivariate model.[9, 10] We tested all MSigDB hallmark gene sets[11] and Kyoto Encyclopedia of Genes and Genomes (KEGG) pathways[12] with at least three proteins measured in CATHGEN.

Step 3b: For the validation in TECOS, we used a parallel multivariate model for logistic regression, excluding DM as all participants have diabetes. Proteins were considered significant at $p<0.05$.

Step 3c: For the LASSO approach, we used a penalized regression approach to create a sparse model of proteins that discriminate HFpEF cases from non-HF controls, beginning with the set

of proteins that were significant in the CATHGEN multivariate model. In this iterative Monte Carlo approach, the CATHGEN dataset was split randomly into a training set (80%) and test set (20%), with equal proportions of cases in each. LASSO regression was then performed using 10-fold cross-validation in the training set, with this model used to measure prediction accuracy and model performance in the test set. This process was repeated for 1000 iterations, and proteins were retained in the final sparse model if they were included in ≥50% of LASSO models. To examine the validity of this accuracy estimate we conduced a leave-one-out cross-validation test in both CATHGEN and TECOS using the sparse protein model.

Step 4b: To mitigate proportional hazards assumption violations in model covariates, CATHGEN all-cause mortality models were stratified based on creatinine levels (<1.2 vs. ≥1.2 mg/dl.

References:

1. Kraus WE, Granger CB, Sketch MH, Jr., et al. A Guide for a Cardiovascular Genomics Biorepository: the CATHGEN Experience. *J Cardiovasc Transl Res*. Nov 2015;8(8):449-57. doi:10.1007/s12265-015-9648-y
2. Pitt B, Pfeffer MA, Assmann SF, et al. Spironolactone for heart failure with preserved ejection fraction. *N Engl J Med*. Apr 10 2014;370(15):1383-92. doi:10.1056/NEJMoa1313731
3. Solomon SD, Rizkala AR, Gong J, et al. Angiotensin Receptor Neprilysin Inhibition in Heart Failure With Preserved Ejection Fraction: Rationale and Design of the PARAGON-HF Trial. *JACC Heart Fail*. Jul 2017;5(7):471-482. doi:10.1016/j.jchf.2017.04.013
4. Green JB, Bethel MA, Armstrong PW, et al. Effect of Sitagliptin on Cardiovascular Outcomes in Type 2 Diabetes. *N Engl J Med*. Jul 16 2015;373(3):232-42. doi:10.1056/NEJMoa1501352
5. Taylor HA, Jr., Wilson JG, Jones DW, et al. Toward resolution of cardiovascular health disparities in African Americans: design and methods of the Jackson Heart Study. *Ethn Dis*. Autumn 2005;15(4 Suppl 6):S6-4-17.
6. Keku E, Rosamond W, Taylor HA, Jr., et al. Cardiovascular disease event classification in the Jackson Heart Study: methods and procedures. *Ethn Dis*. Autumn 2005;15(4 Suppl 6):S6-62-70.
7. Olink. https://www.olink.com
8. Kowarik A, Templ M. Imputation with the R Package VIM. *J Stat Softw*. Oct 2016;74(7)doi:10.18637/jss.v074.i07
9. Subramanian A, Tamayo P, Mootha VK, et al. Gene set enrichment analysis: a knowledge-based approach for interpreting genome-wide expression profiles. *Proc Natl Acad Sci U S A*. Oct 25 2005;102(43):15545-50. doi:10.1073/pnas.0506580102
10. Mootha VK, Lindgren CM, Eriksson KF, et al. PGC-1alpha-responsive genes involved in oxidative phosphorylation are coordinately downregulated in human diabetes. *Nat Genet*. Jul 2003;34(3):267-73. doi:10.1038/ng1180
11. Liberzon A, Birger C, Thorvaldsdottir H, Ghandi M, Mesirov JP, Tamayo P. The Molecular Signatures Database (MSigDB) hallmark gene set collection. *Cell Syst*. Dec 23 2015;1(6):417-425. doi:10.1016/j.cels.2015.12.004
12. Kanehisa M, Goto S. KEGG: kyoto encyclopedia of genes and genomes. *Nucleic Acids Res*. Jan 1 2000;28(1):27-30. doi:10.1093/nar/28.1.27

Supplemental Figure 1. Heat Map of Protein Levels in CATHGEN HFpEF Cases and non-HF Controls. The heatmap shows the upregulation (red) or downregulation (blue) of the 65 proteins significant in the multivariate model in the CATHGEN discovery cohort. HFpEF cases are shown on the left and No HF controls on the right.

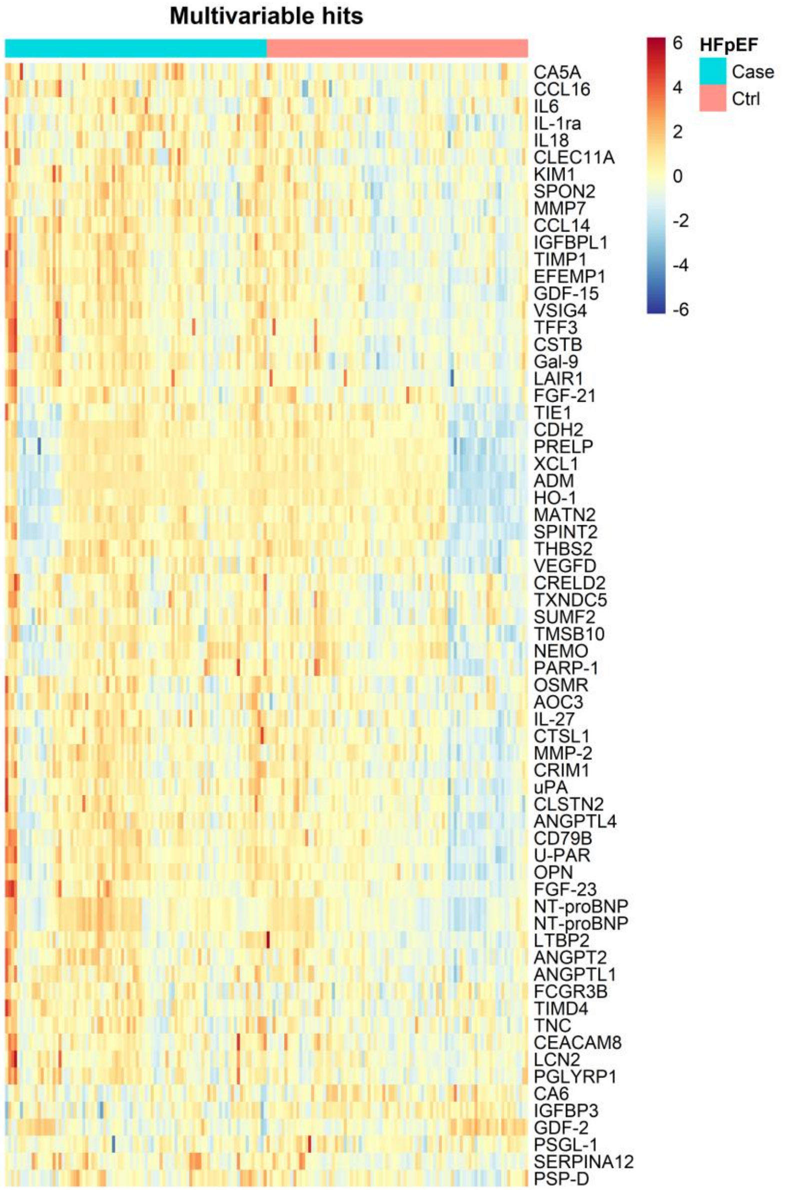

Supplemental Figure 2. Kaplan-Meier curves for HF Hospitalization by protein level in CATHGEN. Each curve shows time to HF hospitalization by protein level tertile in CATHGEN for the 29 proteins that were either selected by LASSO or validated in TECOS.

A.

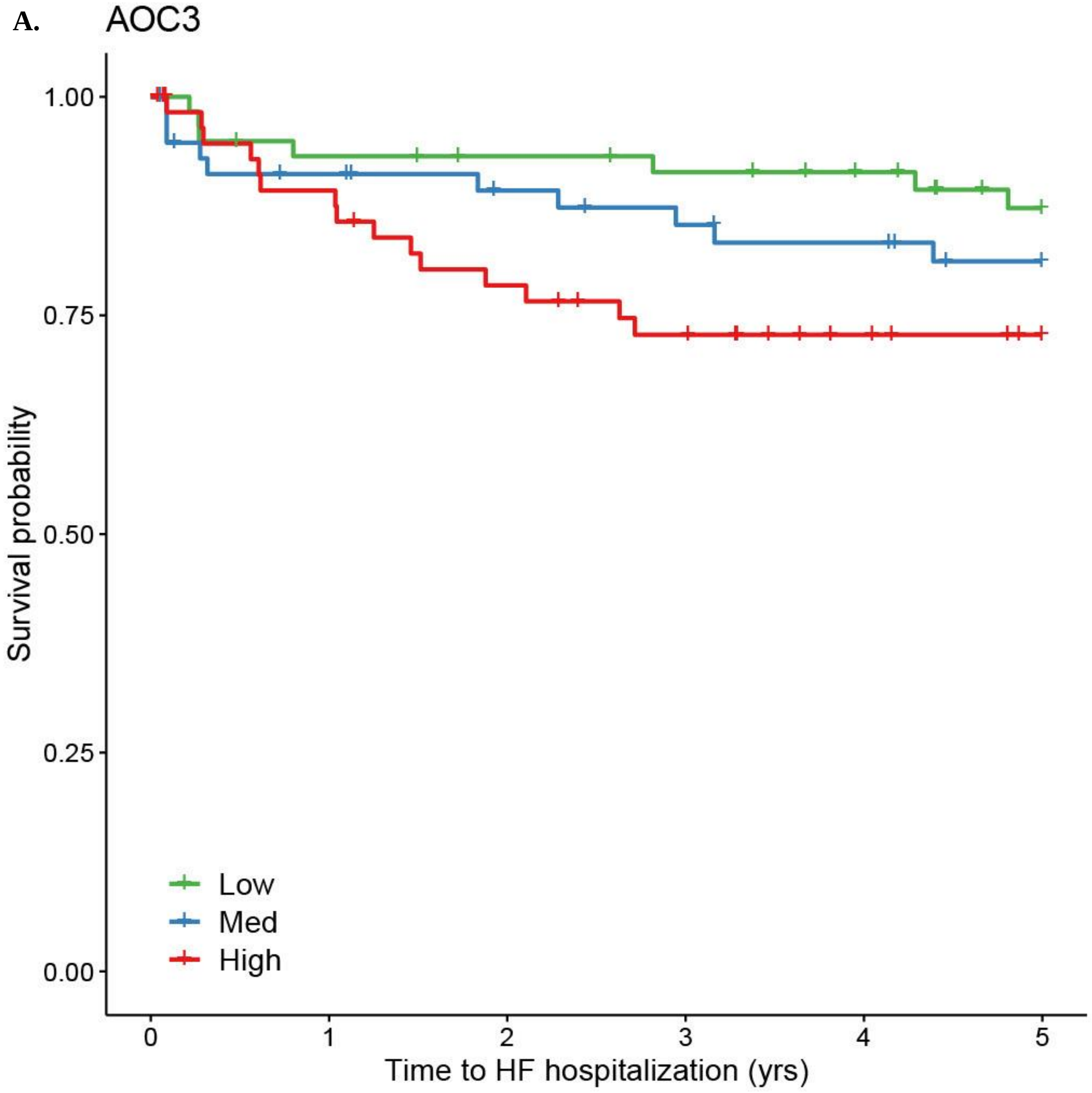

B.

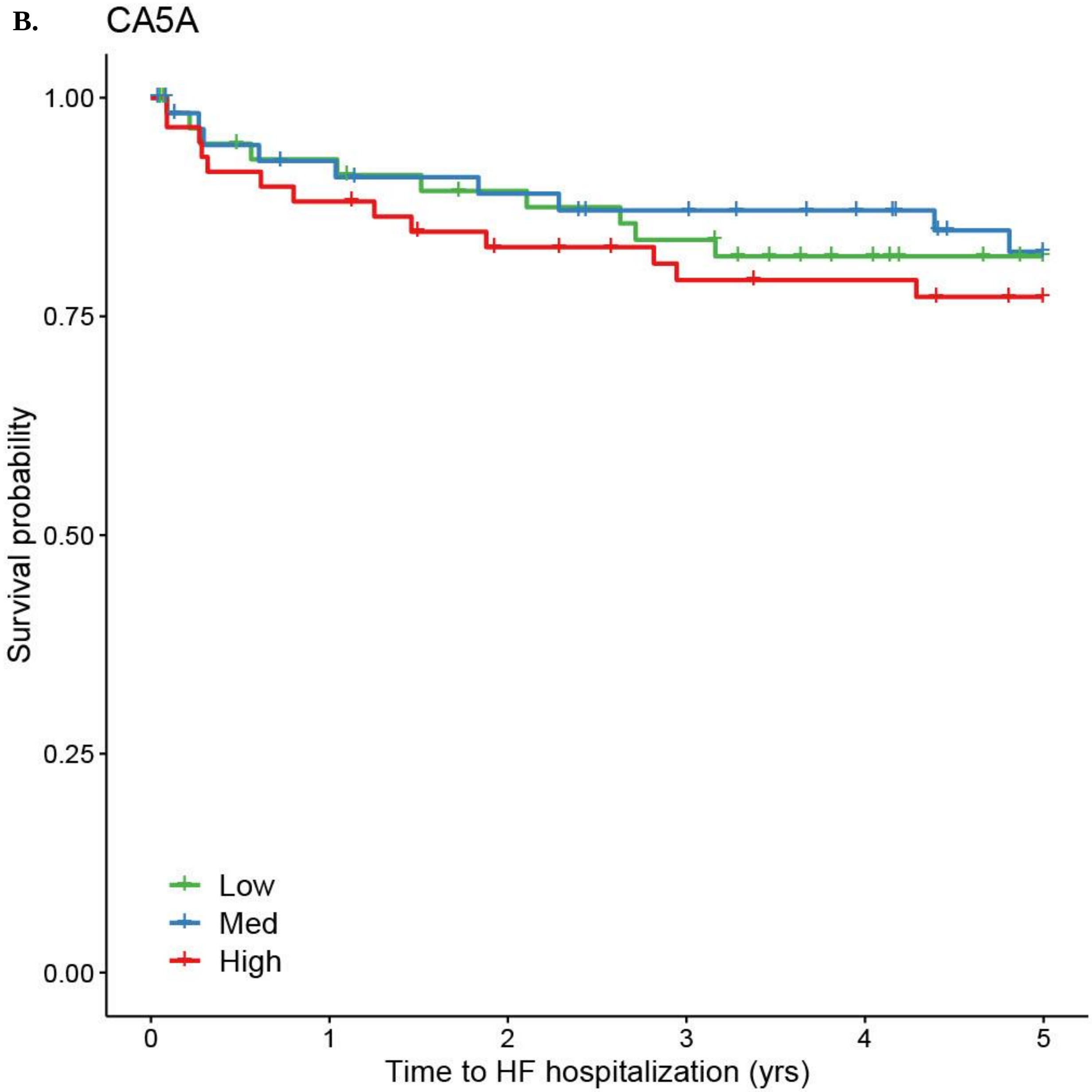
CA5A
1.00
0.75
0.50
0.25
0.00
Survival probability
0
1
2
3
4
5
Time to HF hospitalization (yrs)
Low
Med
High

C.

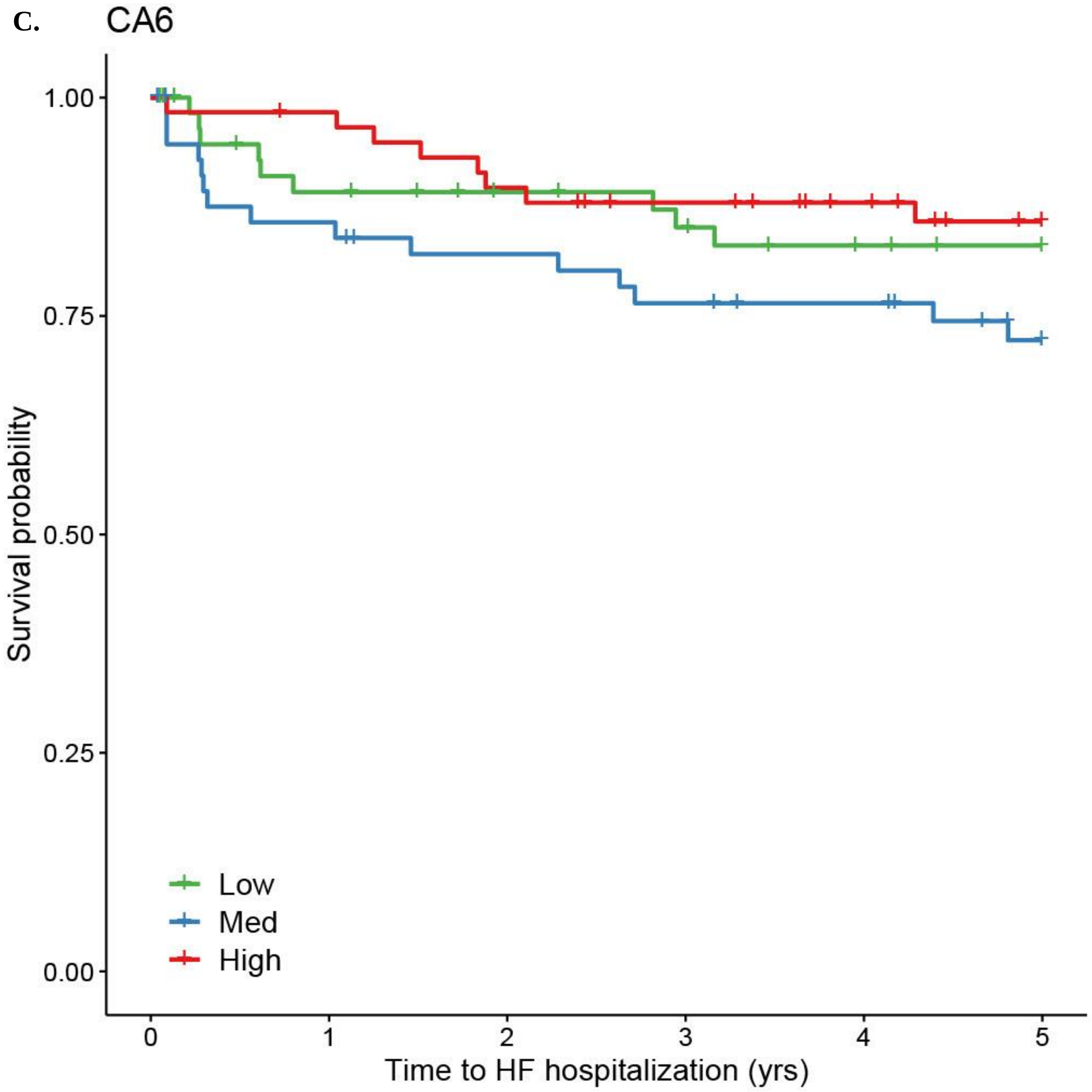

CA6
1.00
0.75
0.50
0.25
0.00
Survival probability
0
1
2
3
4
5
Time to HF hospitalization (yrs)
Low
Med
High

D.

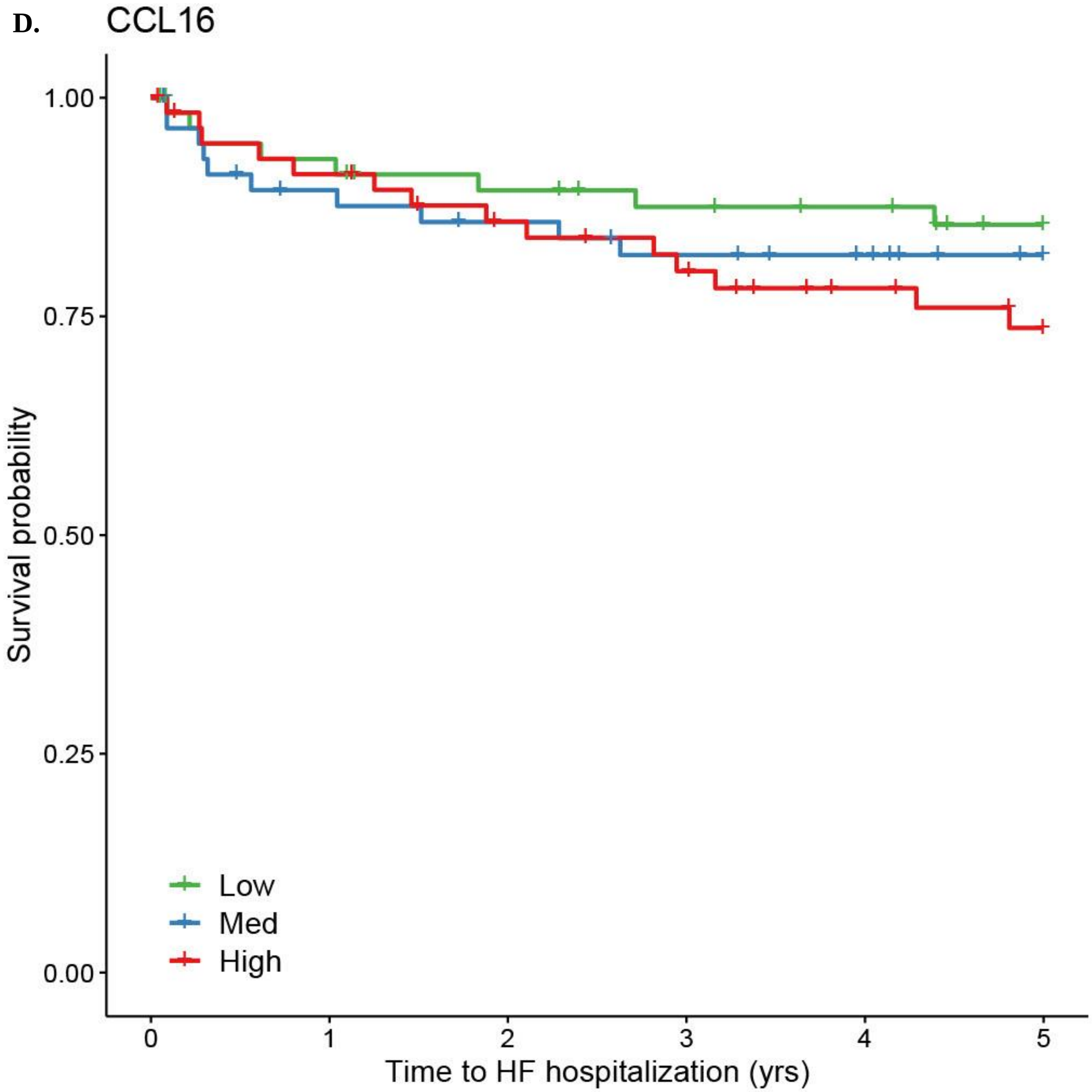
CCL16
Survival probability
1.00
0.75
0.50
0.25
0.00
0
1
2
3
4
5
Time to HF hospitalization (yrs)
Low
Med
High

E.

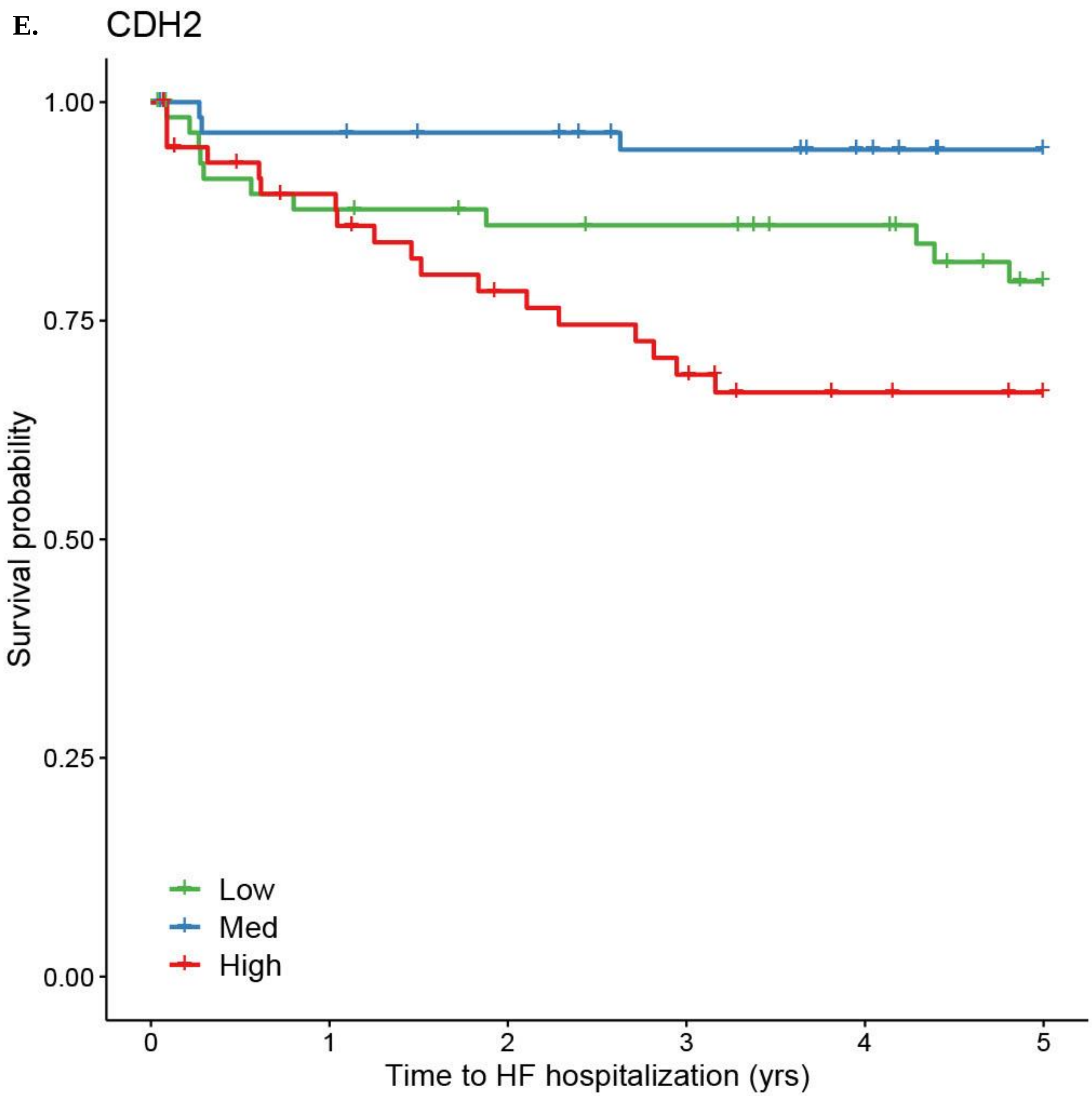
CDH2
1.00
0.75
0.50
0.25
0.00
Survival probability
0
1
2
3
4
5
Time to HF hospitalization (yrs)
Low
Med
High

F.

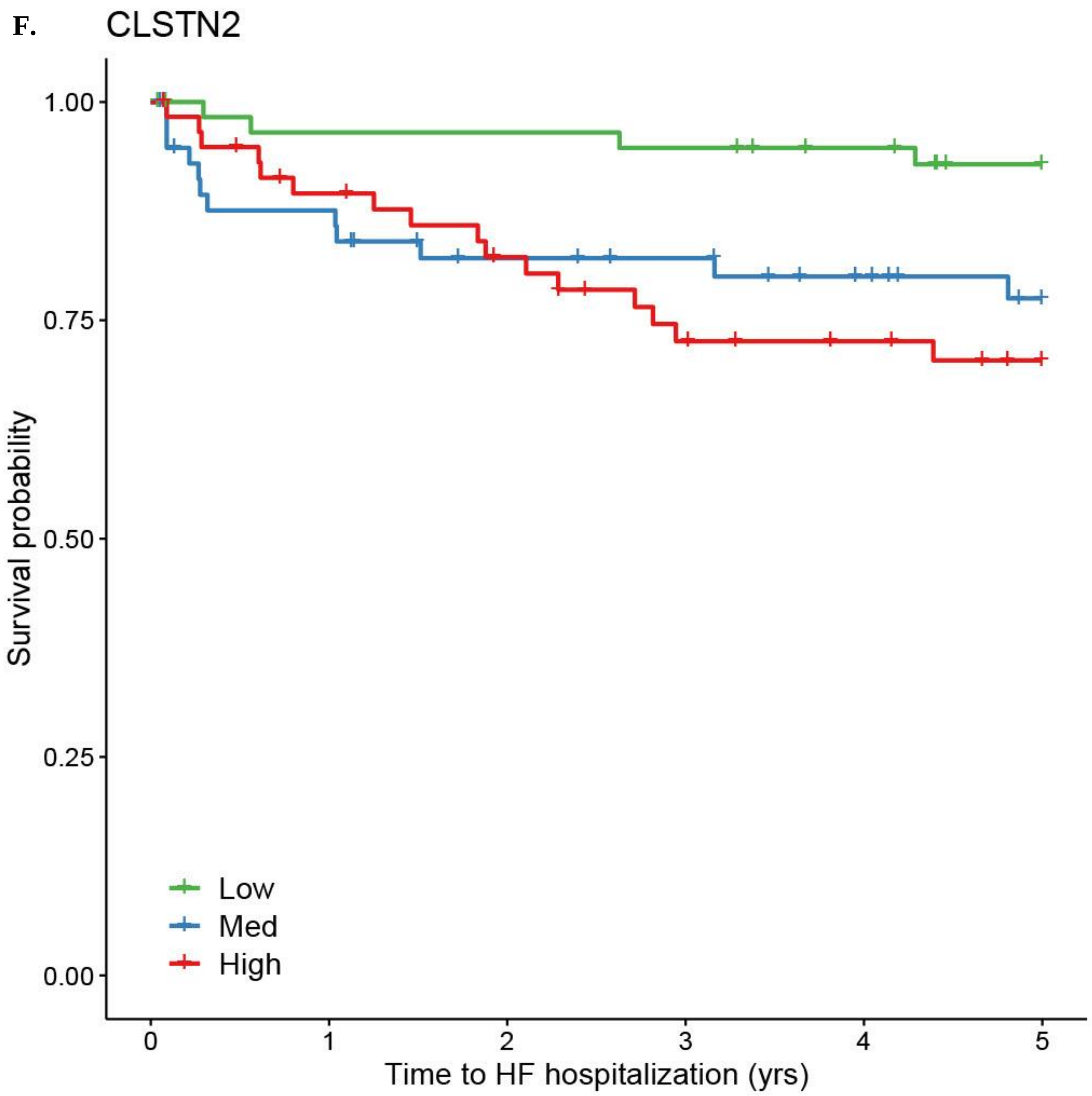
CLSTN2
1.00
0.75
0.50
0.25
0.00
Survival probability
0
1
2
3
4
5
Time to HF hospitalization (yrs)
Low
Med
High

G.

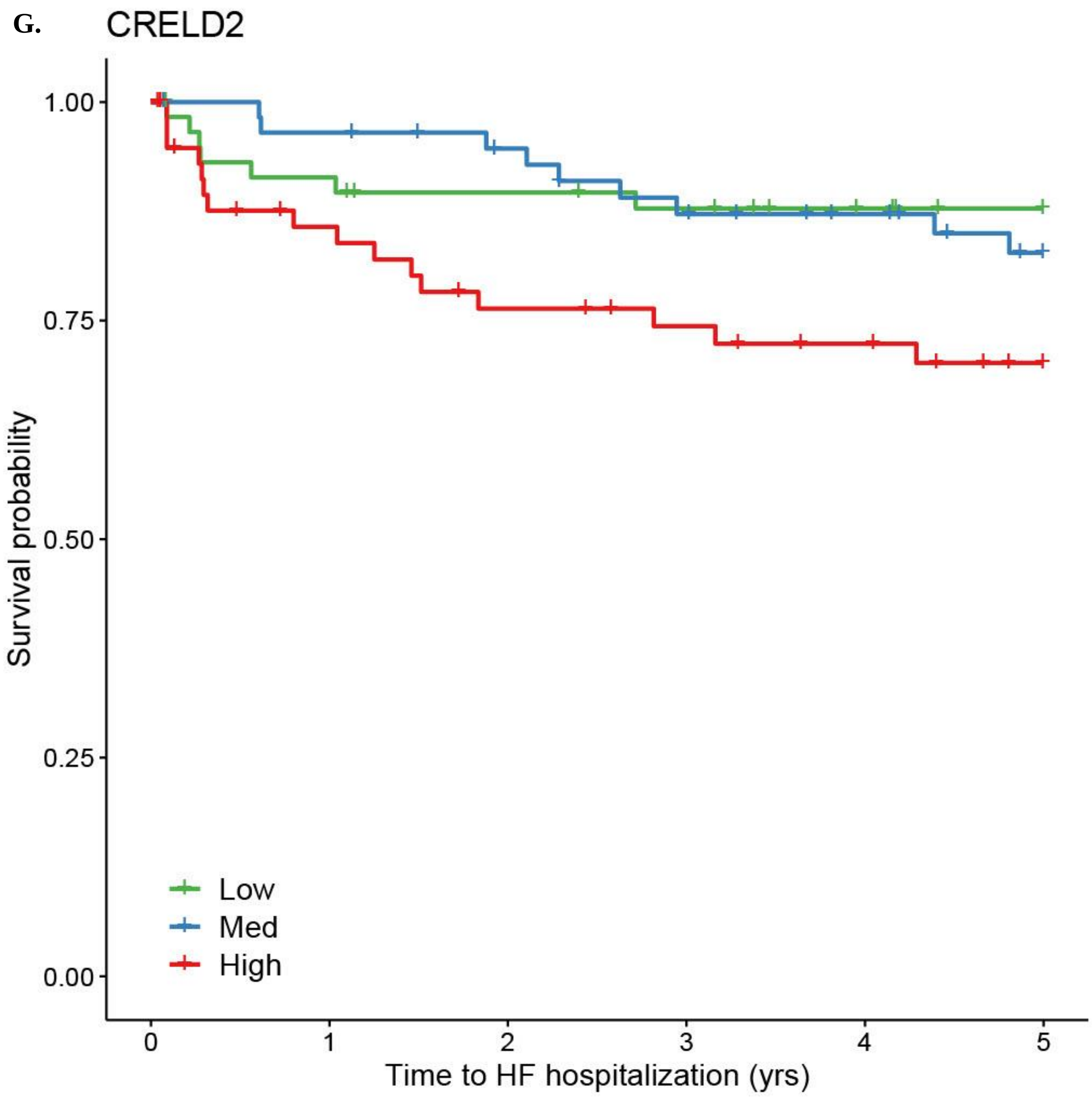

CRELD2
1.00
0.75
0.50
0.25
0.00
Survival probability
0
1
2
3
4
5
Time to HF hospitalization (yrs)
Low
Med
High

H.

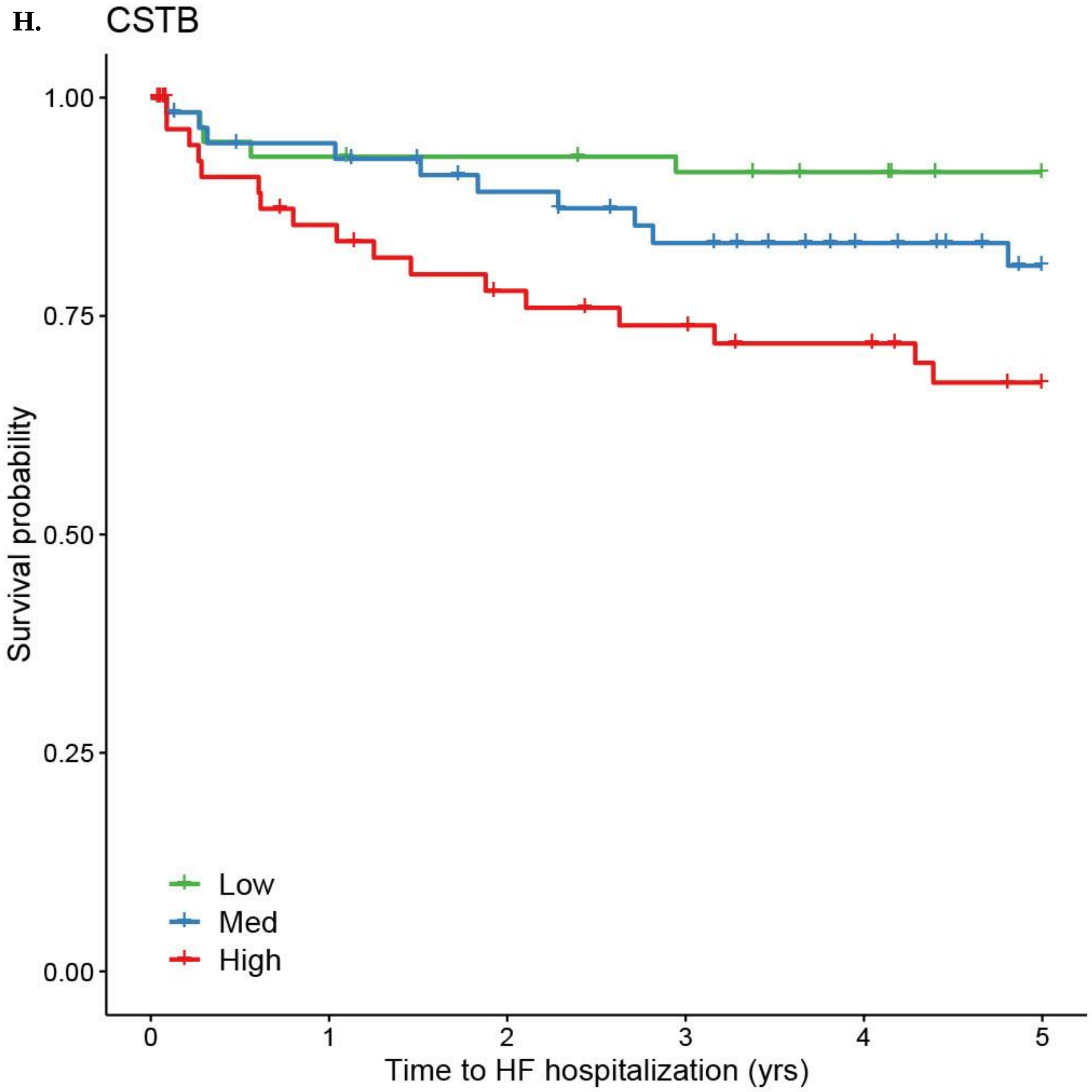
CSTB
1.00
0.75
0.50
0.25
0.00
Survival probability
0
1
2
3
4
5
Time to HF hospitalization (yrs)
Low
Med
High

I.

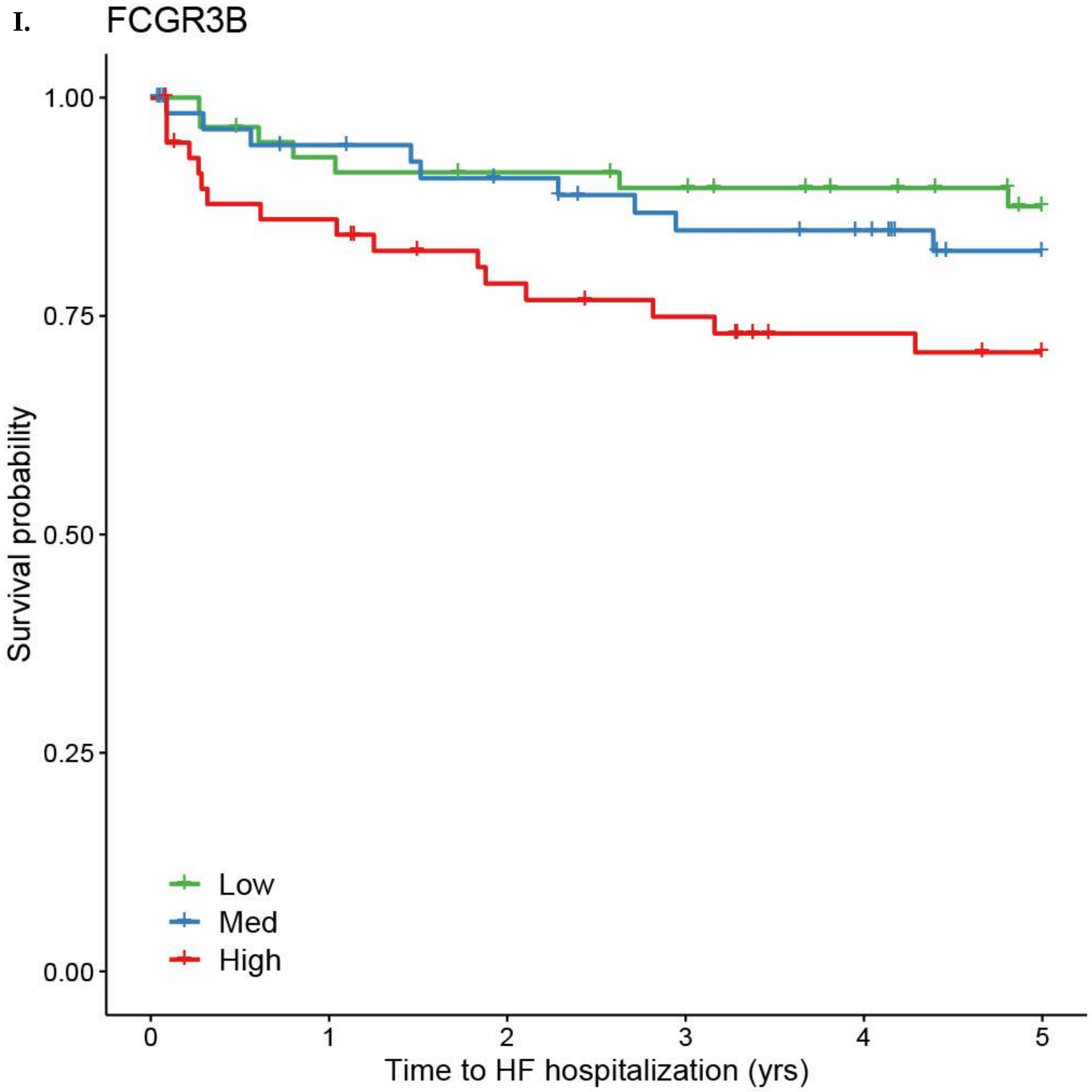
FCGR3B
Survival probability
1.00
0.75
0.50
0.25
0.00
0
1
2
3
4
5
Time to HF hospitalization (yrs)
Low
Med
High

J.

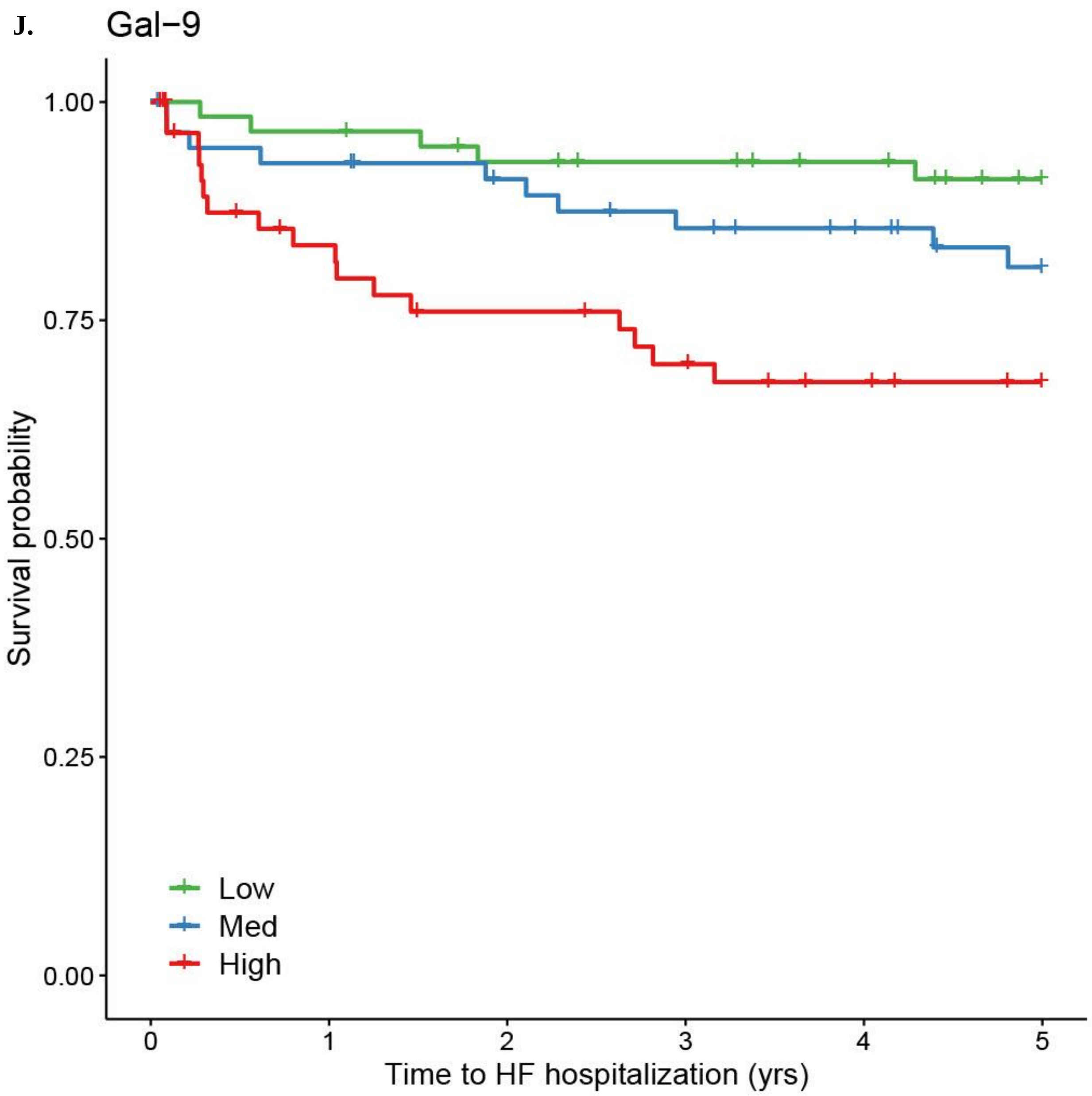

Gal−9
Survival probability
1.00
0.75
0.50
0.25
0.00
0
1
2
3
4
5
Time to HF hospitalization (yrs)
Low
Med
High

K.

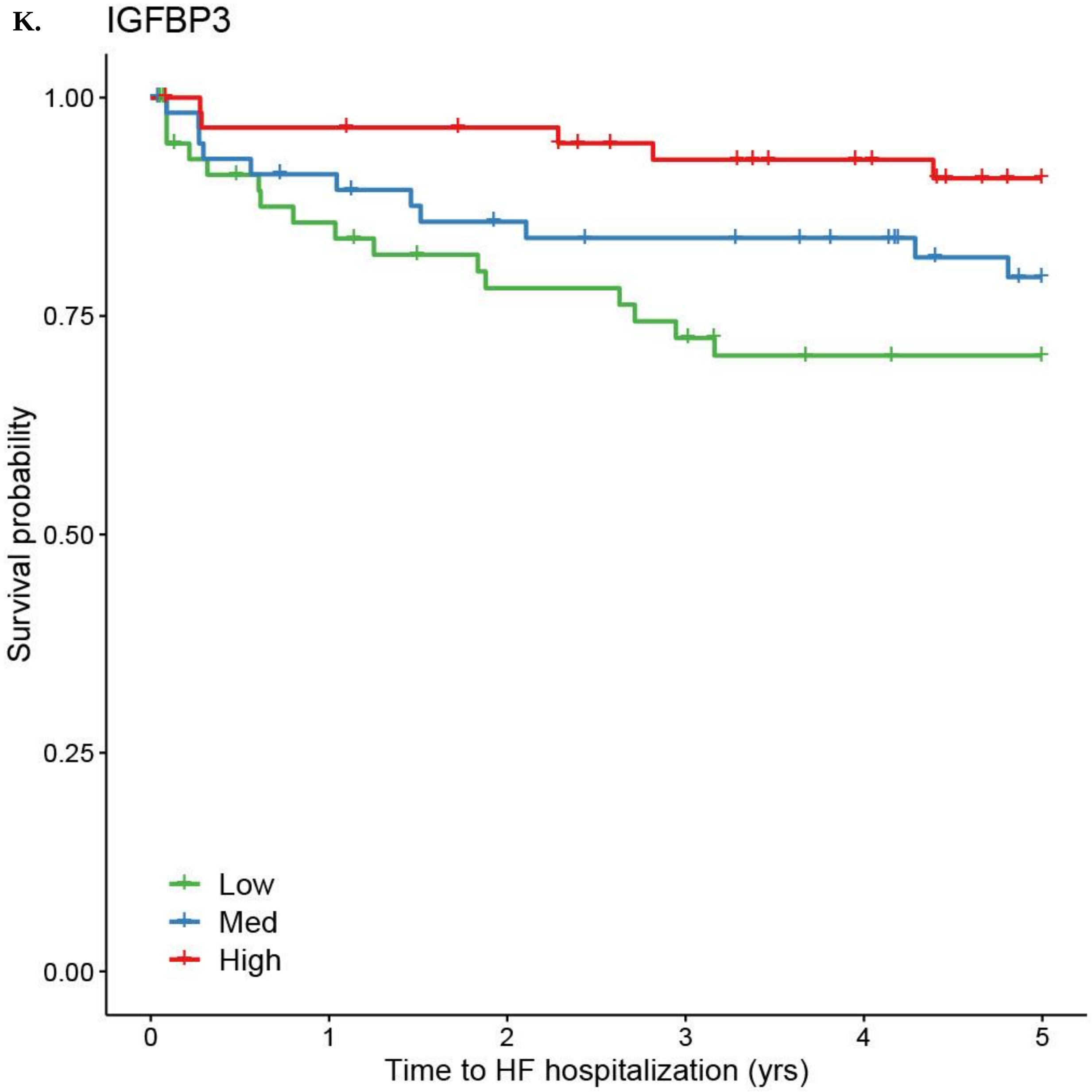
IGFBP3
1.00
0.75
0.50
0.25
0.00
Survival probability
0
1
2
3
4
5
Time to HF hospitalization (yrs)
Low
Med
High

L.

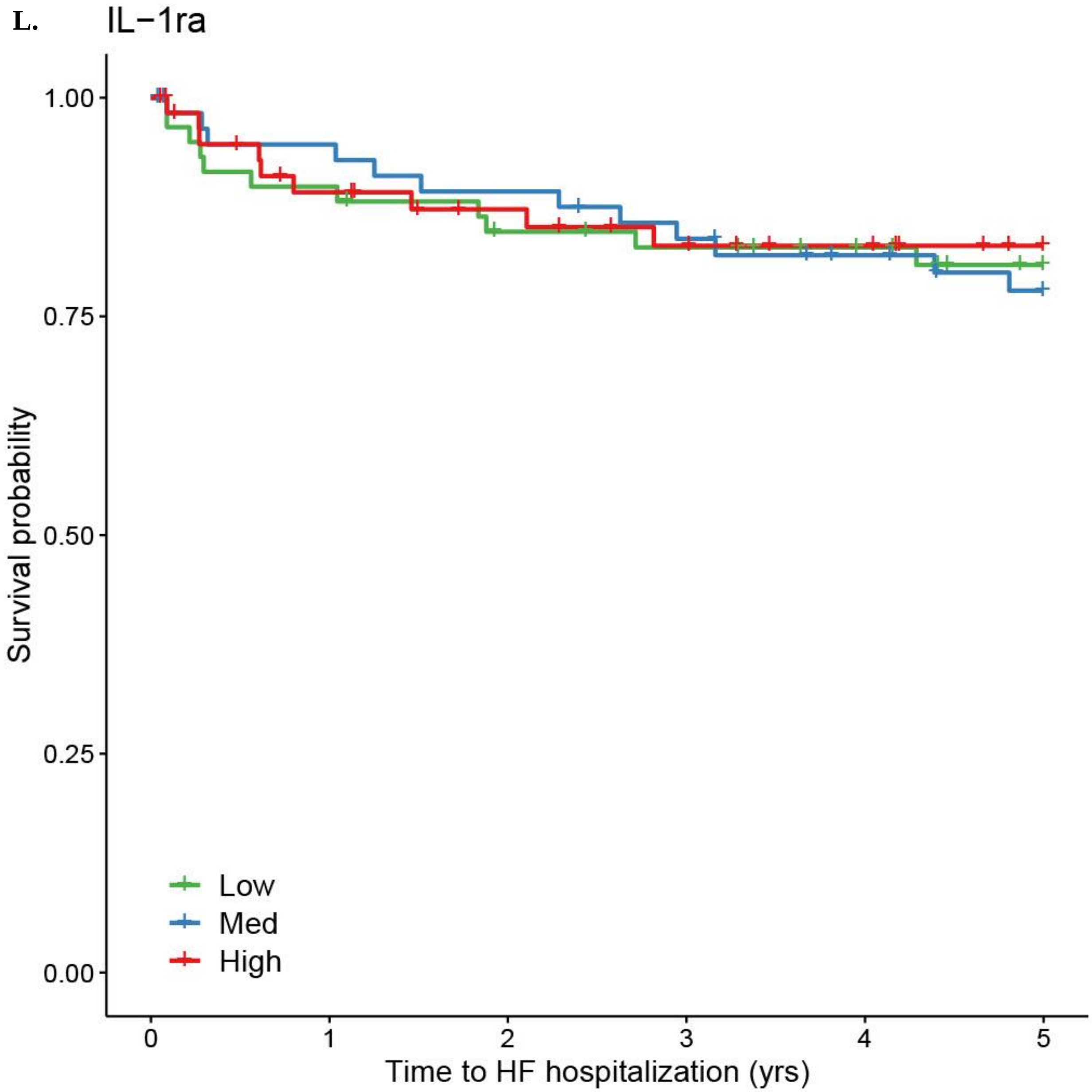

IL−1ra
1.00
0.75
0.50
0.25
0.00
Survival probability
0
1
2
3
4
5
Time to HF hospitalization (yrs)
Low
Med
High

M.

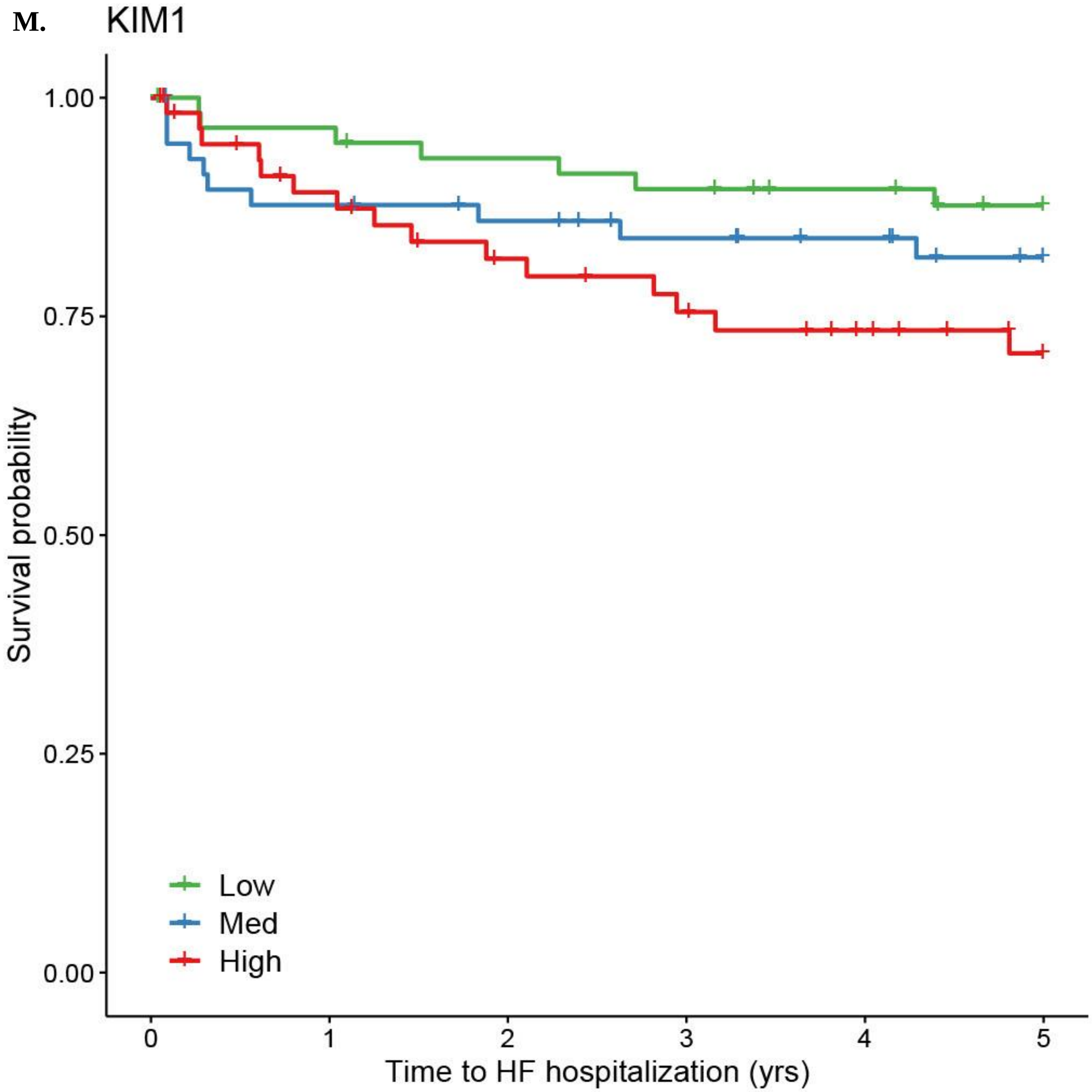
KIM1
1.00
0.75
0.50
0.25
0.00
Survival probability
0
1
2
3
4
5
Time to HF hospitalization (yrs)
Low
Med
High

N.

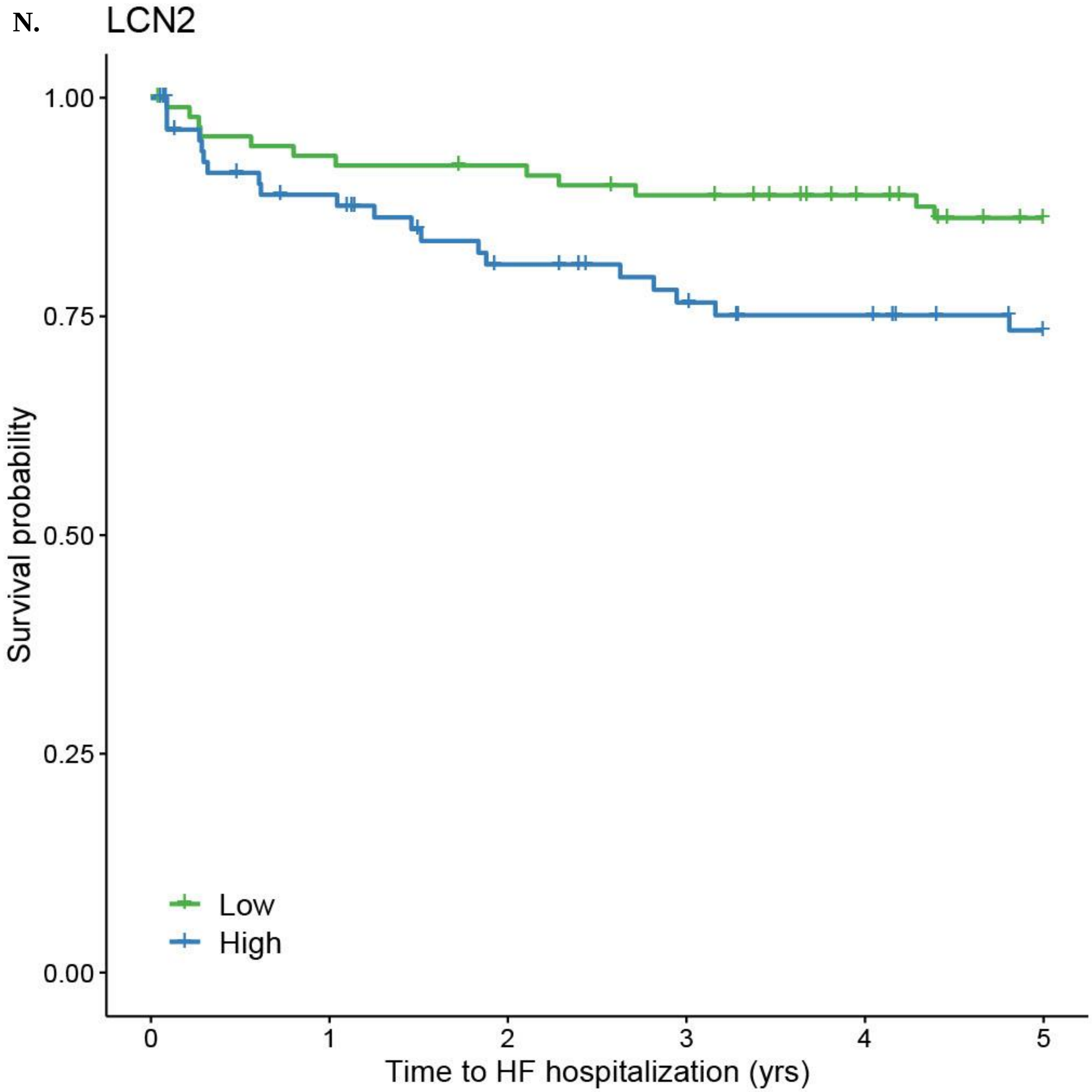
LCN2
1.00
0.75
0.50
0.25
0.00
Survival probability
0
1
2
3
4
5
Time to HF hospitalization (yrs)
Low
High

O. MATN2

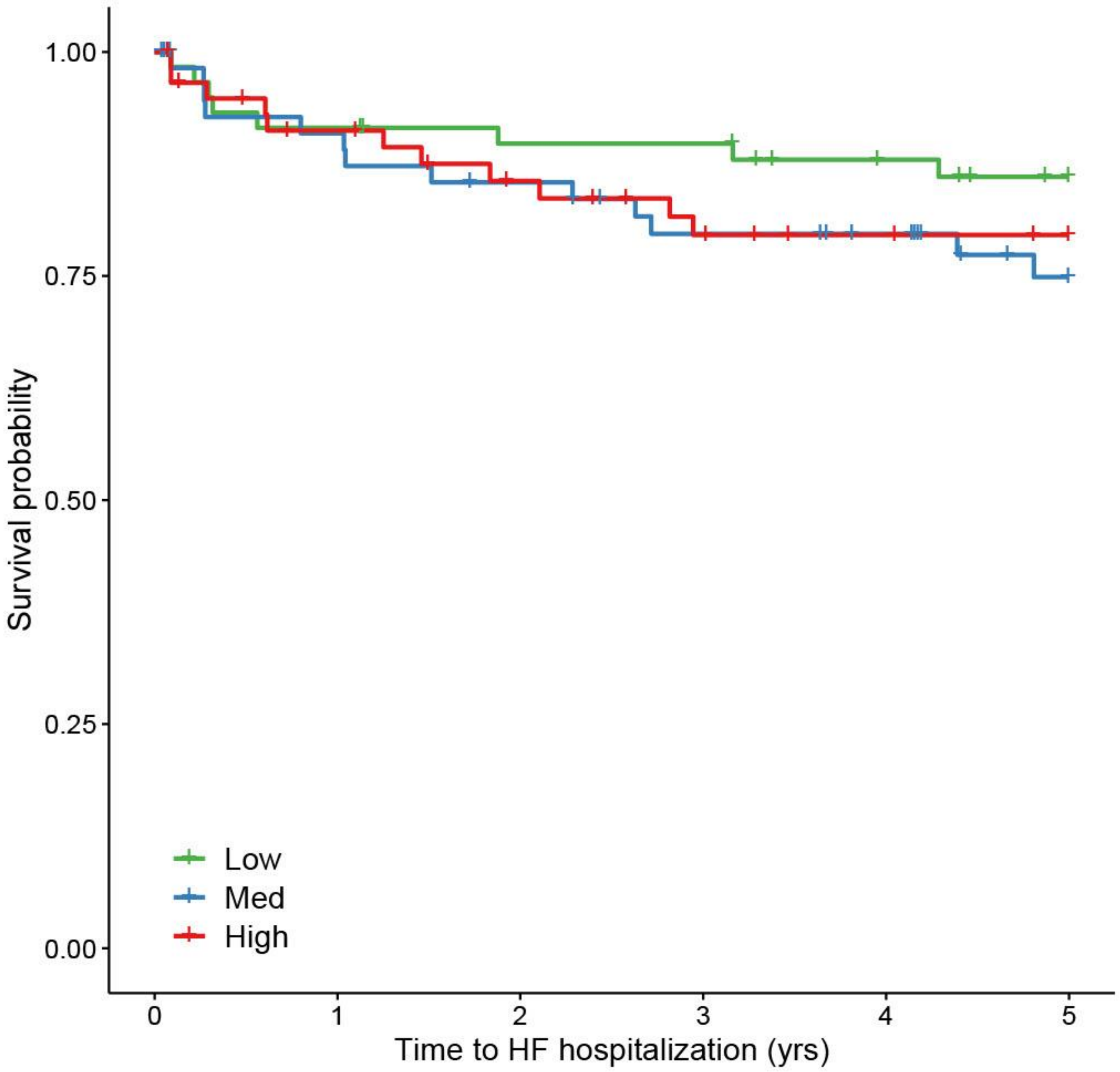

1.00
0.75
0.50
0.25
0.00
Survival probability
0
1
2
3
4
5
Time to HF hospitalization (yrs)
Low
Med
High

P.

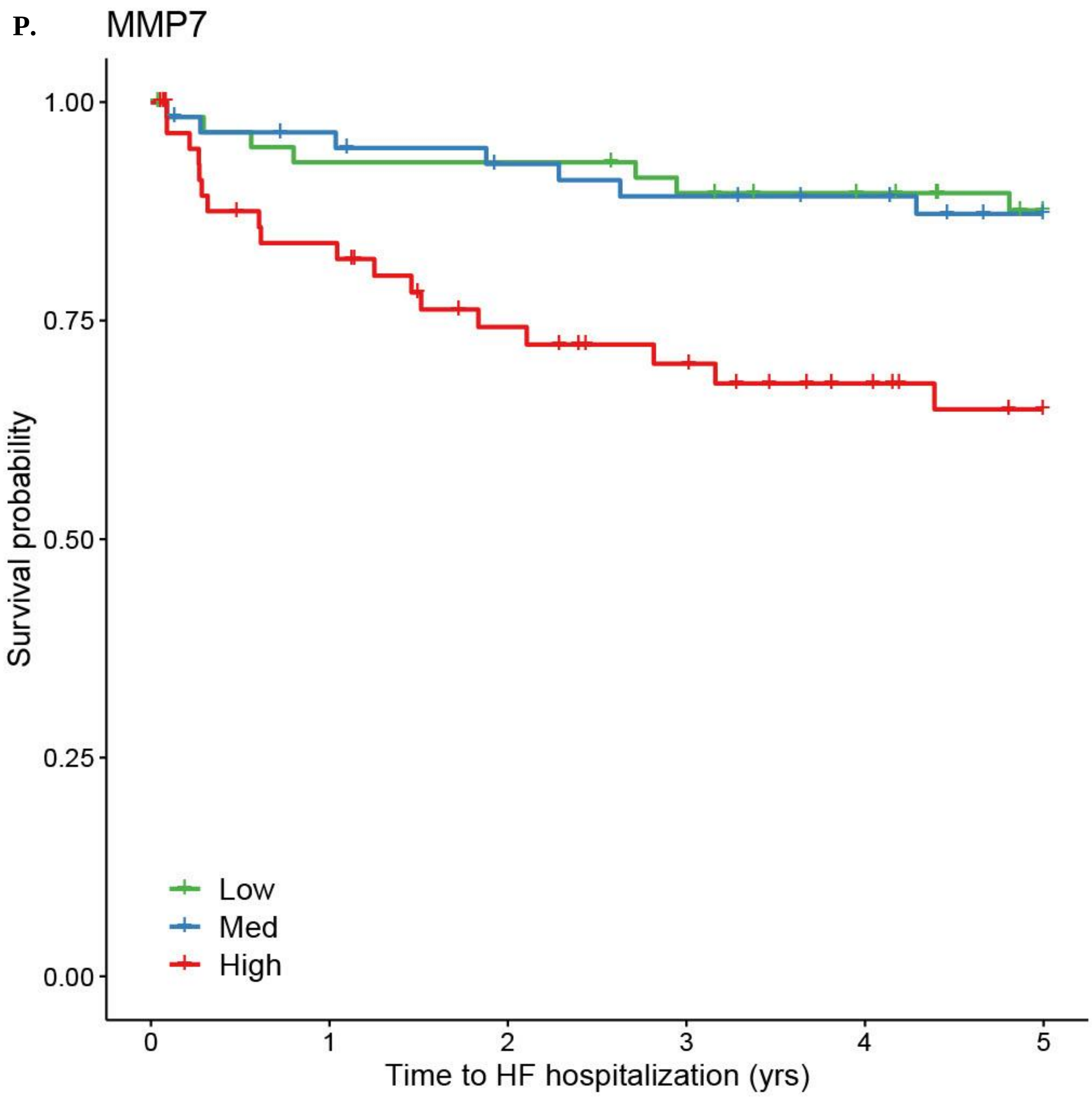
MMP7
Survival probability
1.00
0.75
0.50
0.25
0.00
0
1
2
3
4
5
Time to HF hospitalization (yrs)
Low
Med
High

Q.

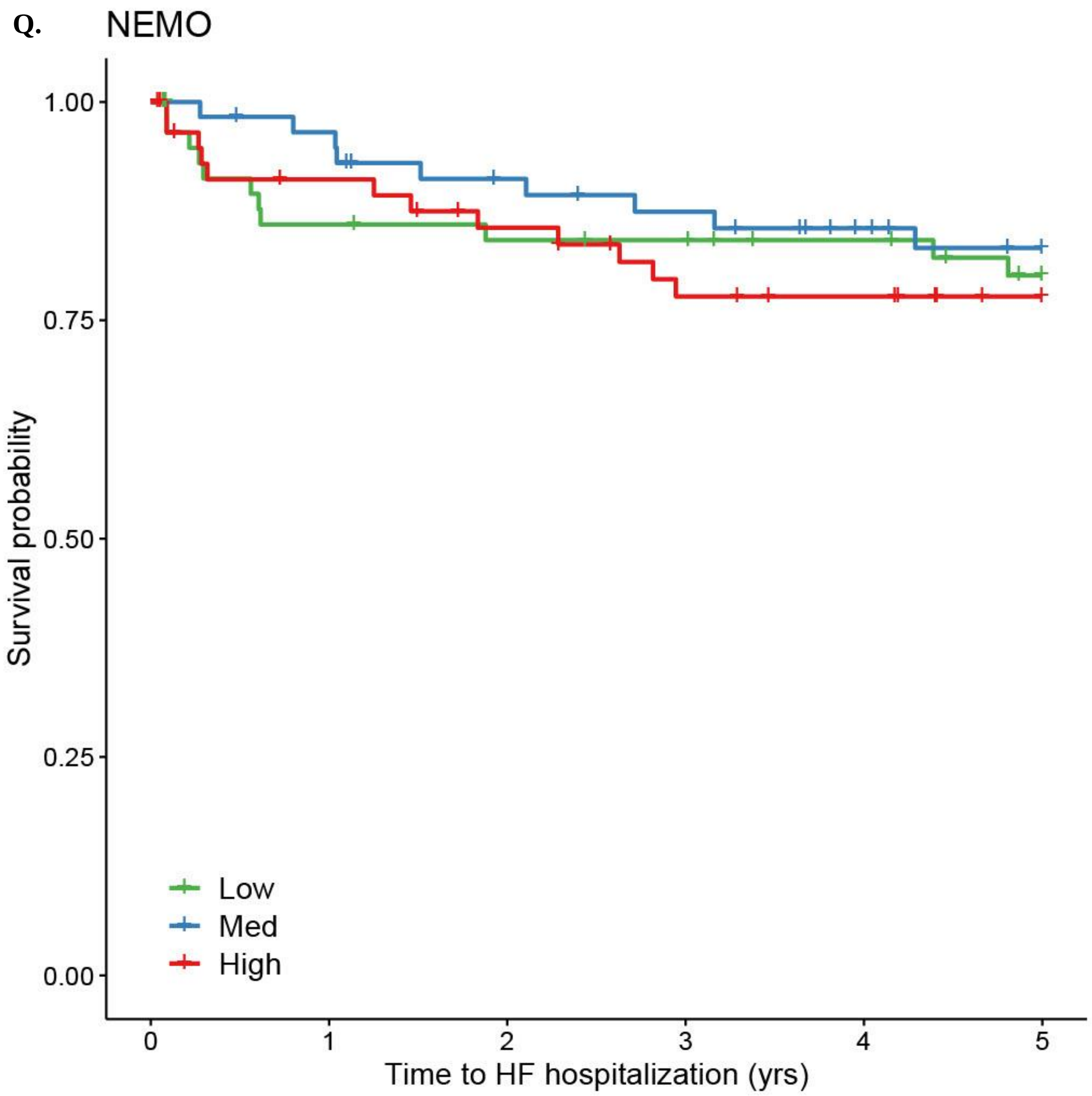
NEMO
1.00
0.75
0.50
0.25
0.00
Survival probability
0
1
2
3
4
5
Time to HF hospitalization (yrs)
Low
Med
High

R.

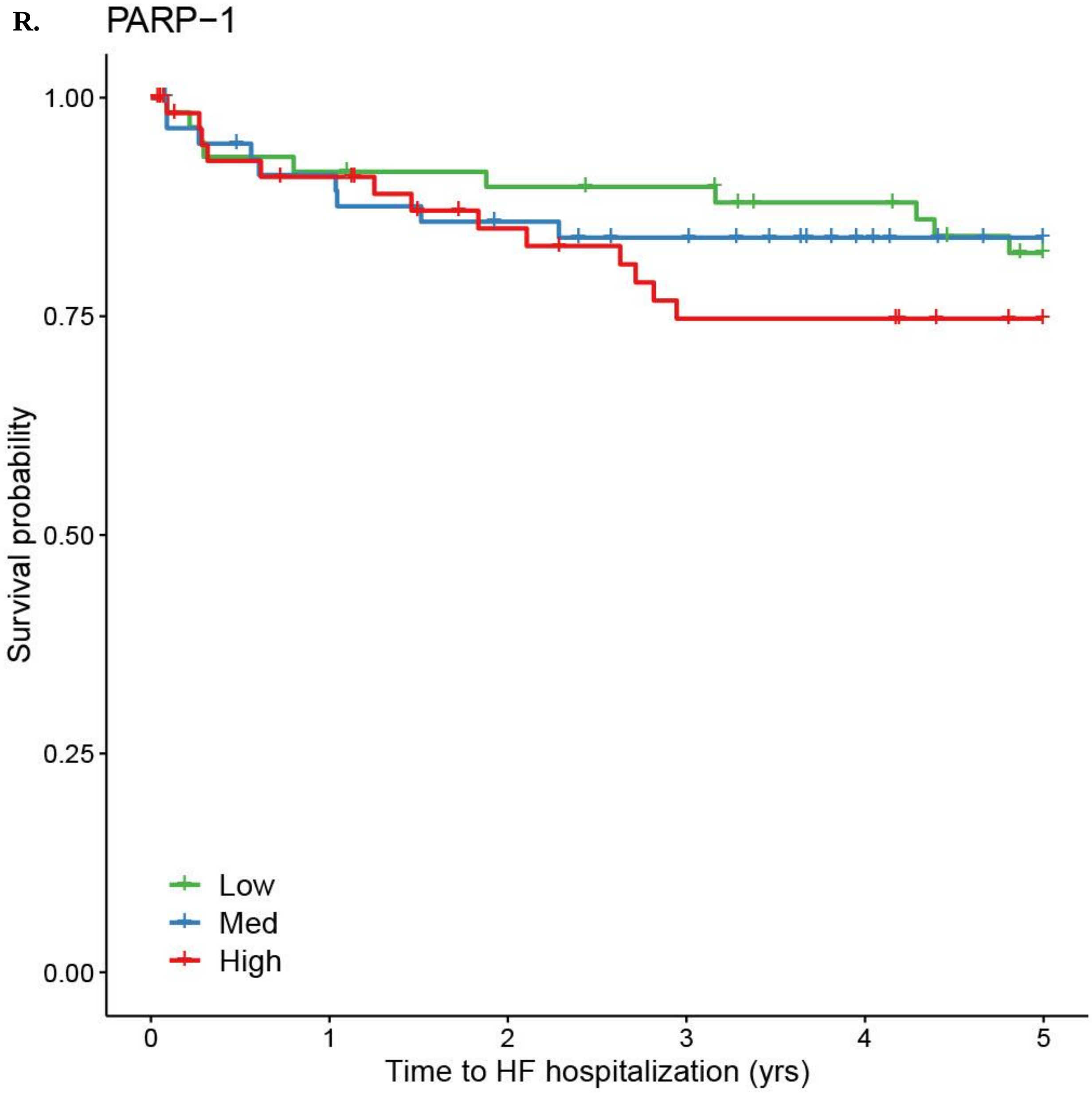
PARP−1
1.00
0.75
0.50
0.25
0.00
Survival probability
0
1
2
3
4
5
Time to HF hospitalization (yrs)
Low
Med
High

S.

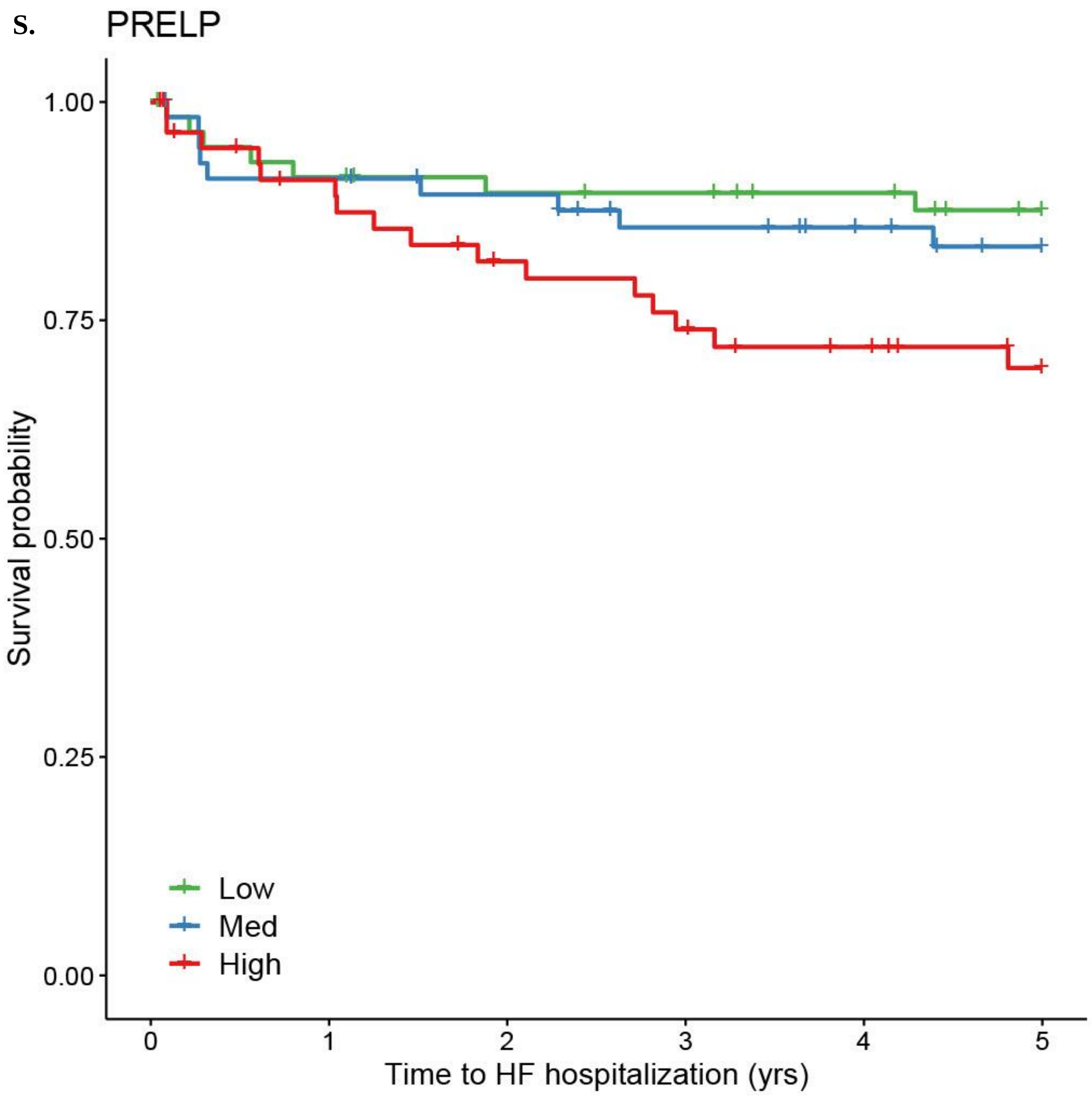
PRELP
1.00
0.75
0.50
0.25
0.00
Survival probability
0
1
2
3
4
5
Time to HF hospitalization (yrs)
Low
Med
High

T.

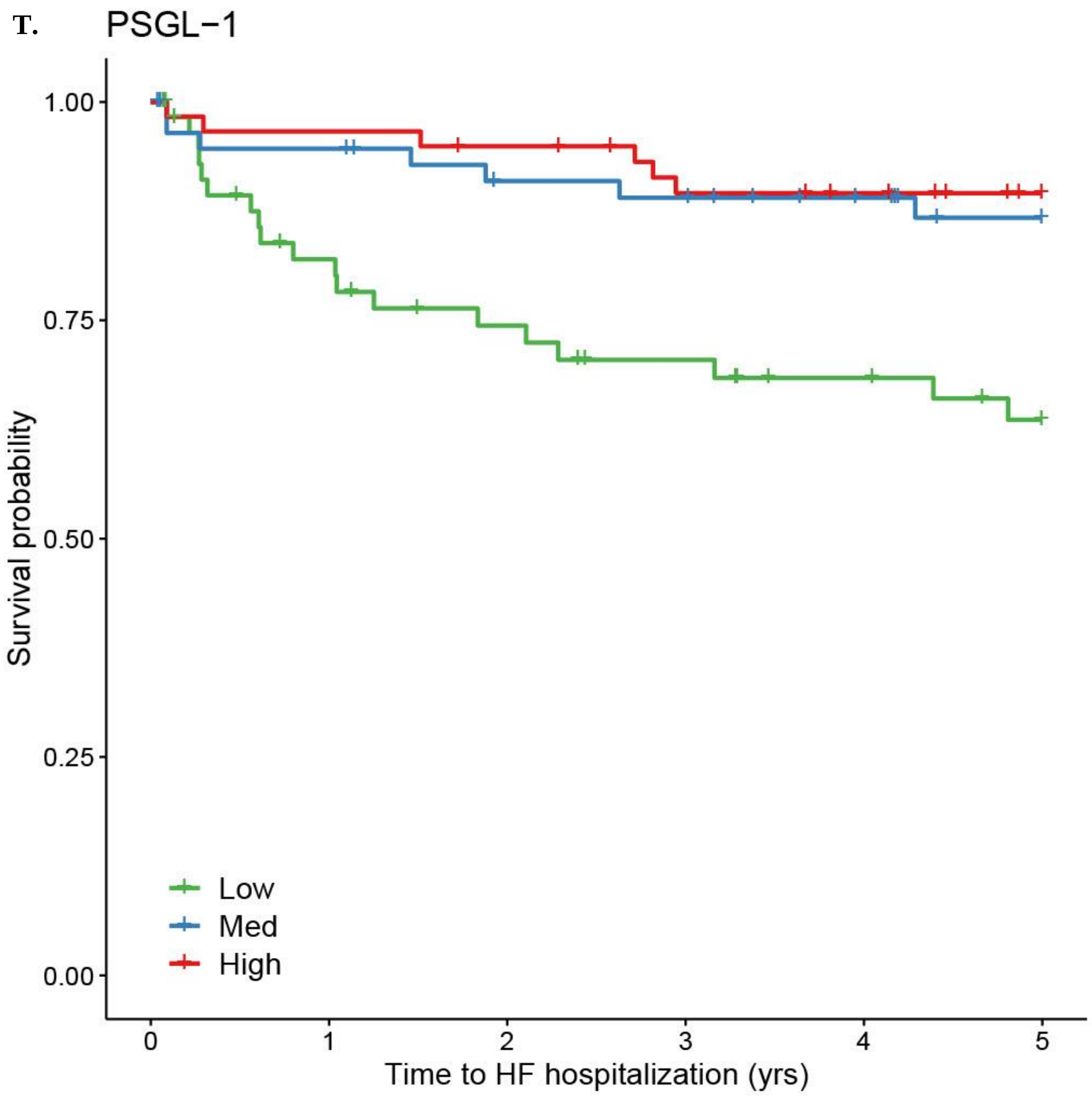
PSGL−1
Survival probability
1.00
0.75
0.50
0.25
0.00
0
1
2
3
4
5
Time to HF hospitalization (yrs)
Low
Med
High

U.

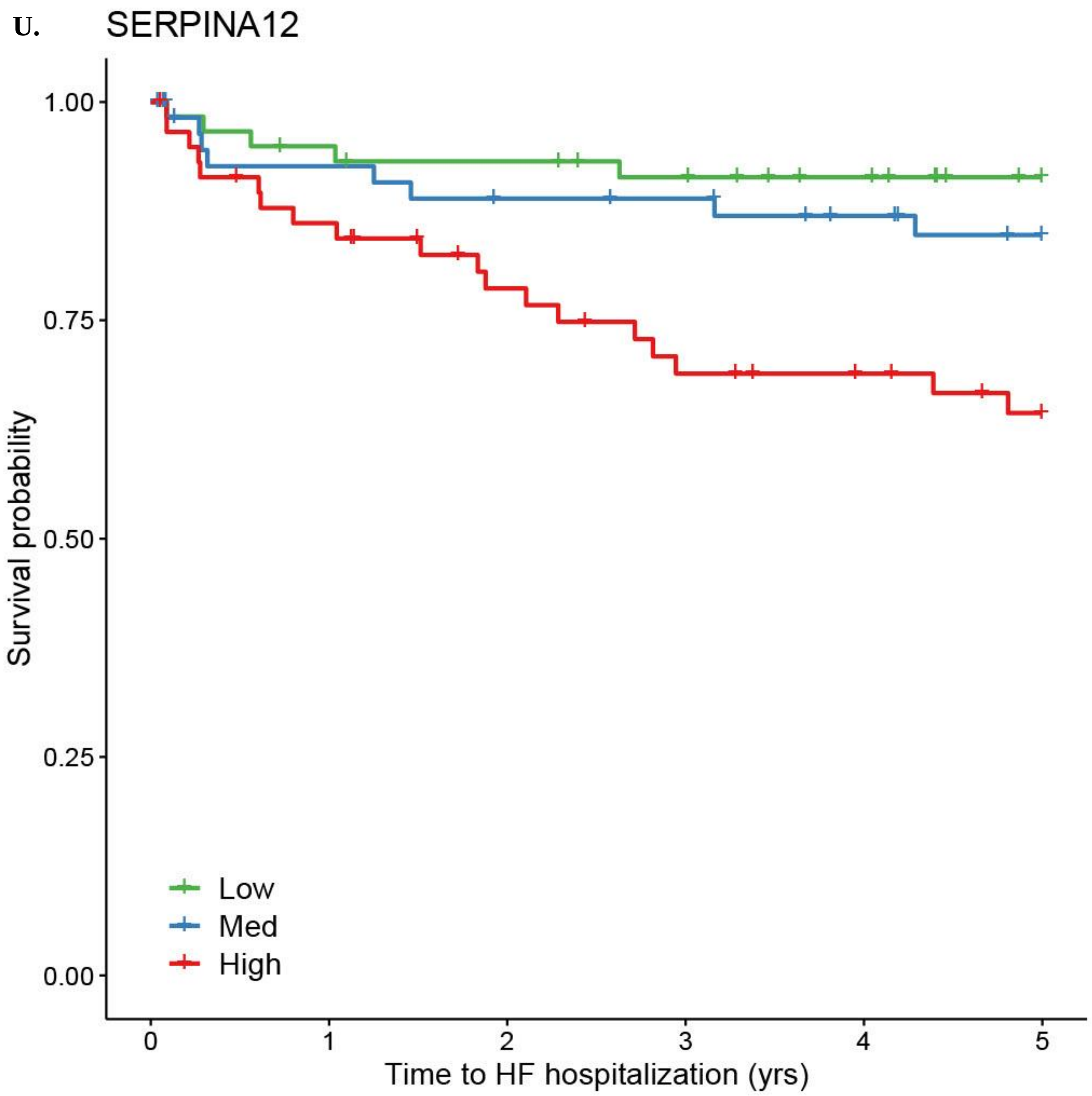

SERPINA12
Survival probability
1.00
0.75
0.50
0.25
0.00
Low
Med
High
0
1
2
3
4
5
Time to HF hospitalization (yrs)

v.

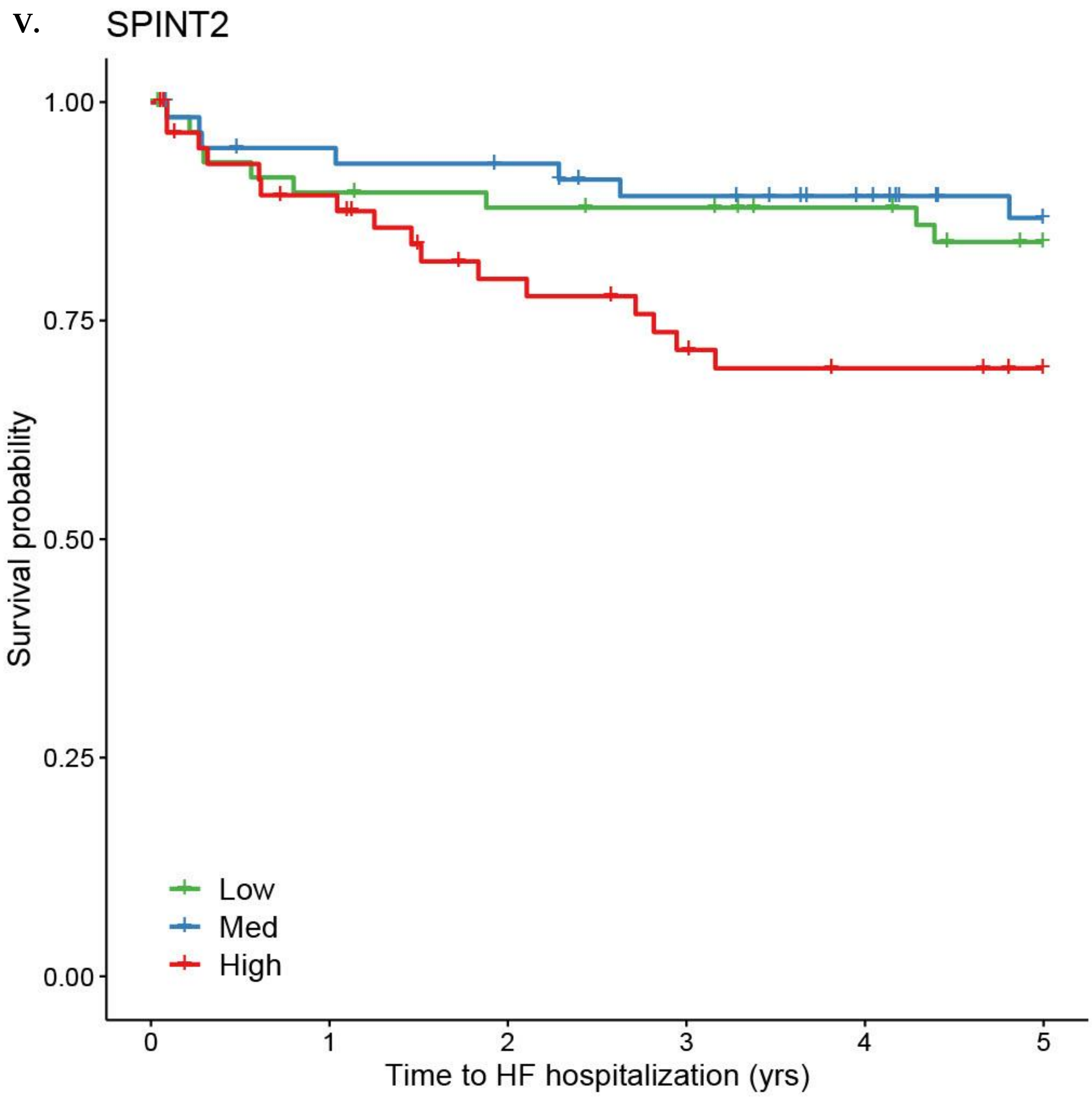

SPINT2
1.00
0.75
0.50
0.25
0.00
Survival probability
0
1
2
3
4
5
Time to HF hospitalization (yrs)
Low
Med
High

w.

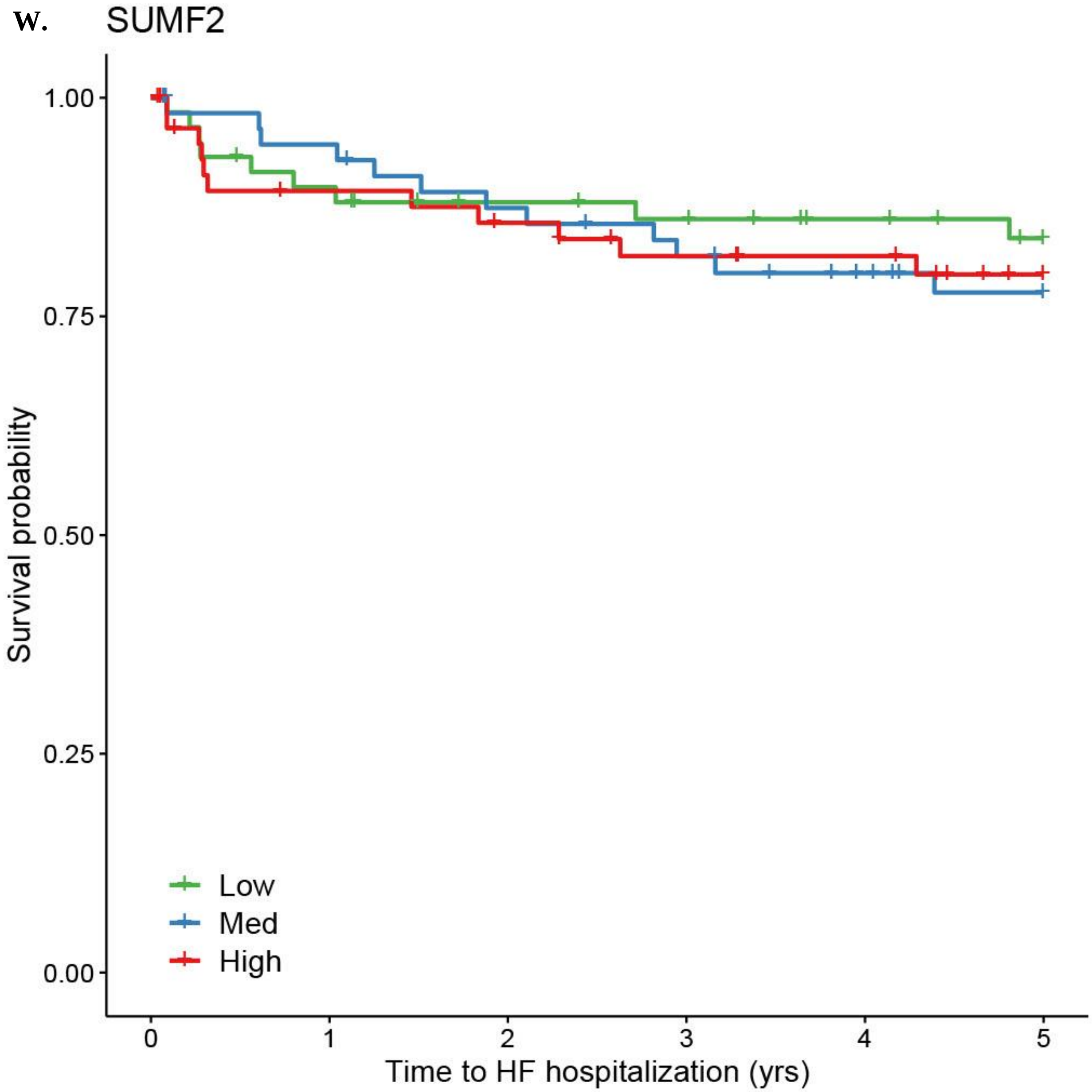
SUMF2
1.00
0.75
0.50
0.25
0.00
Survival probability
0
1
2
3
4
5
Time to HF hospitalization (yrs)
Low
Med
High

x. TIMP1

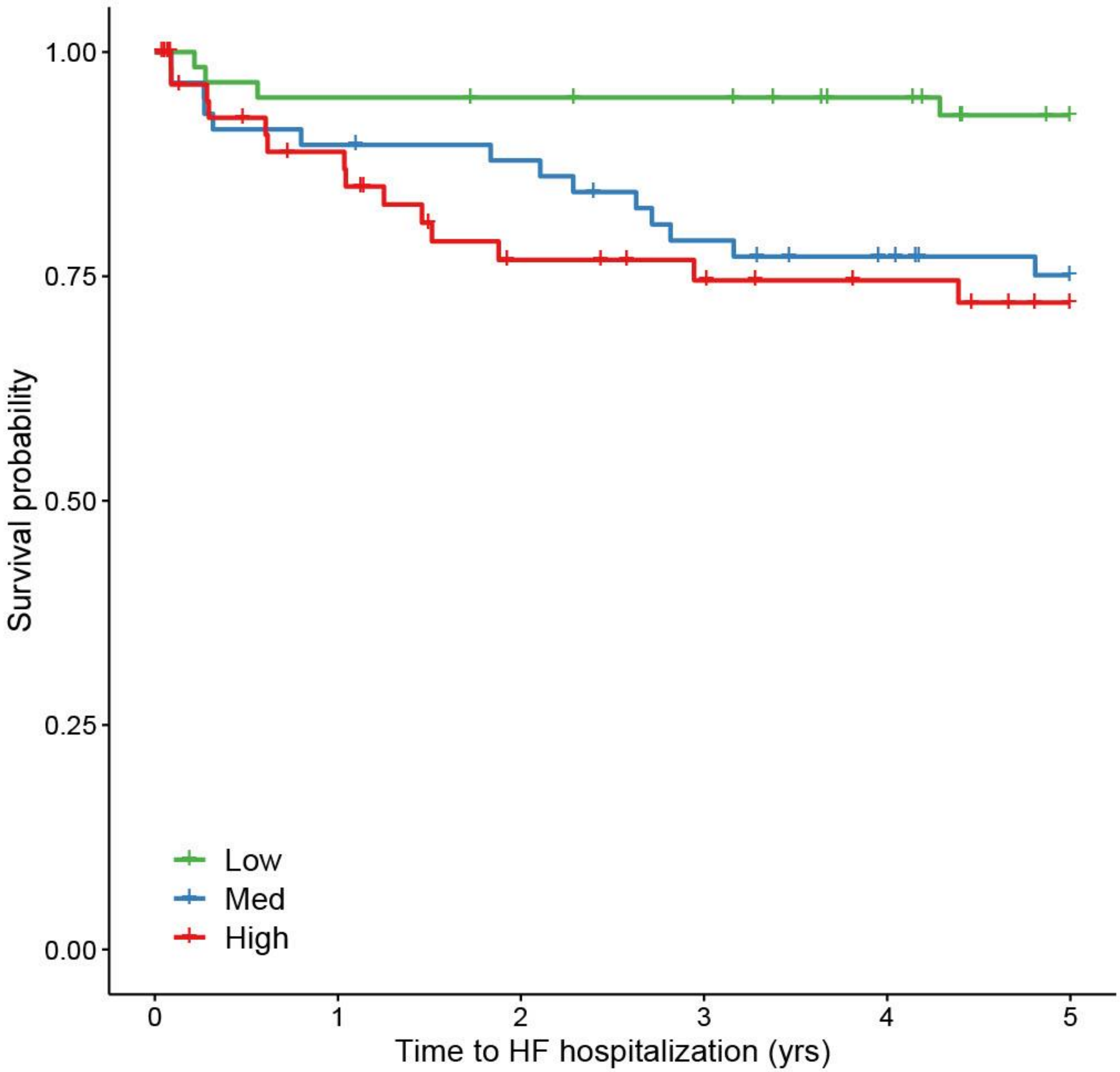

1.00
0.75
0.50
0.25
0.00
Survival probability
0
1
2
3
4
5
Time to HF hospitalization (yrs)
Low
Med
High

Y.

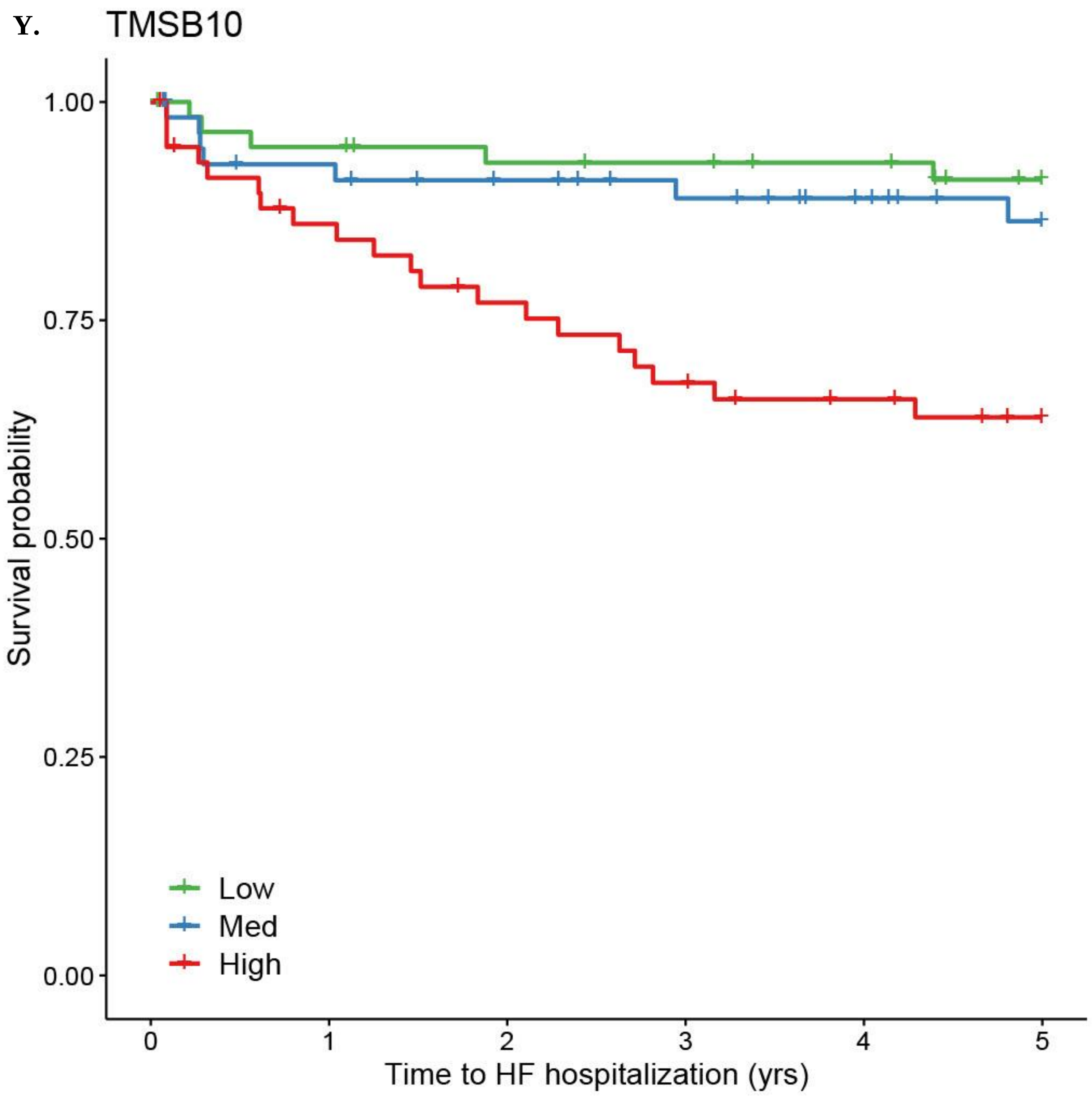
TMSB10
Survival probability
1.00
0.75
0.50
0.25
0.00
Low
Med
High
0
1
2
3
4
5
Time to HF hospitalization (yrs)

z.

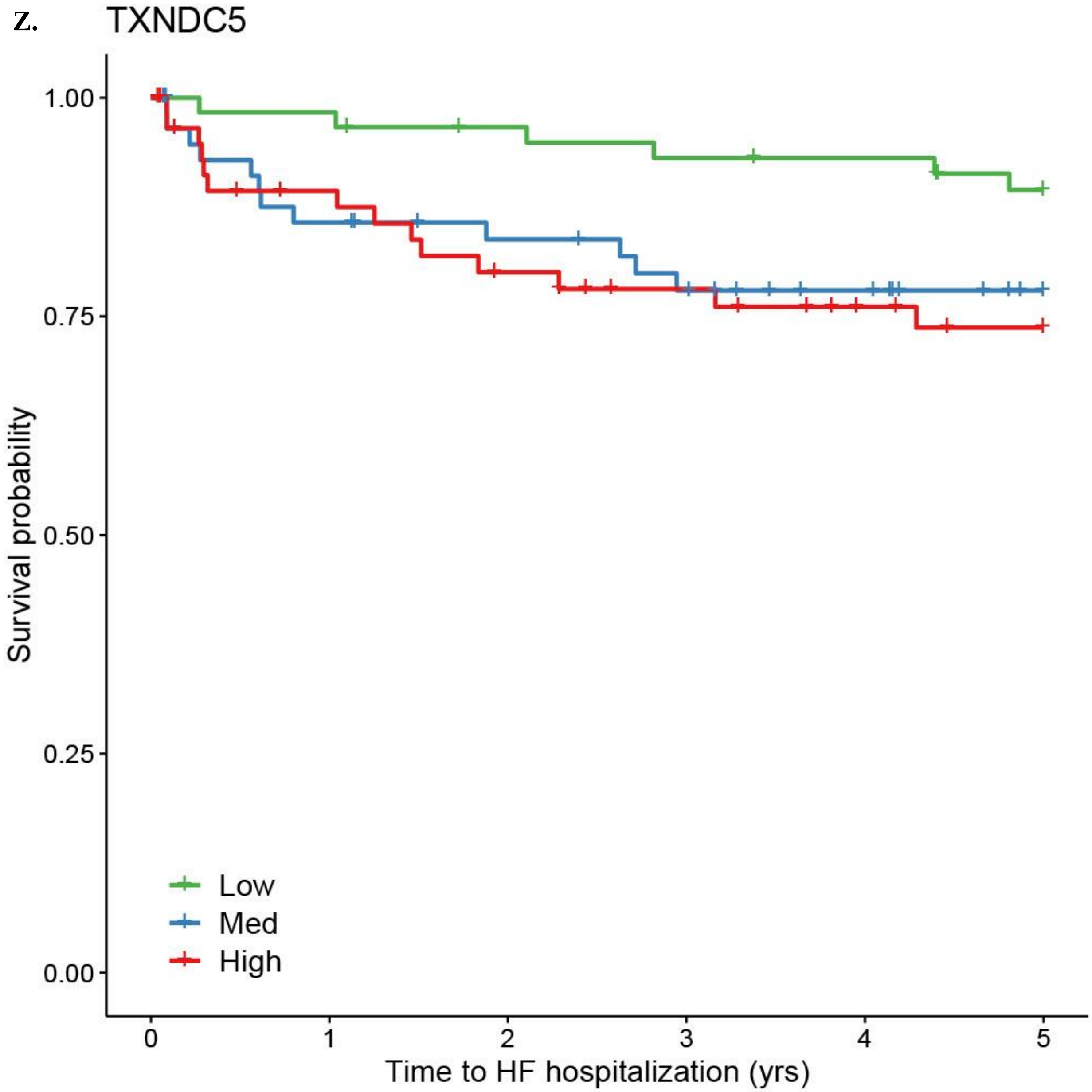
TXNDC5
Survival probability
1.00
0.75
0.50
0.25
0.00
0
1
2
3
4
5
Time to HF hospitalization (yrs)
Low
Med
High

AA.

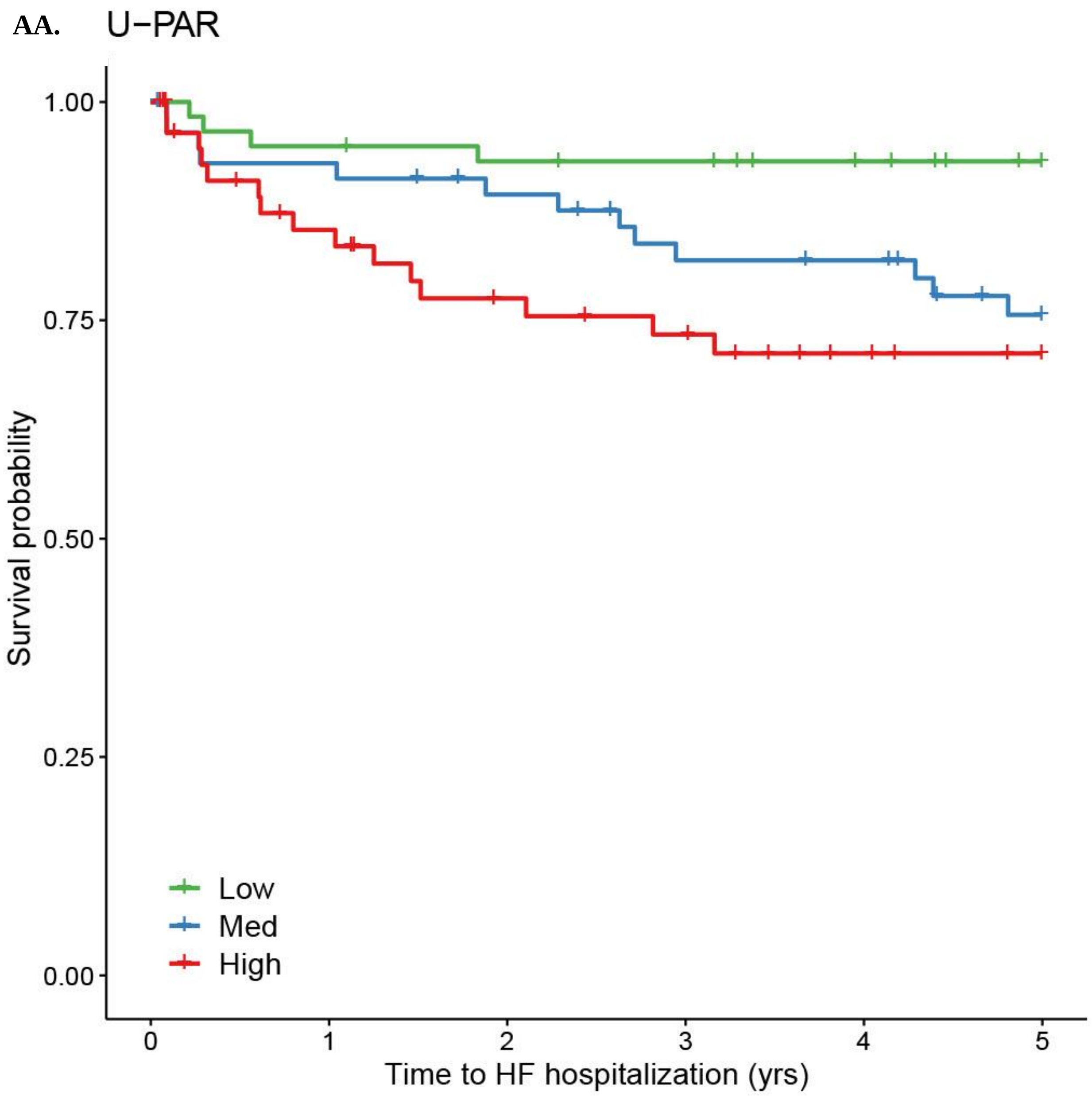
U−PAR
1.00
0.75
0.50
0.25
0.00
Survival probability
0
1
2
3
4
5
Time to HF hospitalization (yrs)
Low
Med
High

AB.

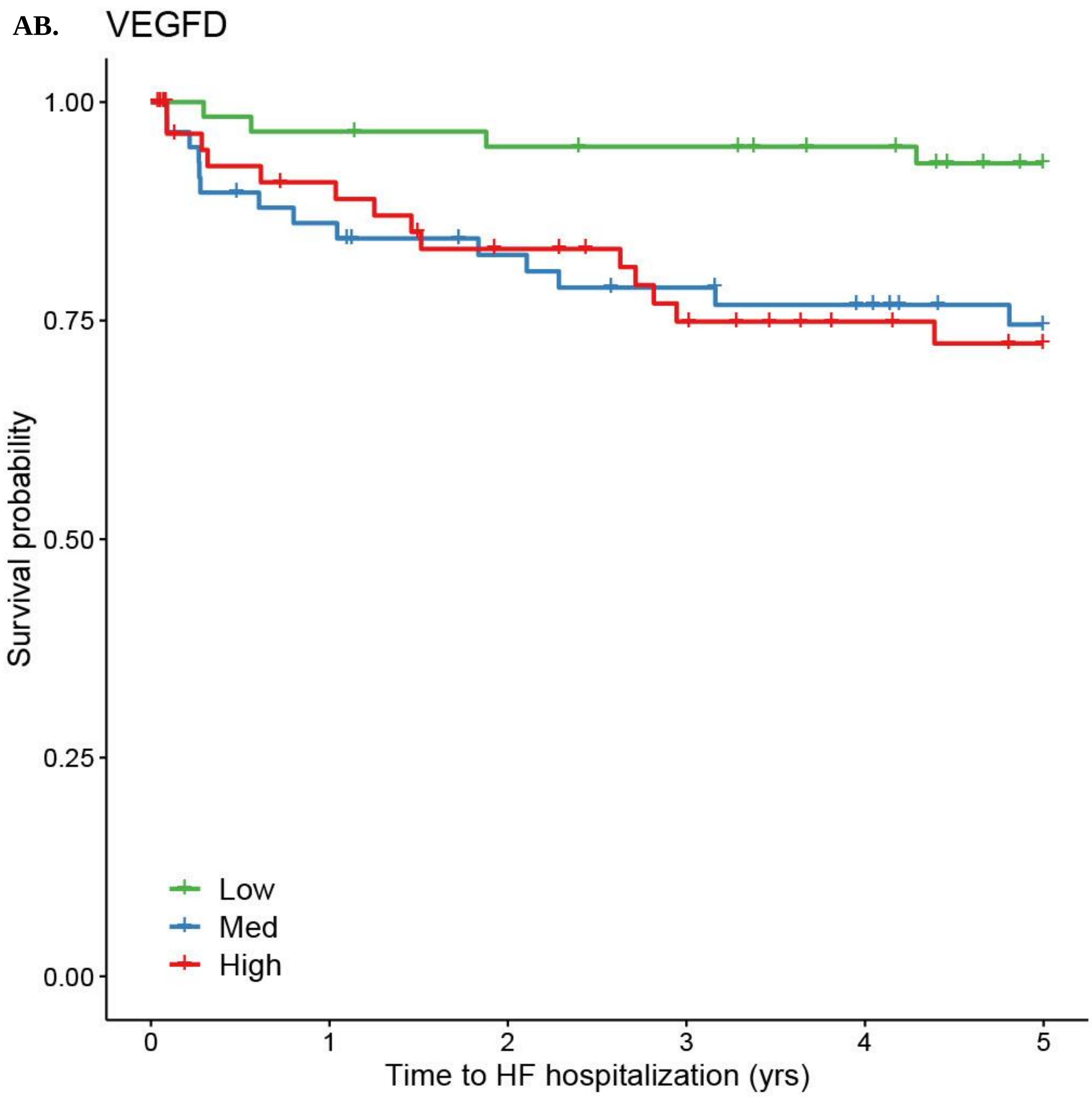
VEGFD
1.00
0.75
0.50
0.25
0.00
Survival probability
0
1
2
3
4
5
Time to HF hospitalization (yrs)
Low
Med
High

AC.

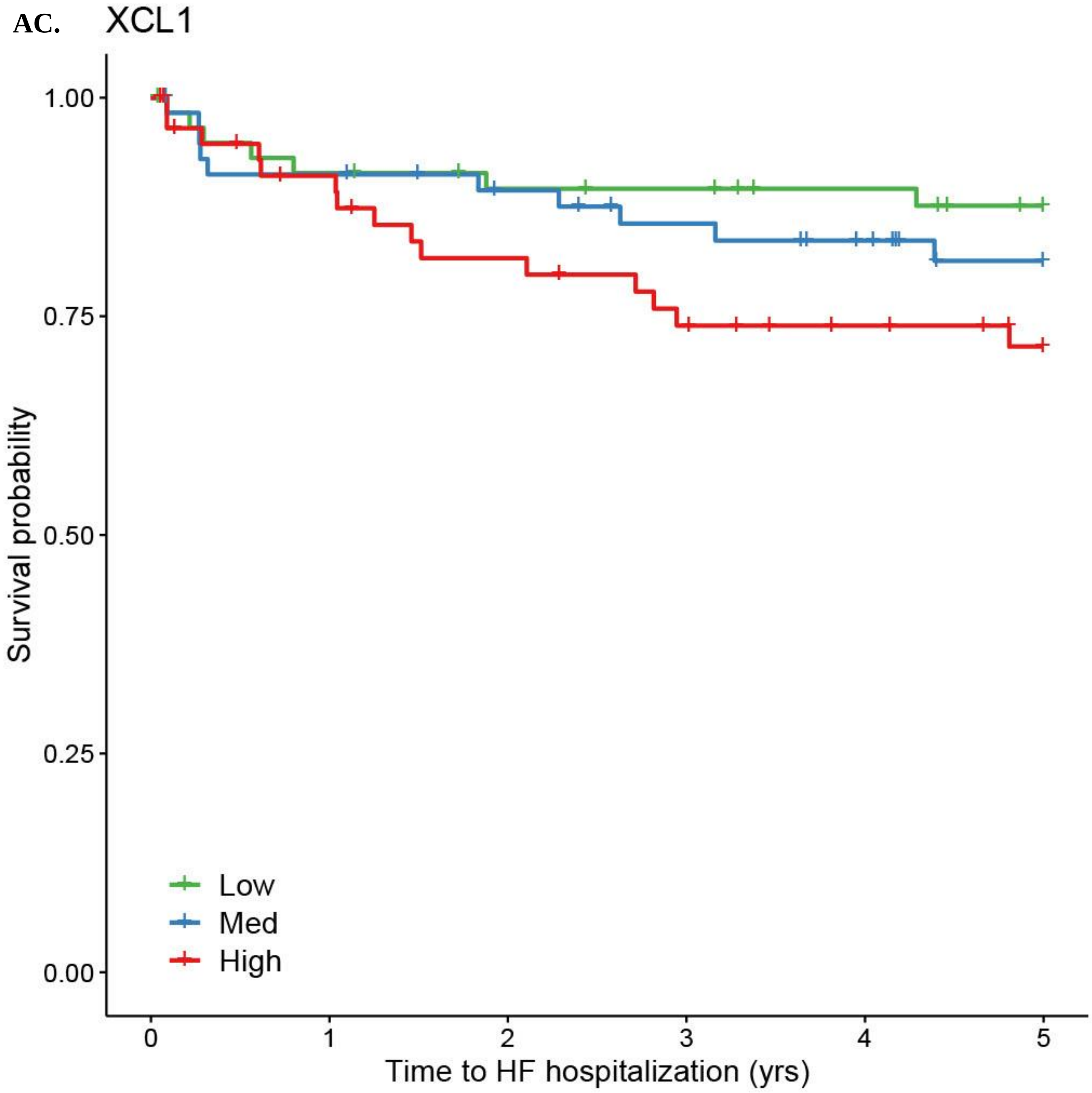
XCL1
1.00
0.75
0.50
0.25
0.00
Survival probability
0
1
2
3
4
5
Time to HF hospitalization (yrs)
Low
Med
High

Supplemental Figure 3. Kaplan-Meier curves for all-cause mortality by protein level in CATHGEN. Each curve shows time to all-cause mortality by protein level tertile in CATHGEN for the 29 proteins that were either selected by LASSO or validated in TECOS.

A.

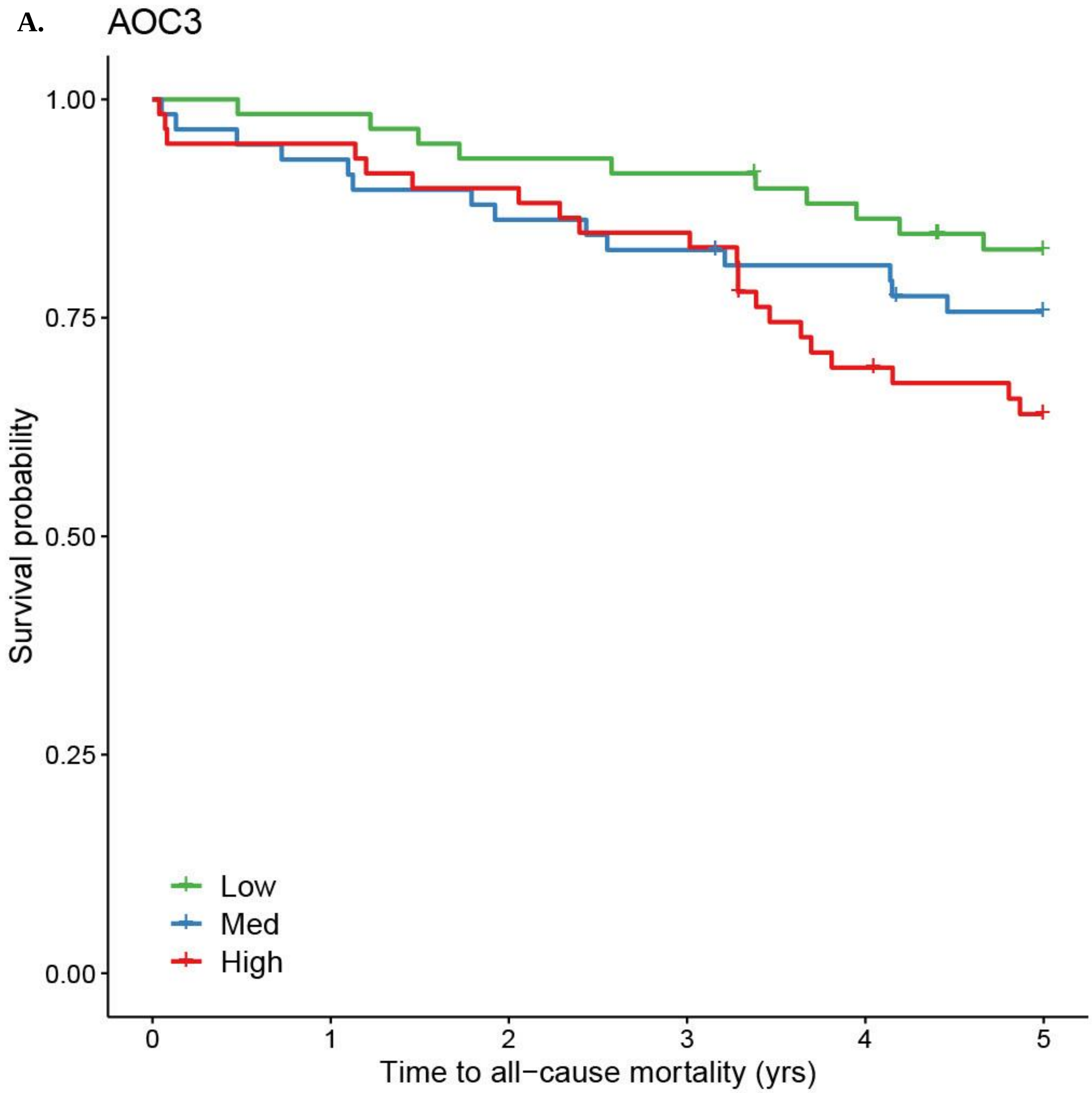

B.

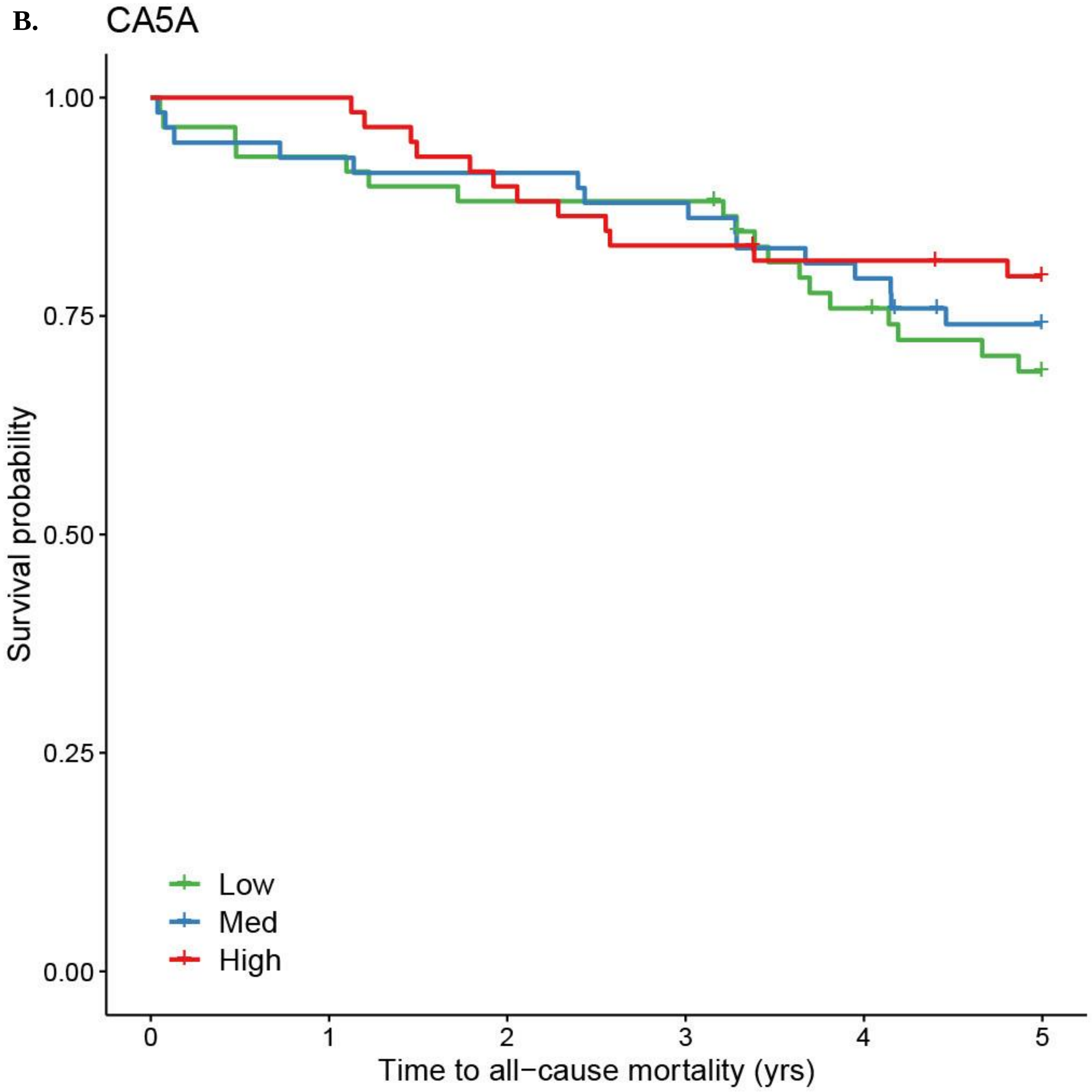

CA5A
1.00
0.75
0.50
0.25
0.00
Survival probability
0
1
2
3
4
5
Time to all−cause mortality (yrs)
Low
Med
High

C.

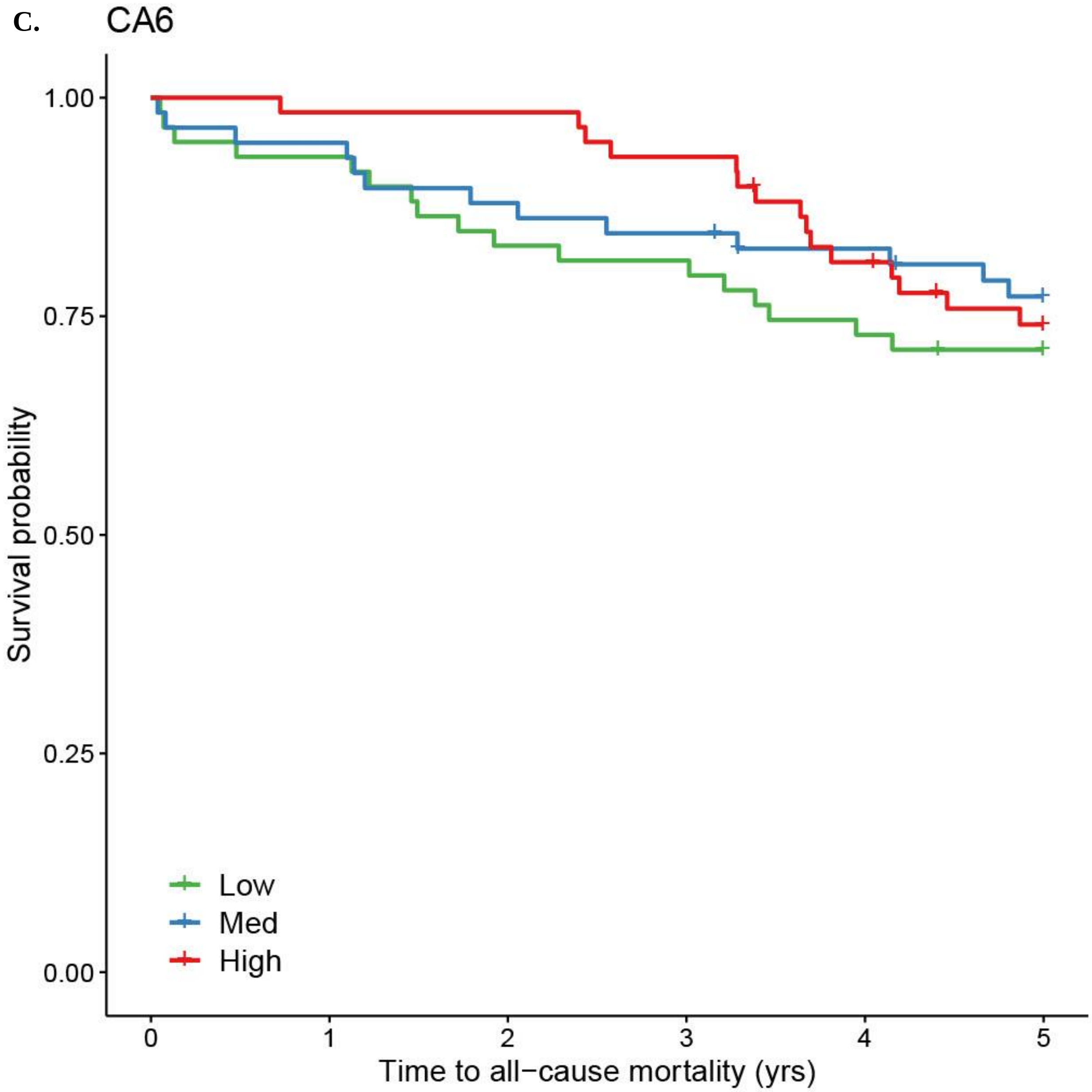
CA6
1.00
0.75
0.50
0.25
0.00
Survival probability
0
1
2
3
4
5
Time to all−cause mortality (yrs)
Low
Med
High

D.

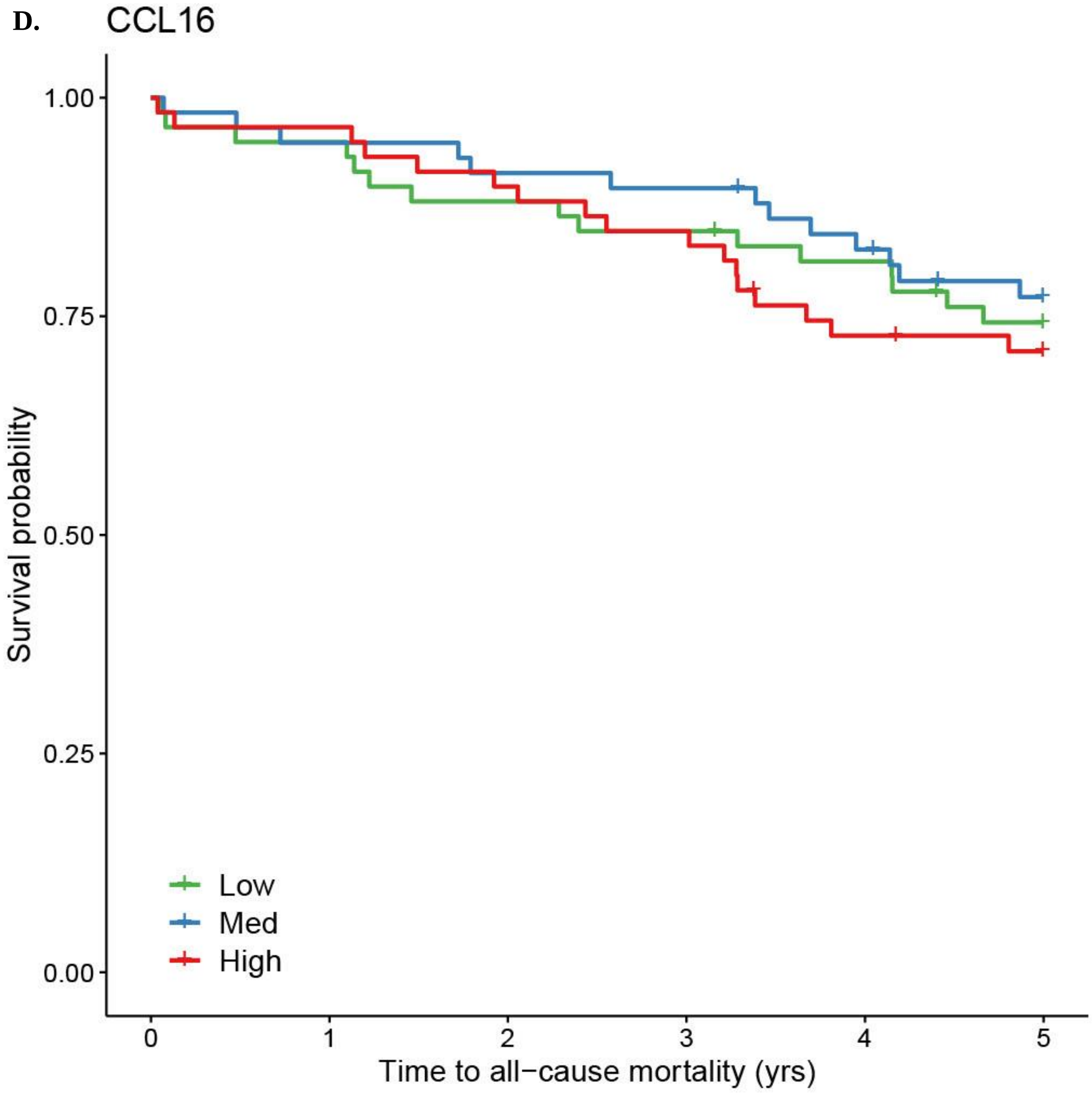
CCL16
1.00
0.75
0.50
0.25
0.00
Survival probability
0
1
2
3
4
5
Time to all−cause mortality (yrs)
Low
Med
High

E.

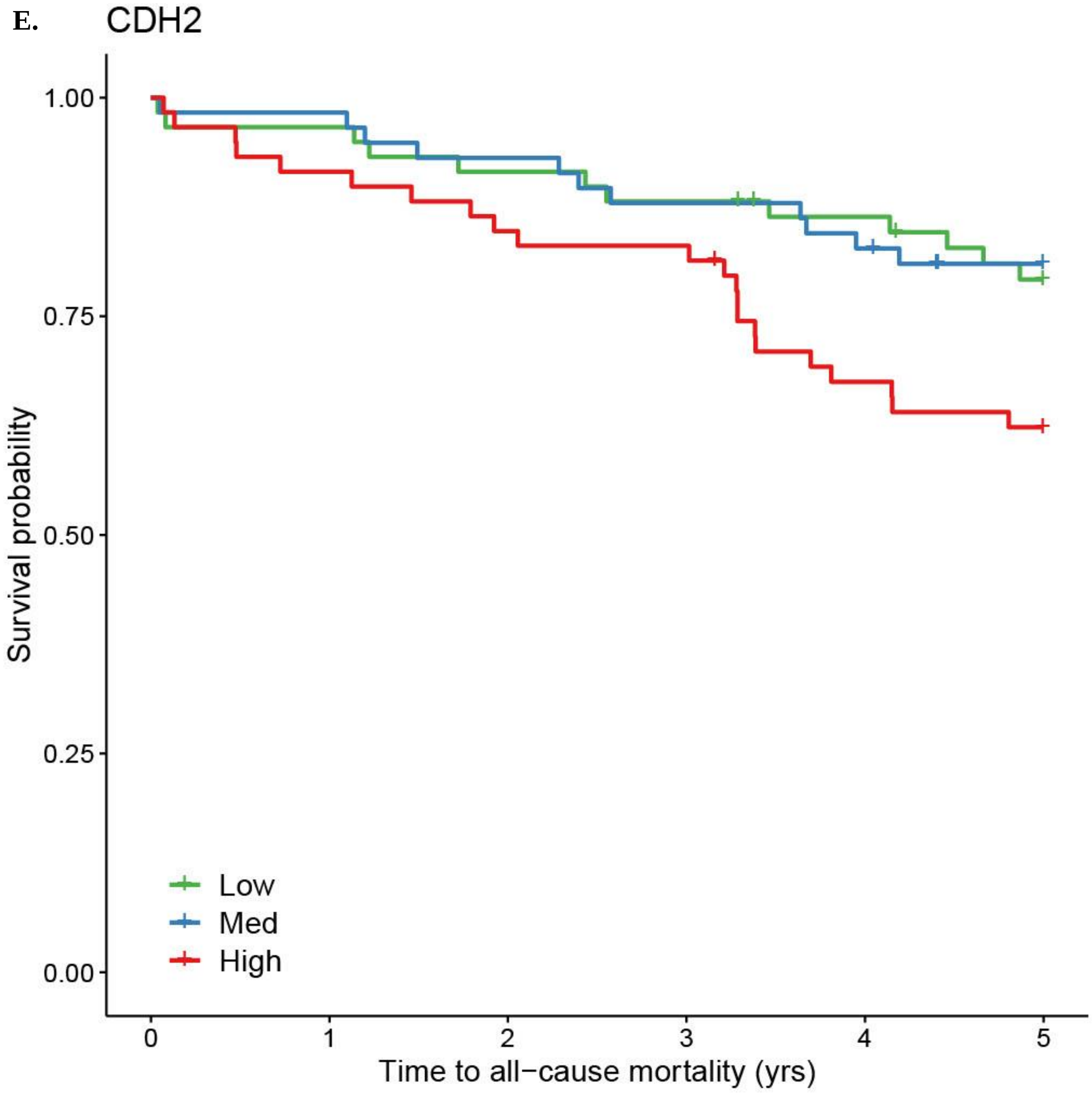
CDH2
1.00
0.75
0.50
0.25
0.00
Survival probability
0
1
2
3
4
5
Time to all−cause mortality (yrs)
Low
Med
High

F.

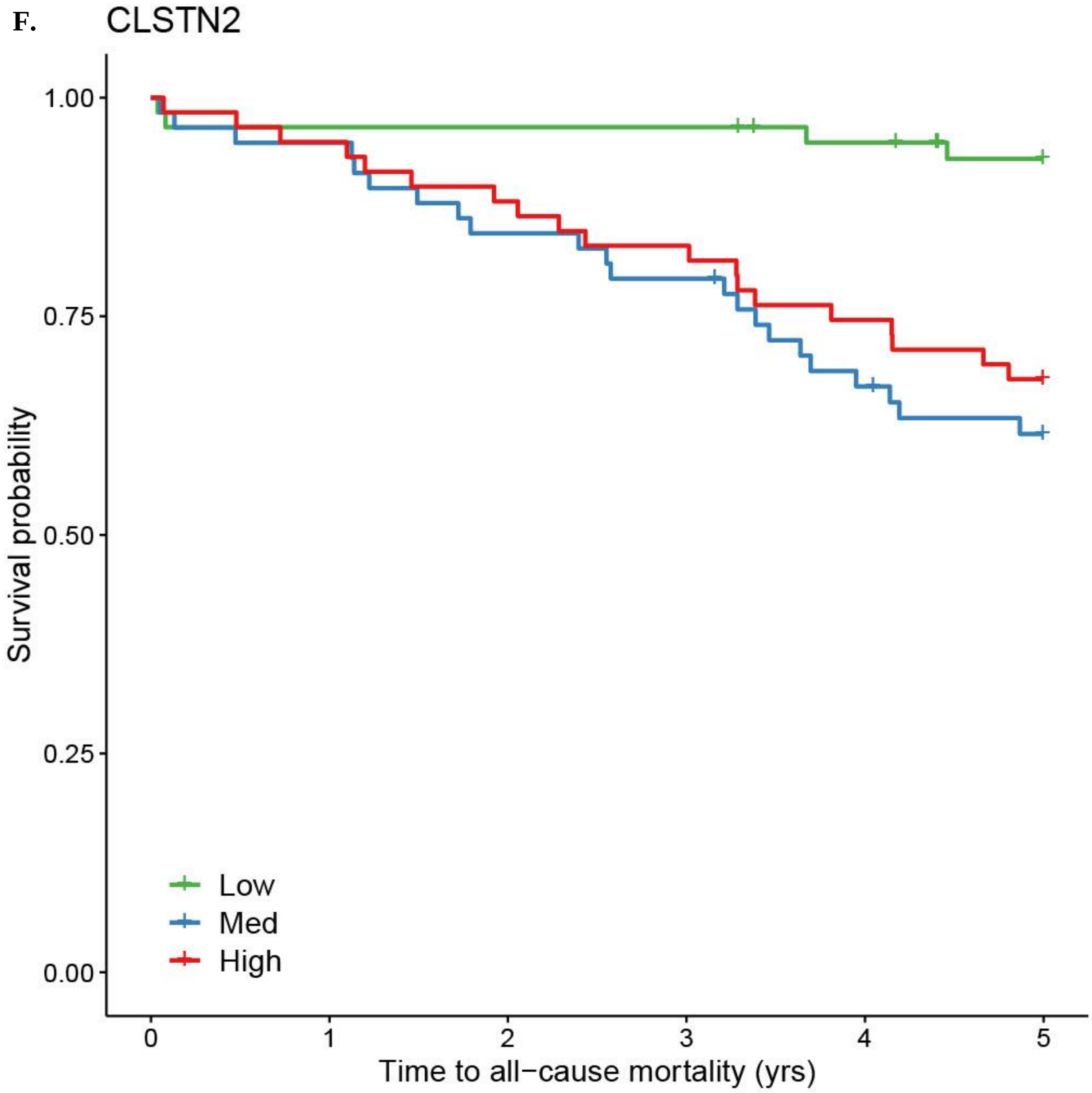

CLSTN2
1.00
0.75
0.50
0.25
0.00
Survival probability
0
1
2
3
4
5
Time to all−cause mortality (yrs)
Low
Med
High

G.

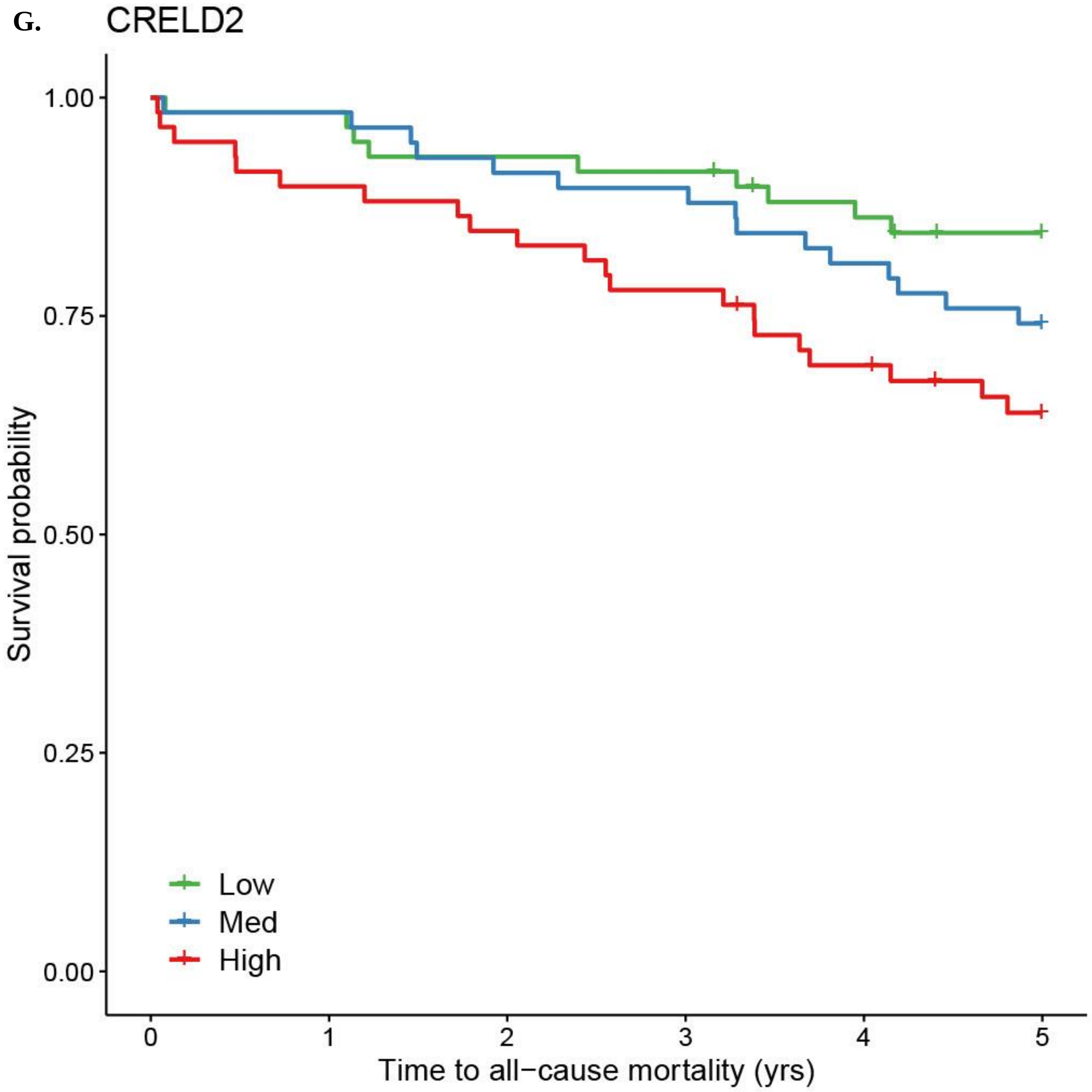
CRELD2
Survival probability
1.00
0.75
0.50
0.25
0.00
Time to all−cause mortality (yrs)
0
1
2
3
4
5
Low
Med
High

H.

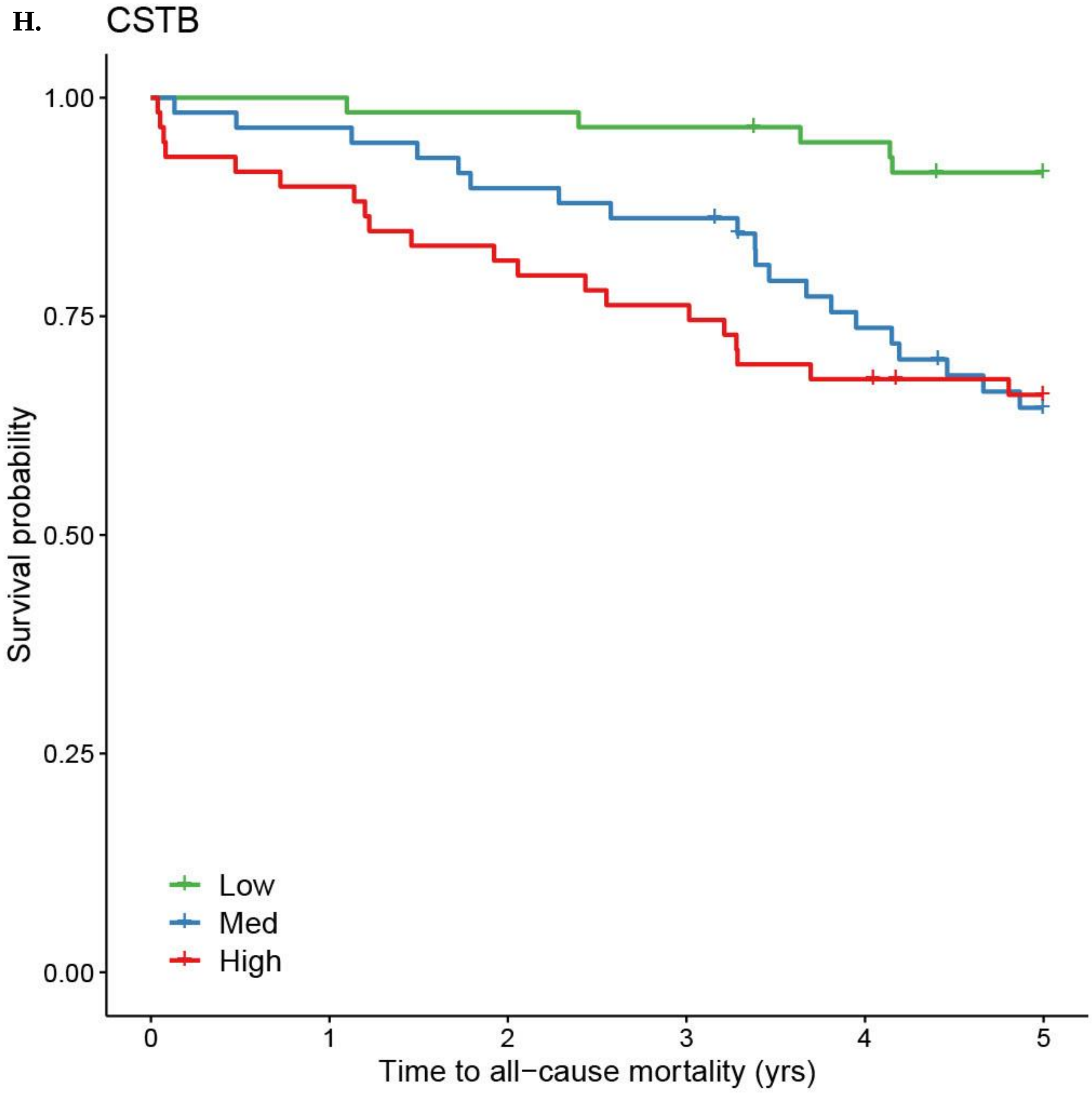
CSTB
1.00
0.75
0.50
0.25
0.00
Survival probability
Low
Med
High
0
1
2
3
4
5
Time to all−cause mortality (yrs)

I.

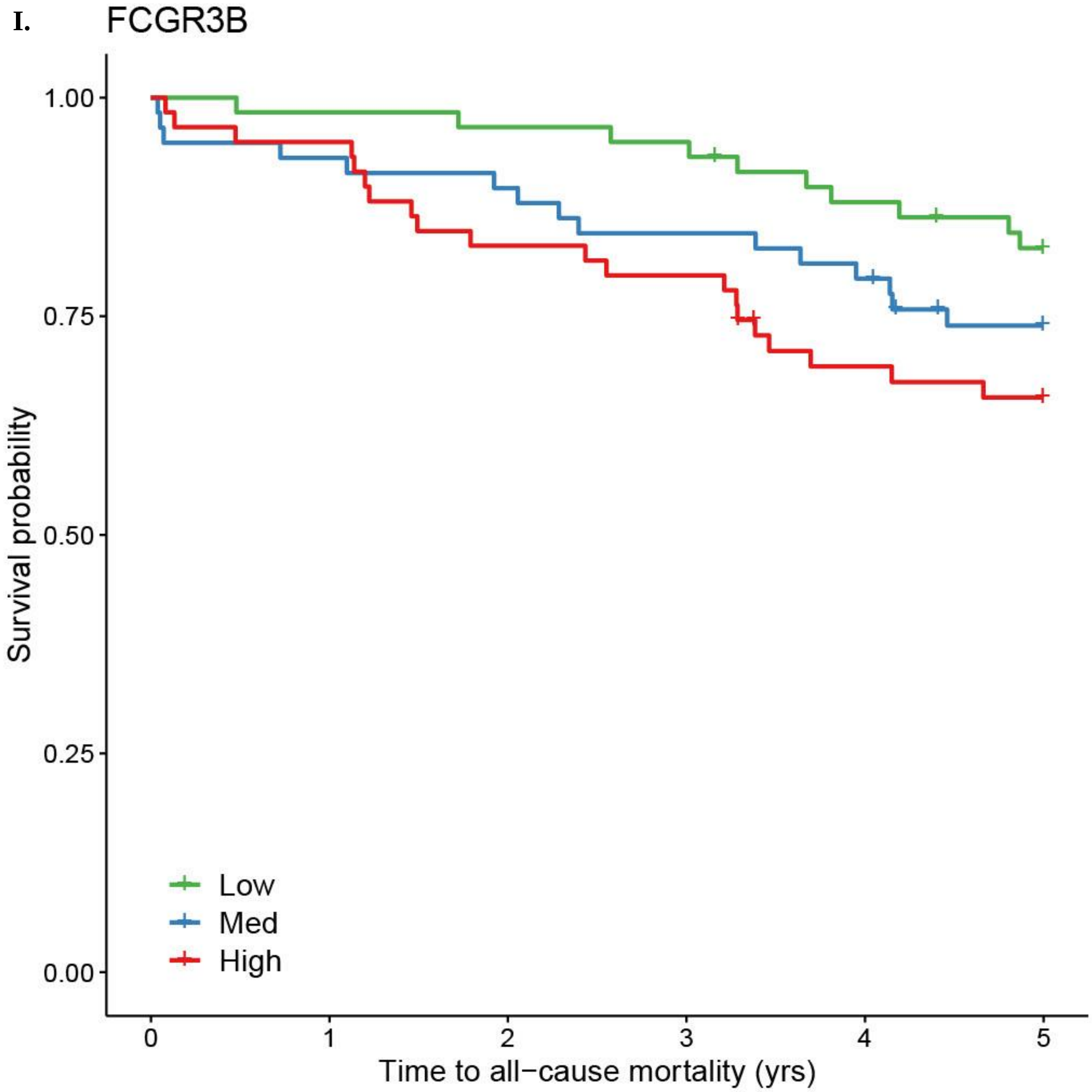
FCGR3B
Survival probability
1.00
0.75
0.50
0.25
0.00
0
1
2
3
4
5
Time to all−cause mortality (yrs)
Low
Med
High

J.

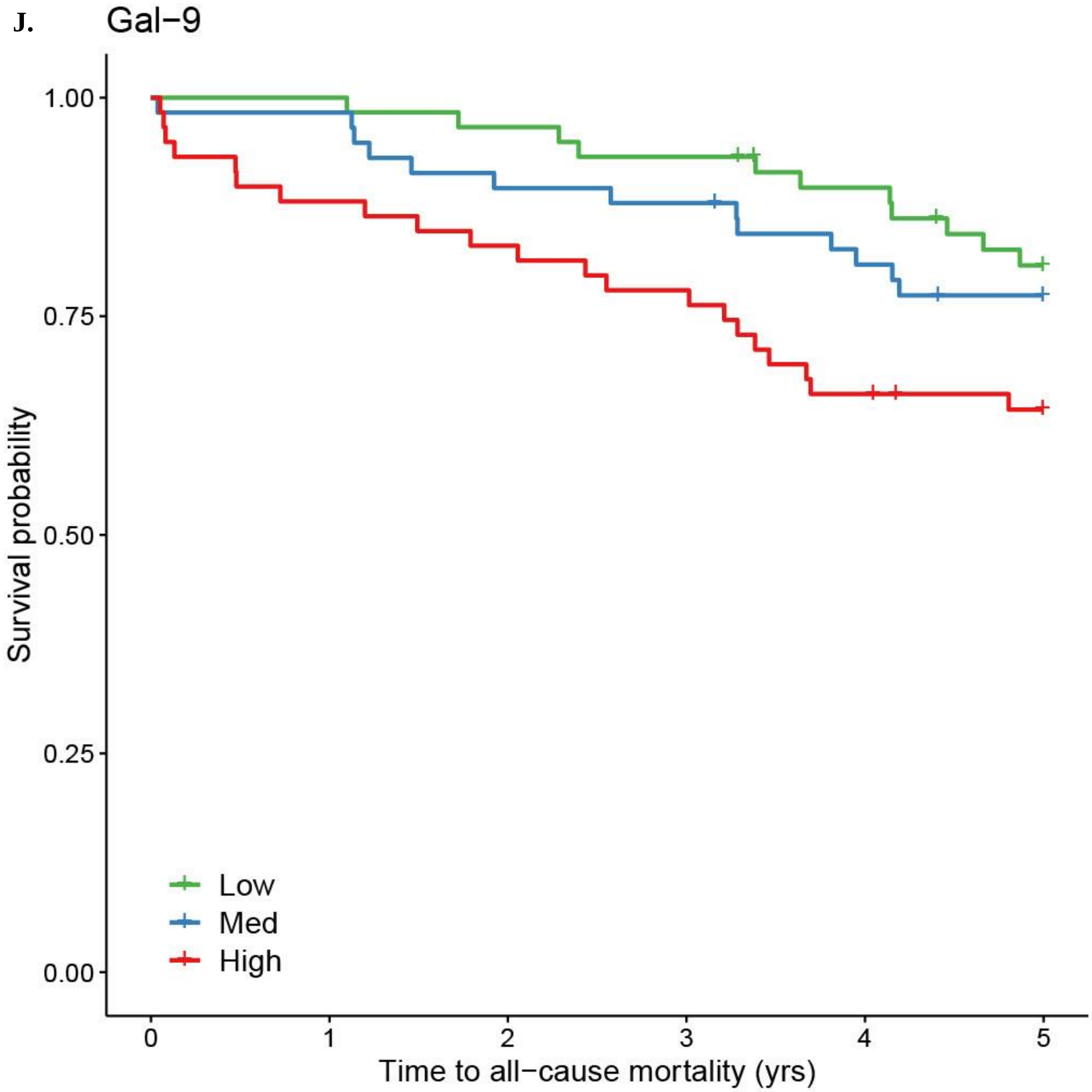

Gal−9
1.00
0.75
0.50
0.25
0.00
Survival probability
0
1
2
3
4
5
Time to all−cause mortality (yrs)
Low
Med
High

K.

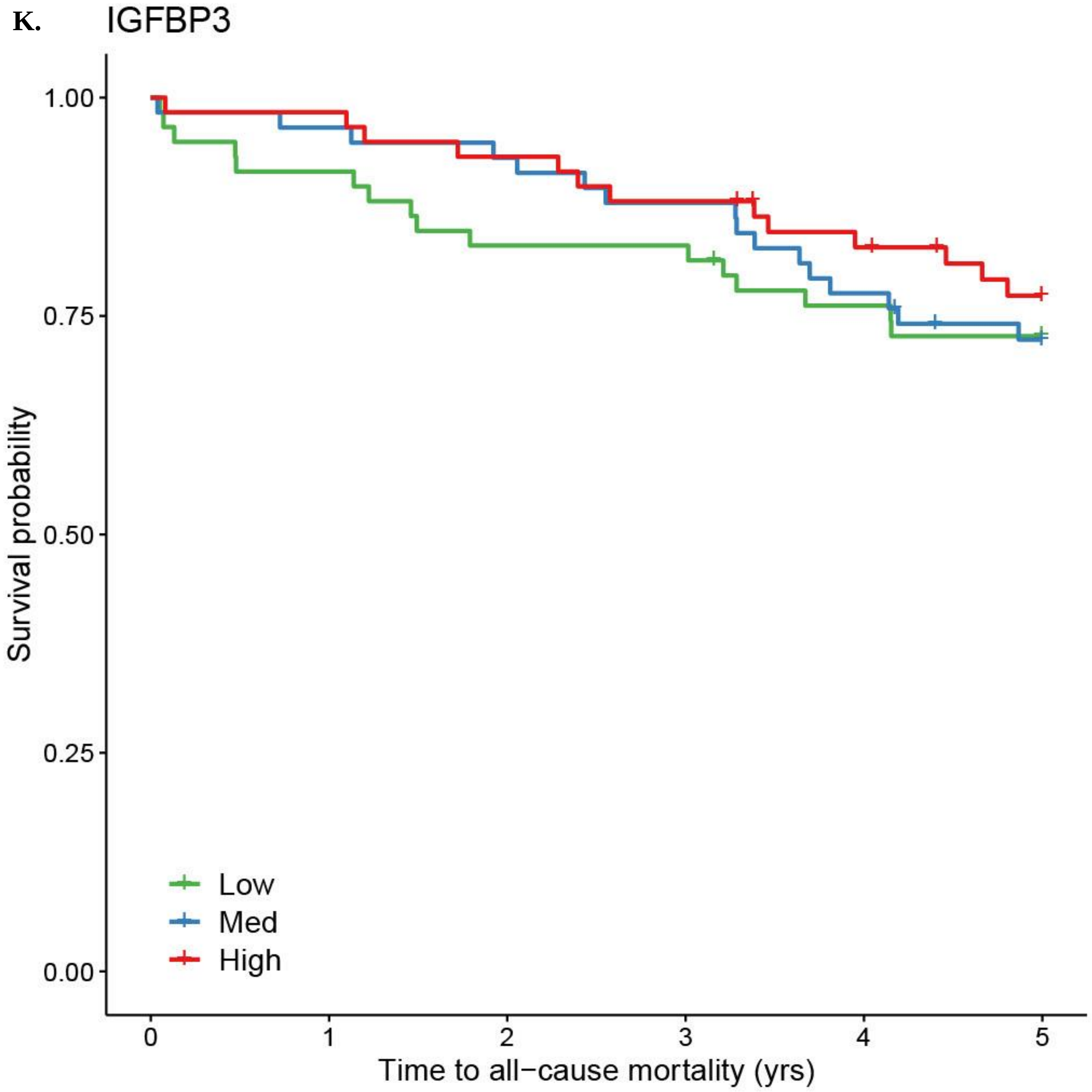

IGFBP3
1.00
0.75
0.50
0.25
0.00
Survival probability
0
1
2
3
4
5
Time to all−cause mortality (yrs)
Low
Med
High

L.

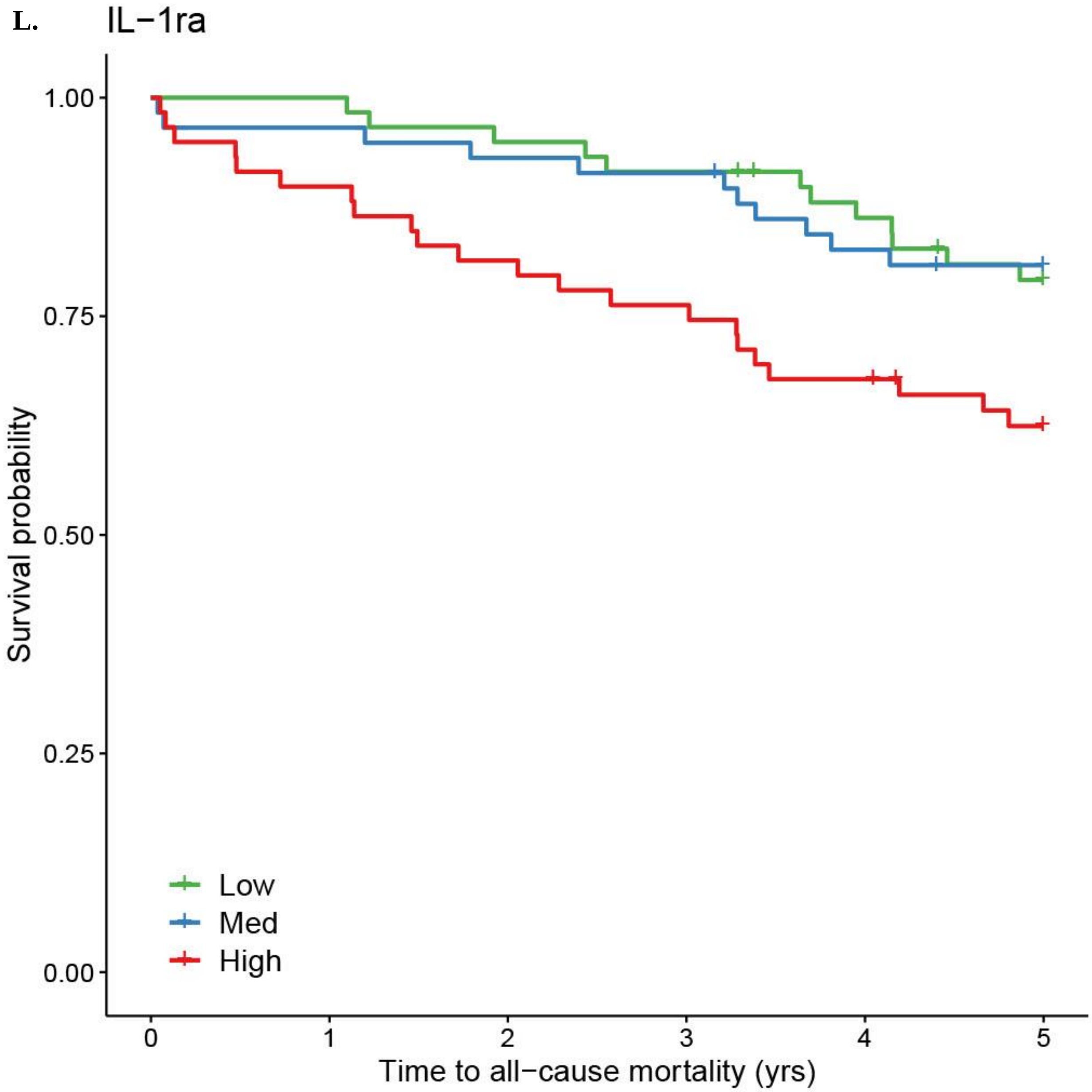
IL−1ra
Survival probability
1.00
0.75
0.50
0.25
0.00
0
1
2
3
4
5
Time to all−cause mortality (yrs)
Low
Med
High

M.

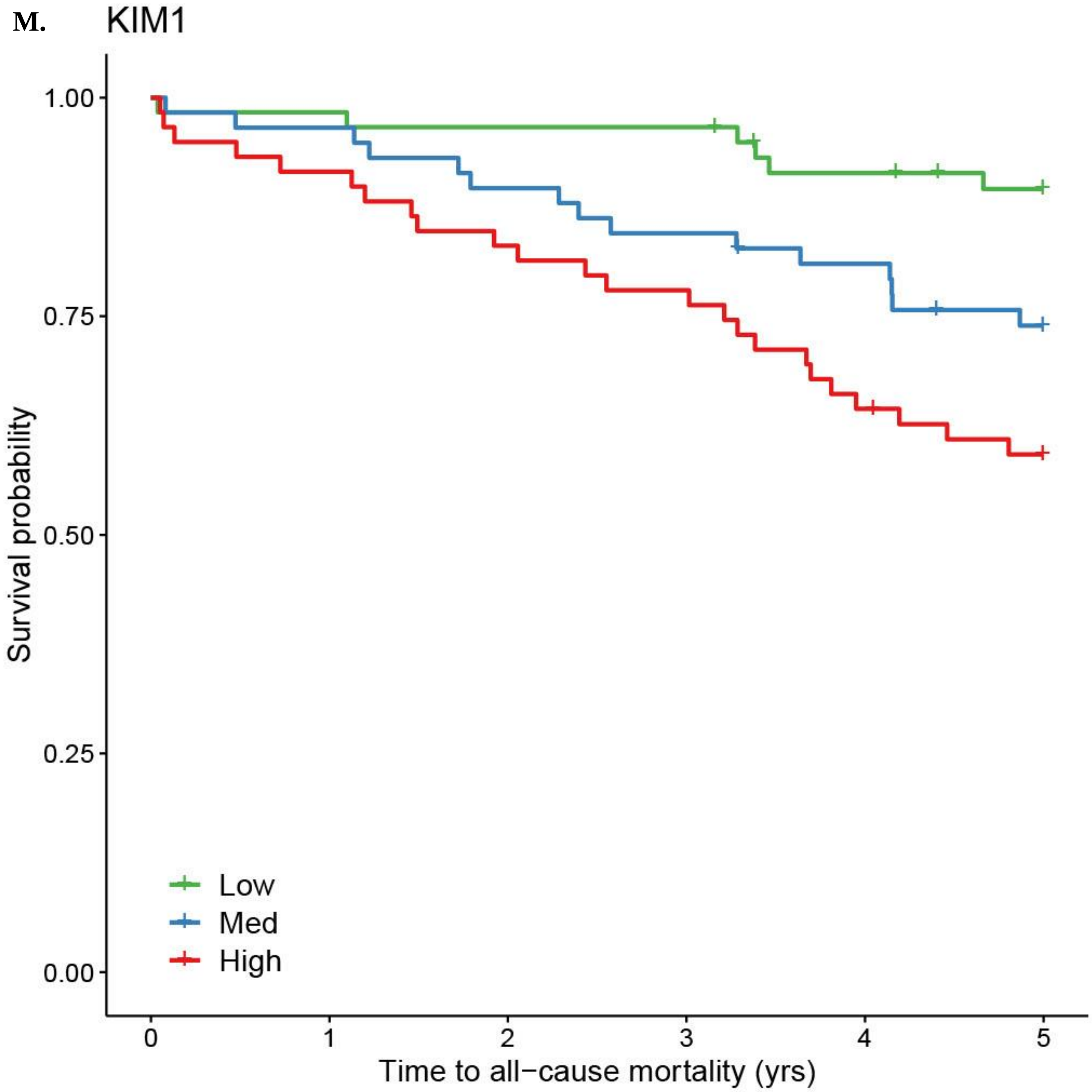
KIM1
1.00
0.75
0.50
0.25
0.00
Survival probability
Low
Med
High
0
1
2
3
4
5
Time to all−cause mortality (yrs)

N.

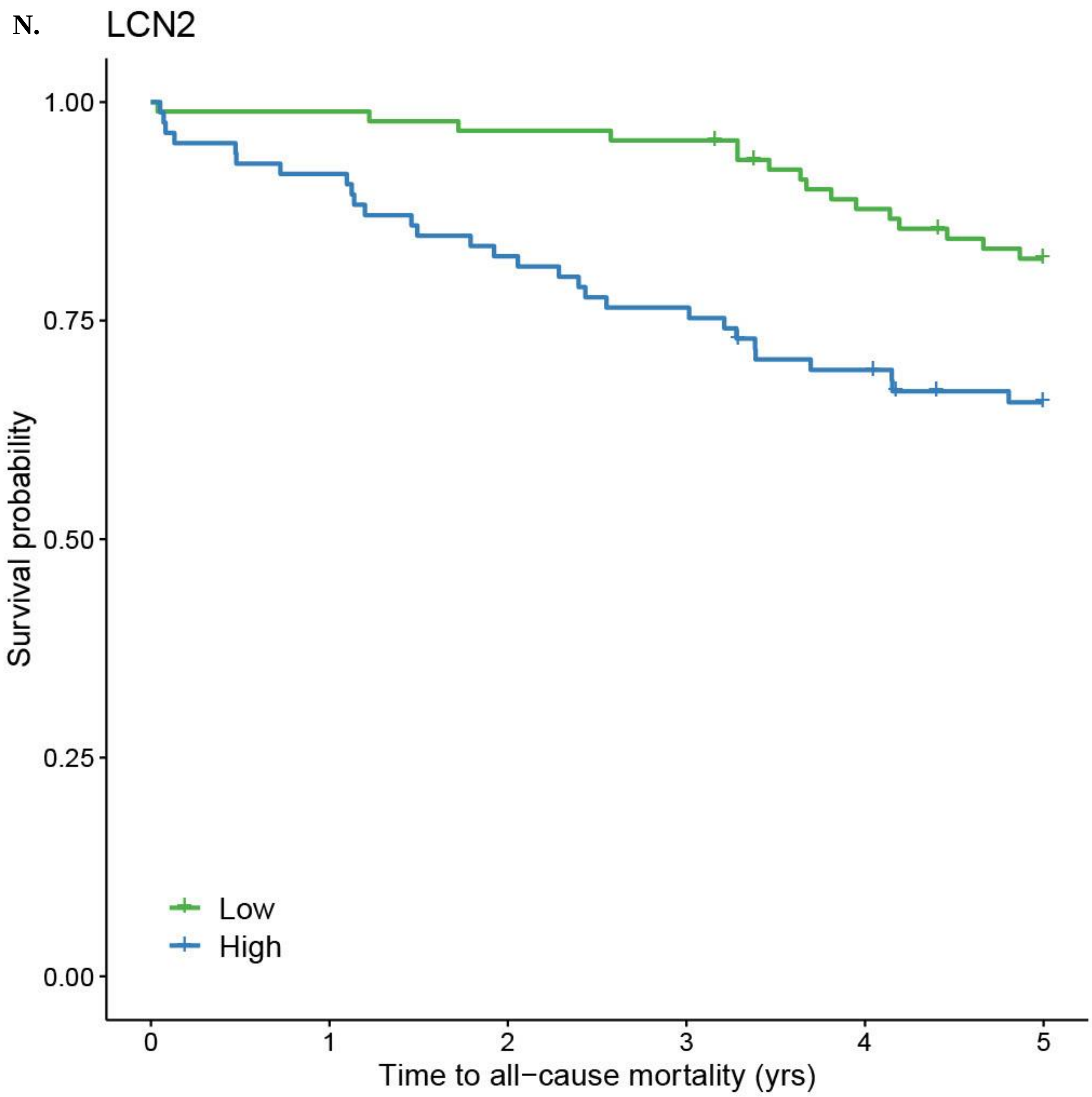
LCN2
1.00
0.75
0.50
0.25
0.00
Survival probability
0
1
2
3
4
5
Time to all−cause mortality (yrs)
Low
High

O. MATN2

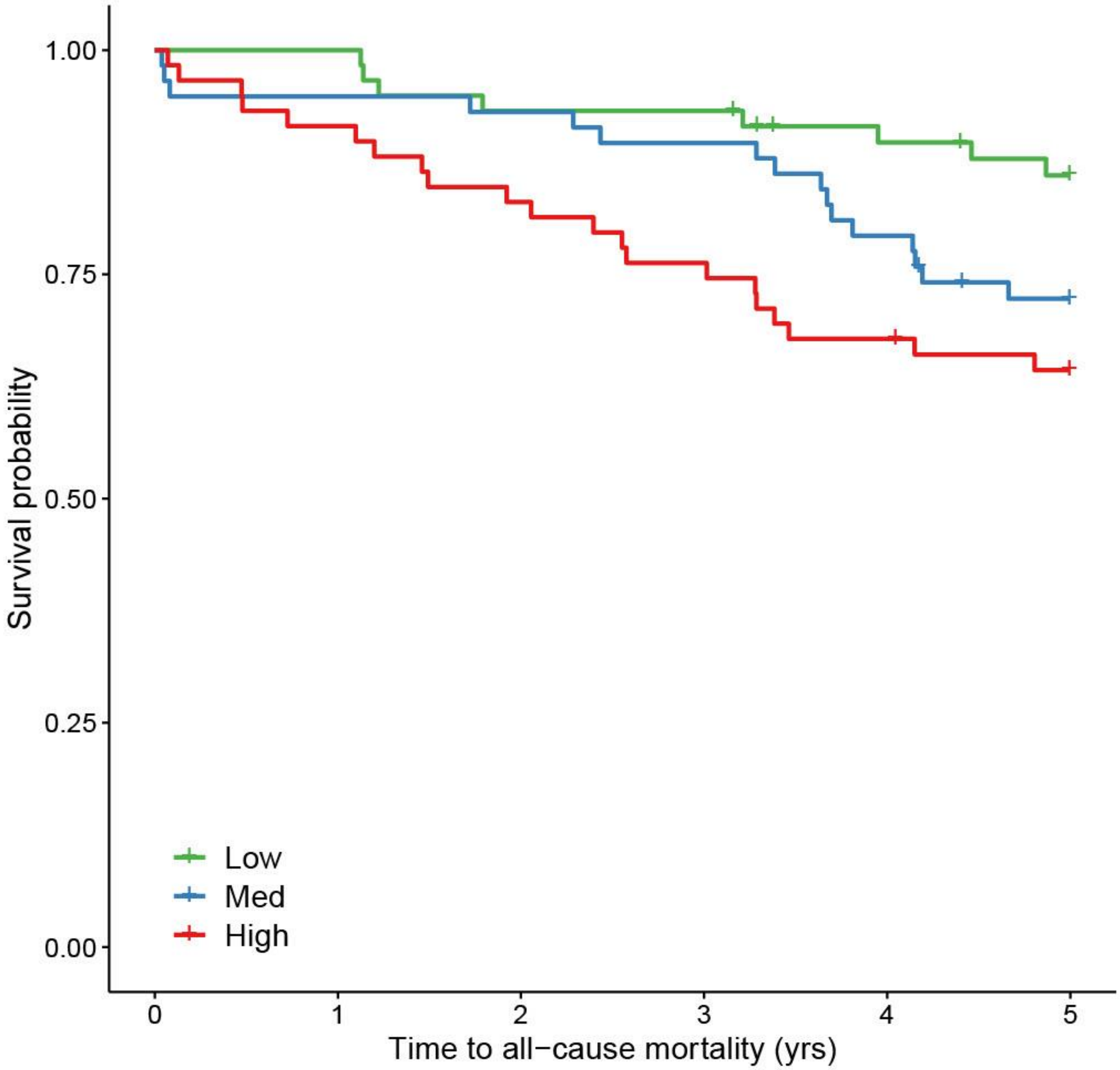
1.00
0.75
0.50
0.25
0.00
Survival probability
0
1
2
3
4
5
Time to all−cause mortality (yrs)
Low
Med
High

P.

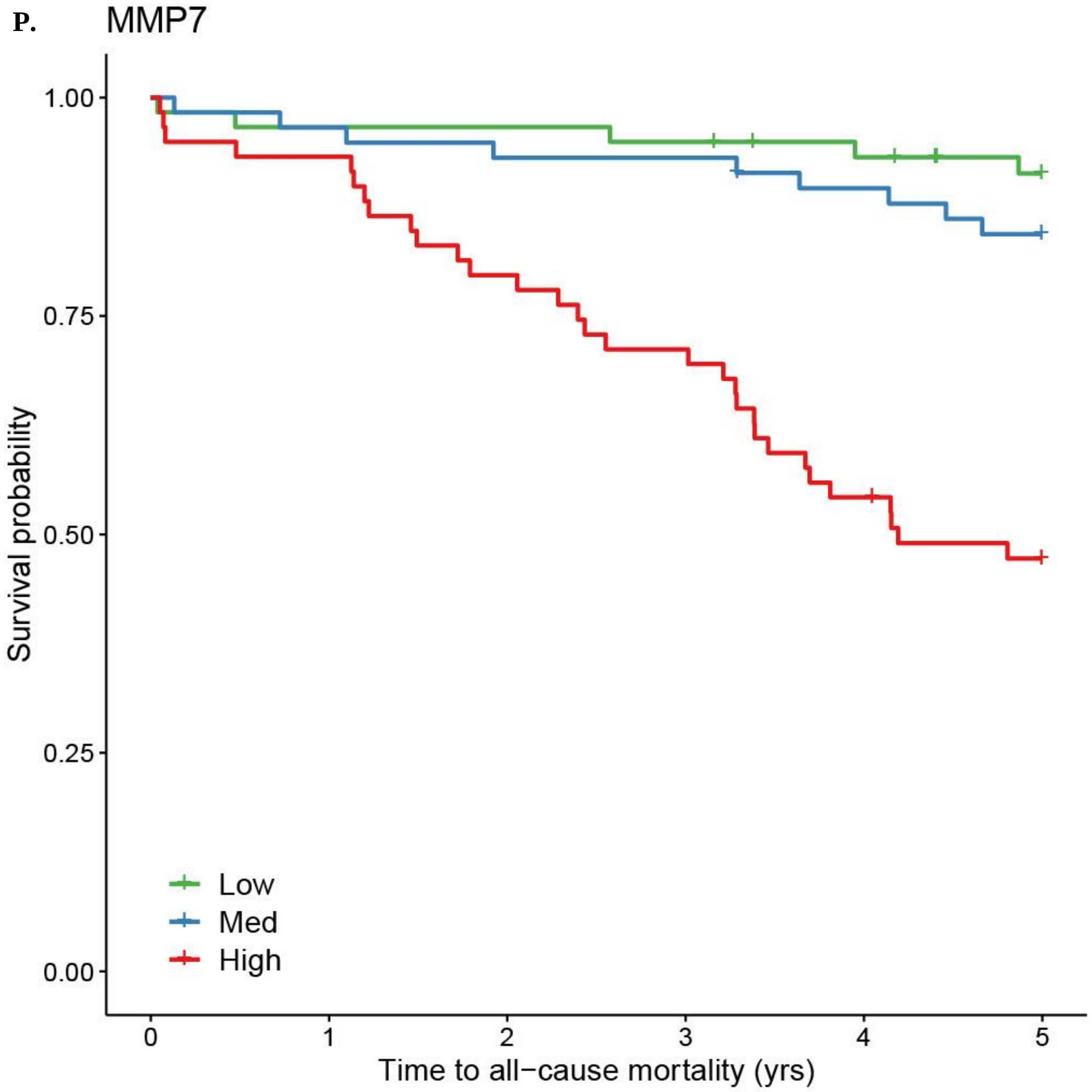
MMP7
Survival probability
1.00
0.75
0.50
0.25
0.00
0
1
2
3
4
5
Time to all−cause mortality (yrs)
Low
Med
High

Q.

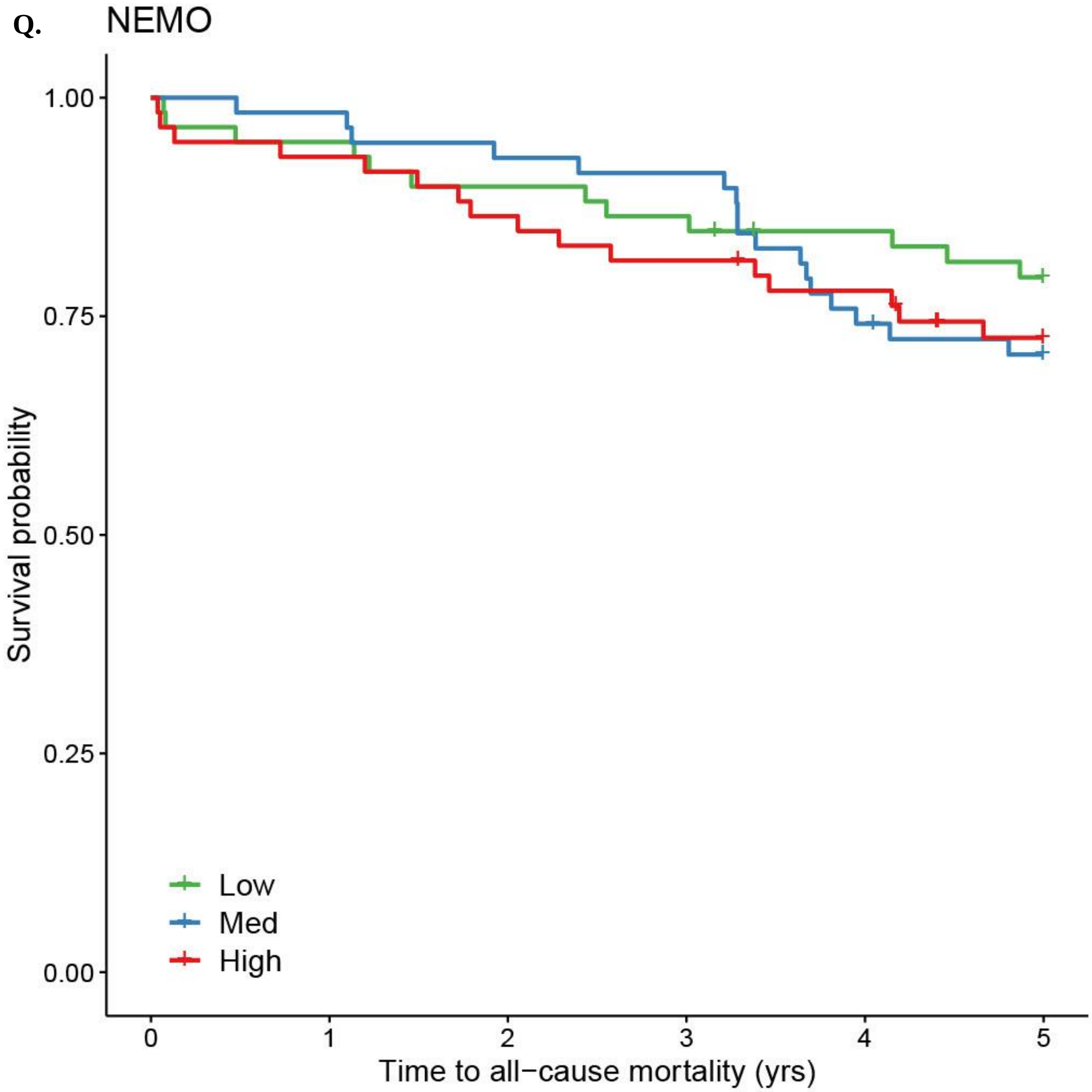
NEMO
1.00
0.75
0.50
0.25
0.00
Survival probability
0
1
2
3
4
5
Time to all−cause mortality (yrs)
Low
Med
High

R.

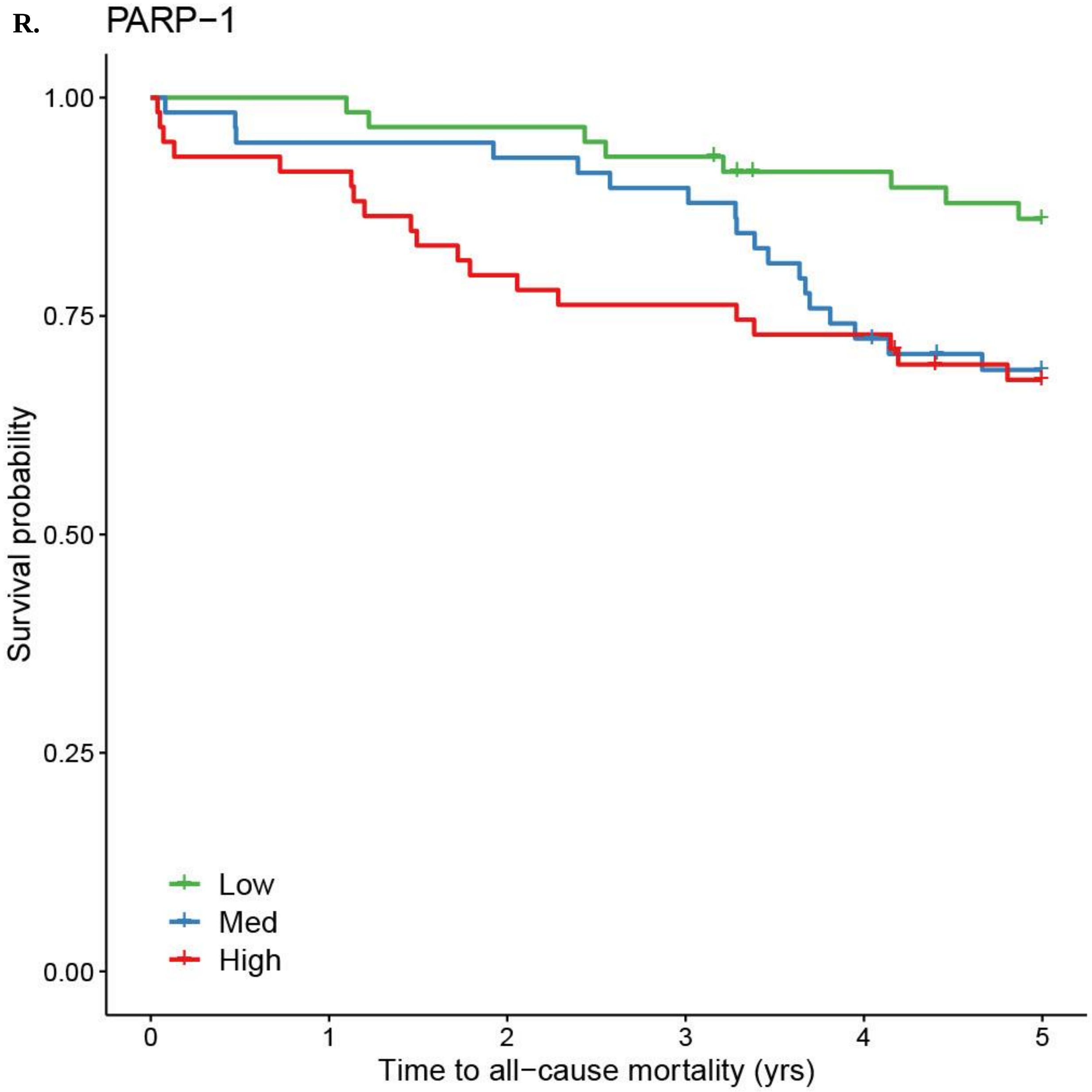
PARP−1
1.00
0.75
0.50
0.25
0.00
Survival probability
Low
Med
High
0
1
2
3
4
5
Time to all−cause mortality (yrs)

s.

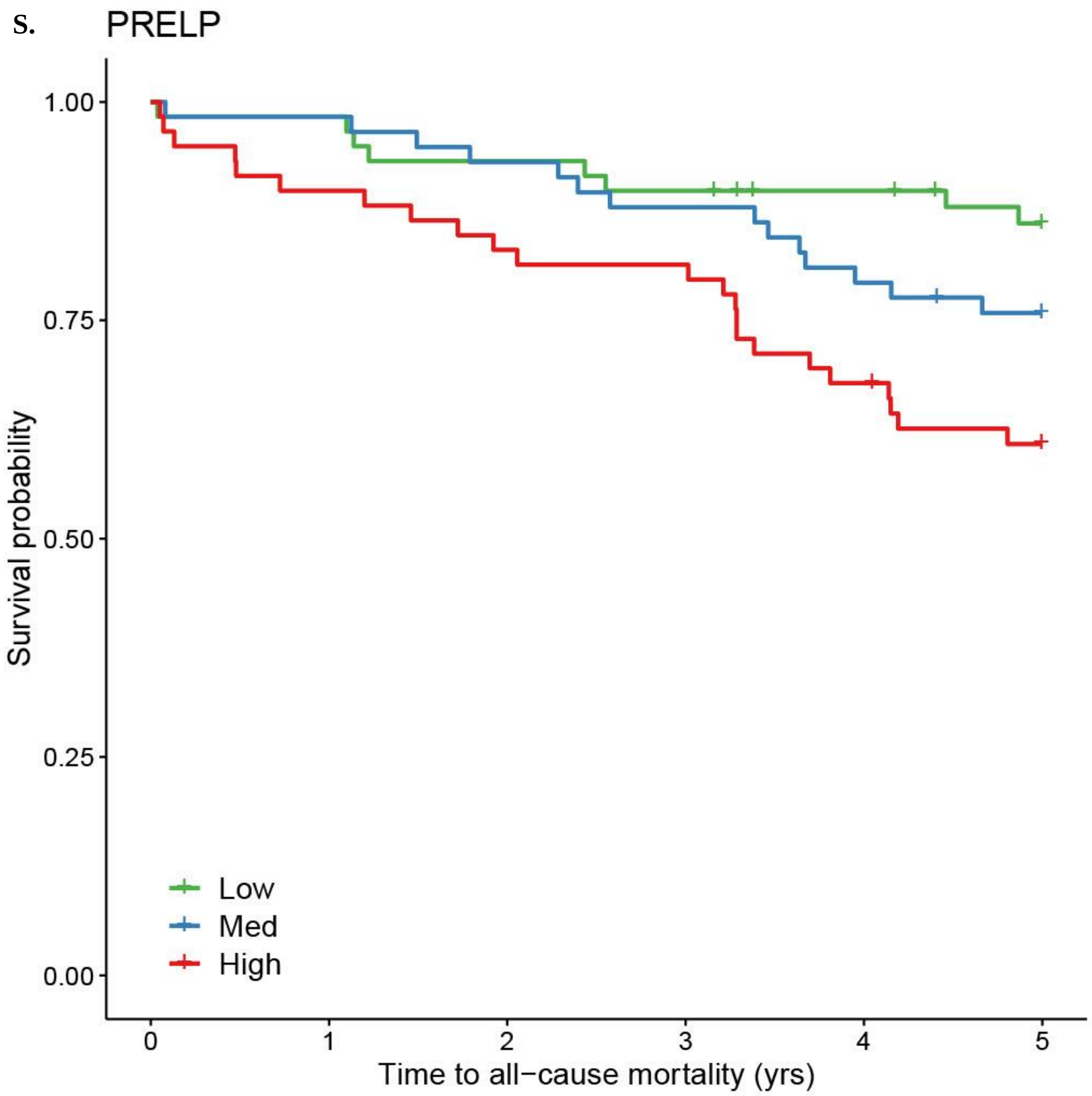
PRELP
1.00
0.75
0.50
0.25
0.00
Survival probability
0
1
2
3
4
5
Time to all−cause mortality (yrs)
Low
Med
High

T.

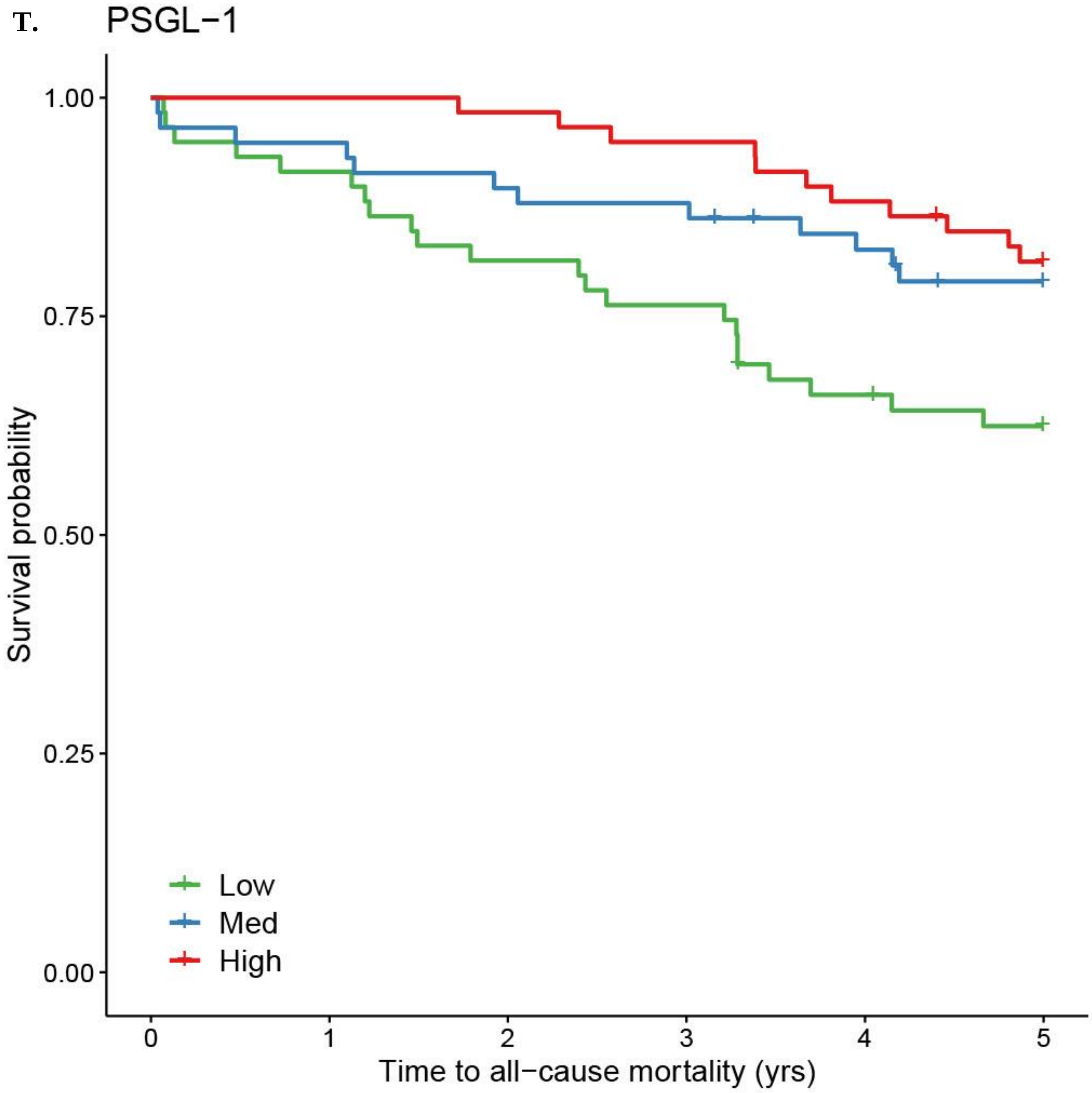
PSGL−1
1.00
0.75
0.50
0.25
0.00
Survival probability
0
1
2
3
4
5
Time to all−cause mortality (yrs)
Low
Med
High

U.

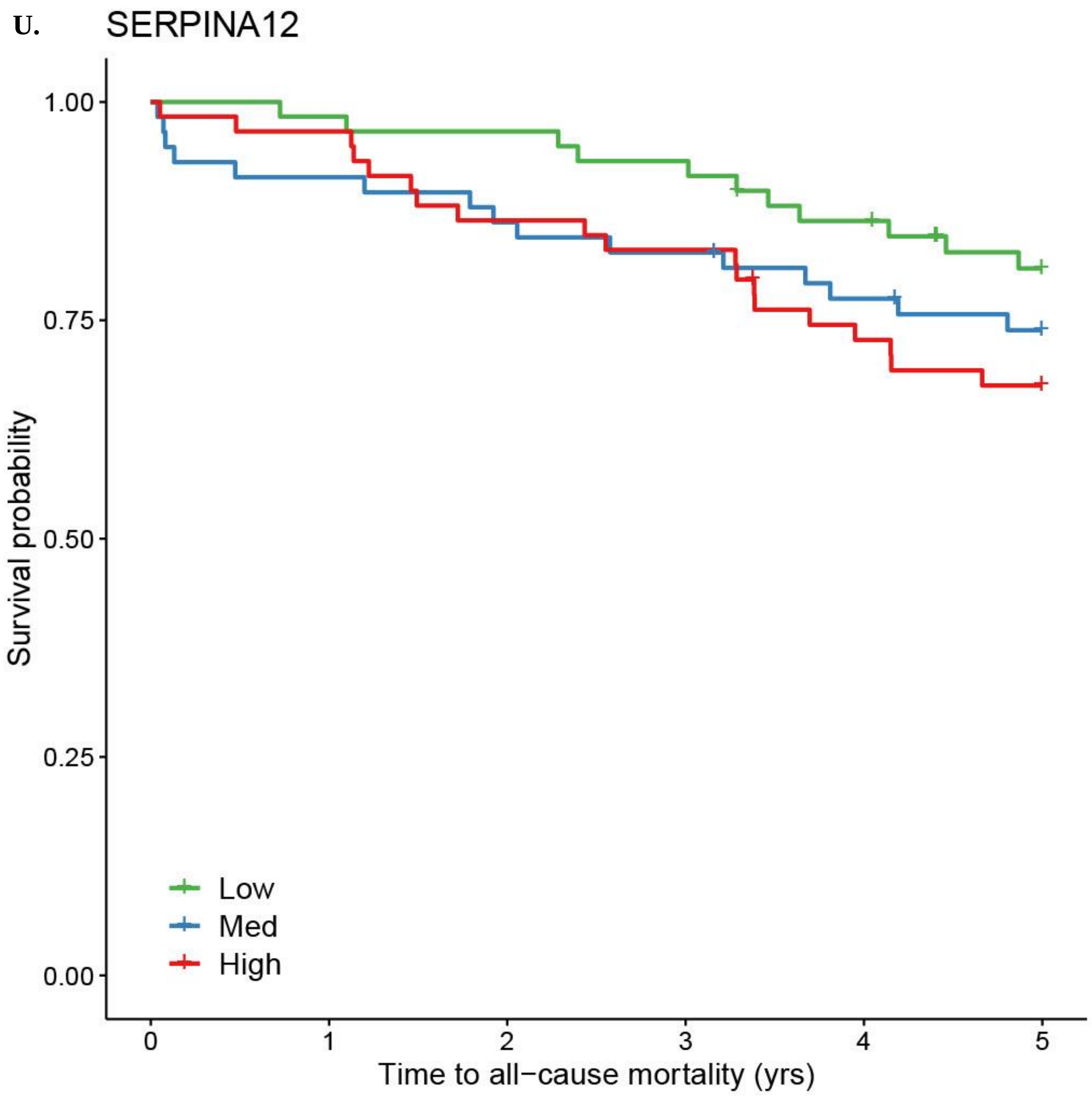
SERPINA12
Survival probability
1.00
0.75
0.50
0.25
0.00
Time to all−cause mortality (yrs)
0
1
2
3
4
5
Low
Med
High

v. SPINT2

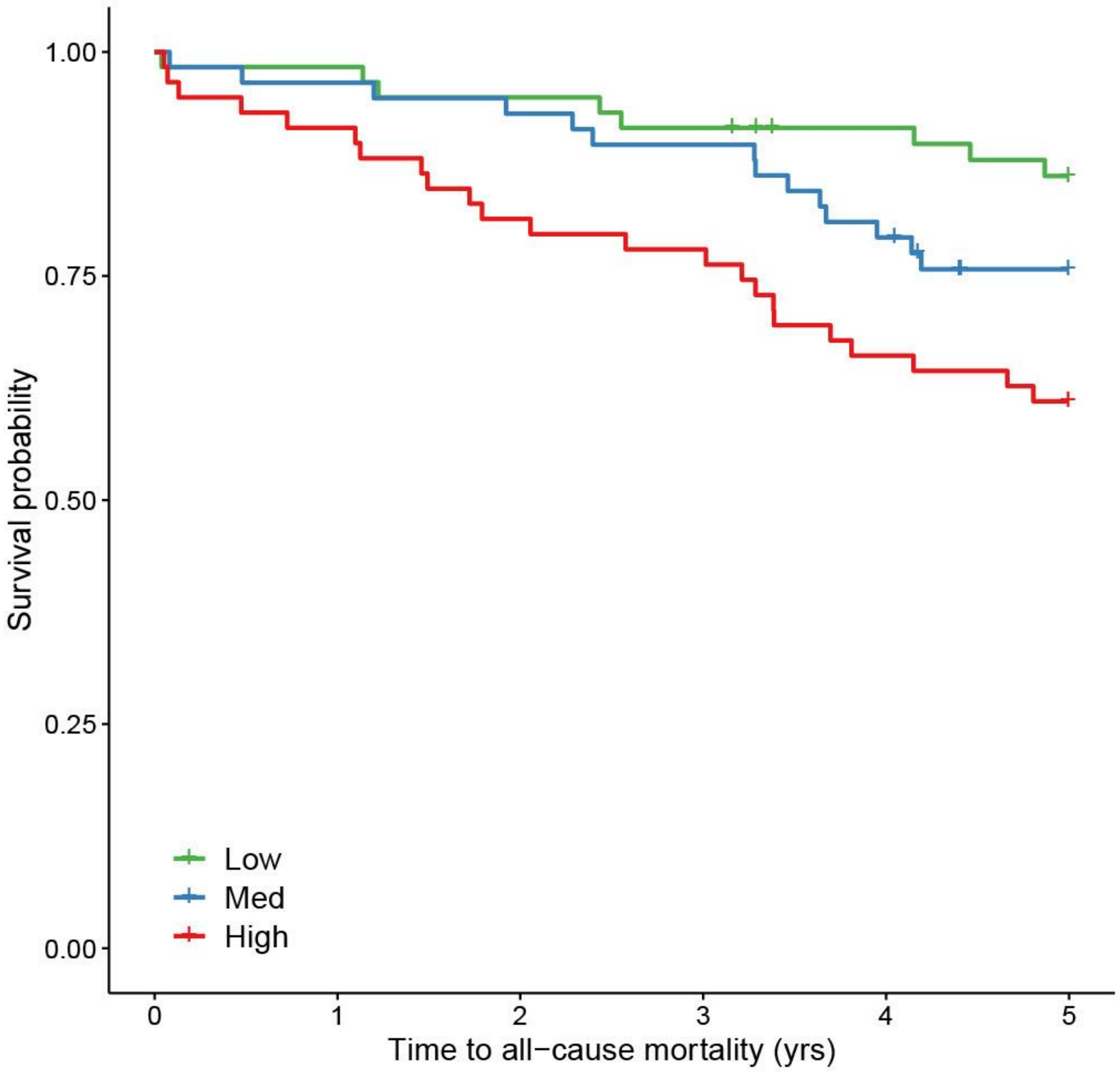
1.00
0.75
0.50
0.25
0.00
Survival probability
0
1
2
3
4
5
Time to all−cause mortality (yrs)
Low
Med
High

w.

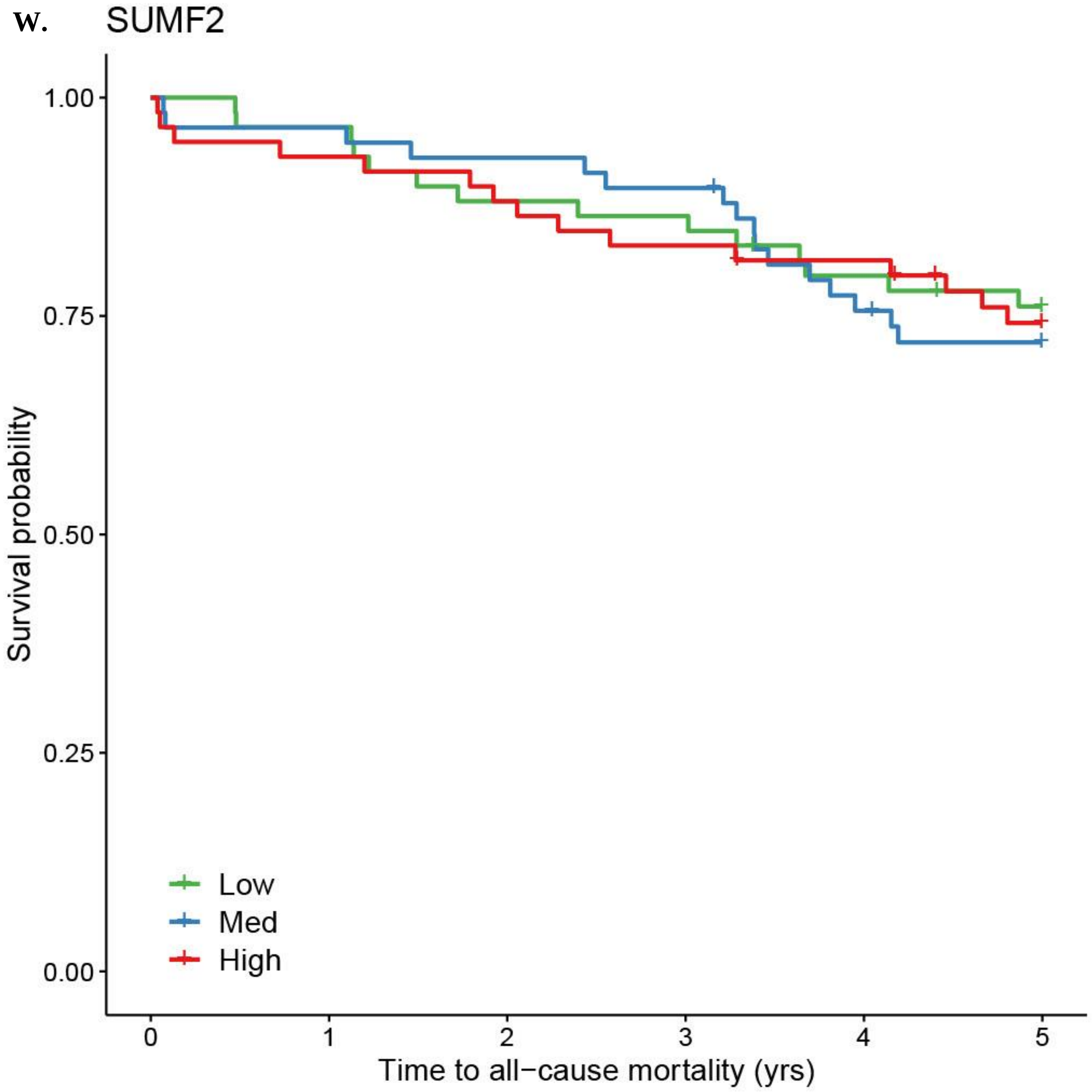
SUMF2
1.00
0.75
0.50
0.25
0.00
Survival probability
0
1
2
3
4
5
Time to all−cause mortality (yrs)
Low
Med
High

x. TIMP1

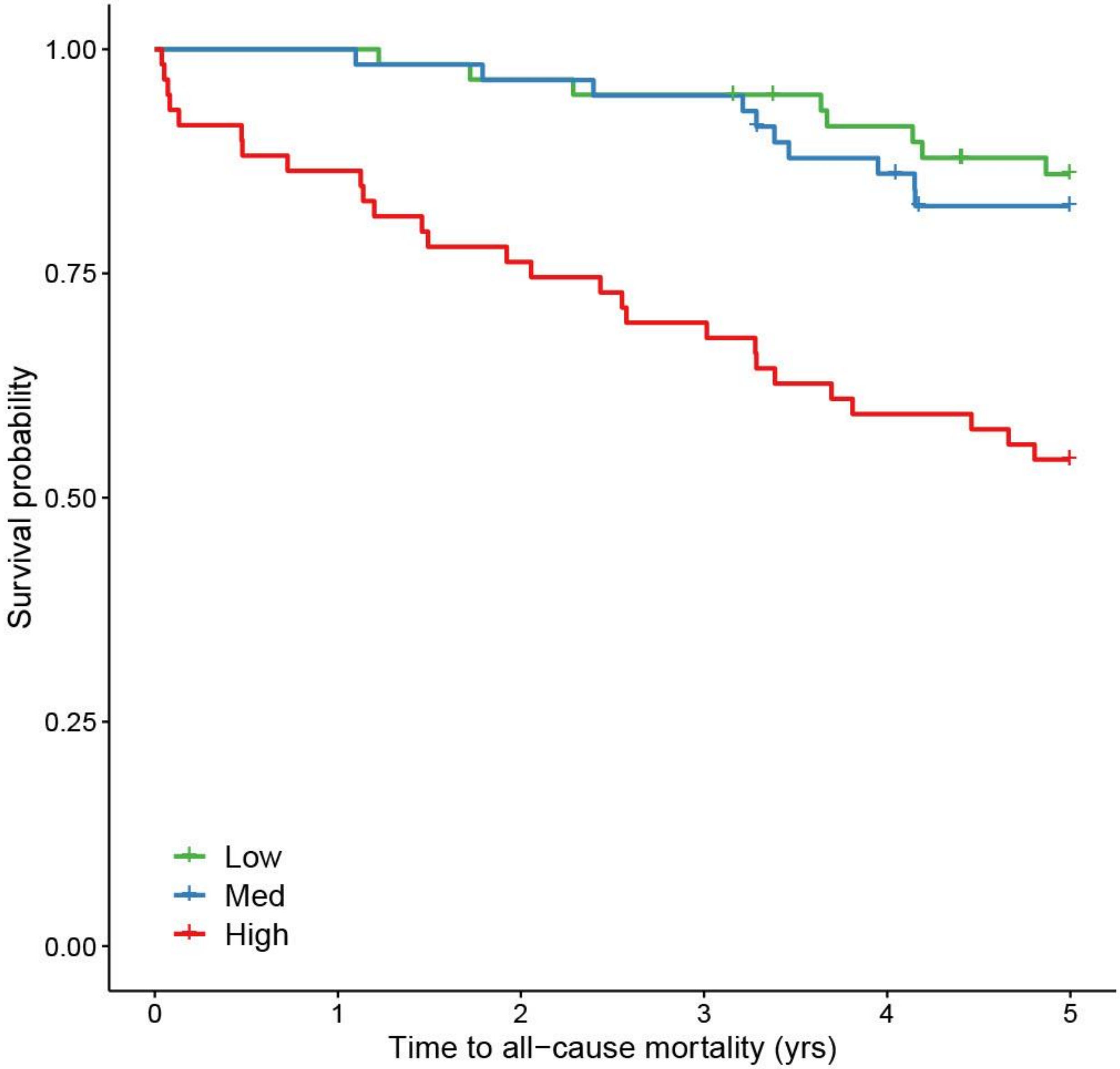

Survival probability
1.00
0.75
0.50
0.25
0.00
Low
Med
High
0
1
2
3
4
5
Time to all−cause mortality (yrs)

Y.

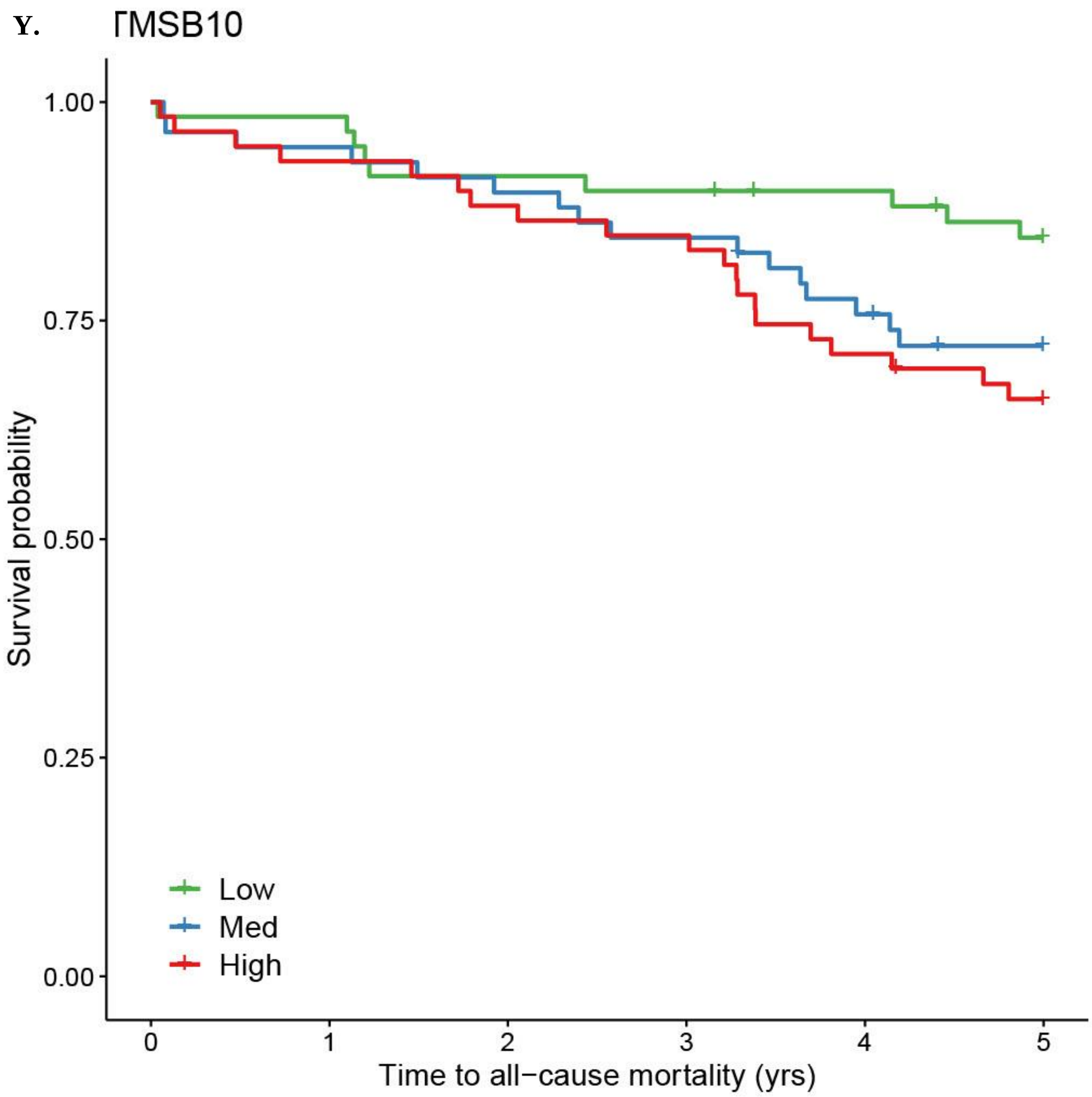
TMSB10
1.00
0.75
0.50
0.25
0.00
Survival probability
0
1
2
3
4
5
Time to all−cause mortality (yrs)
Low
Med
High

Z.

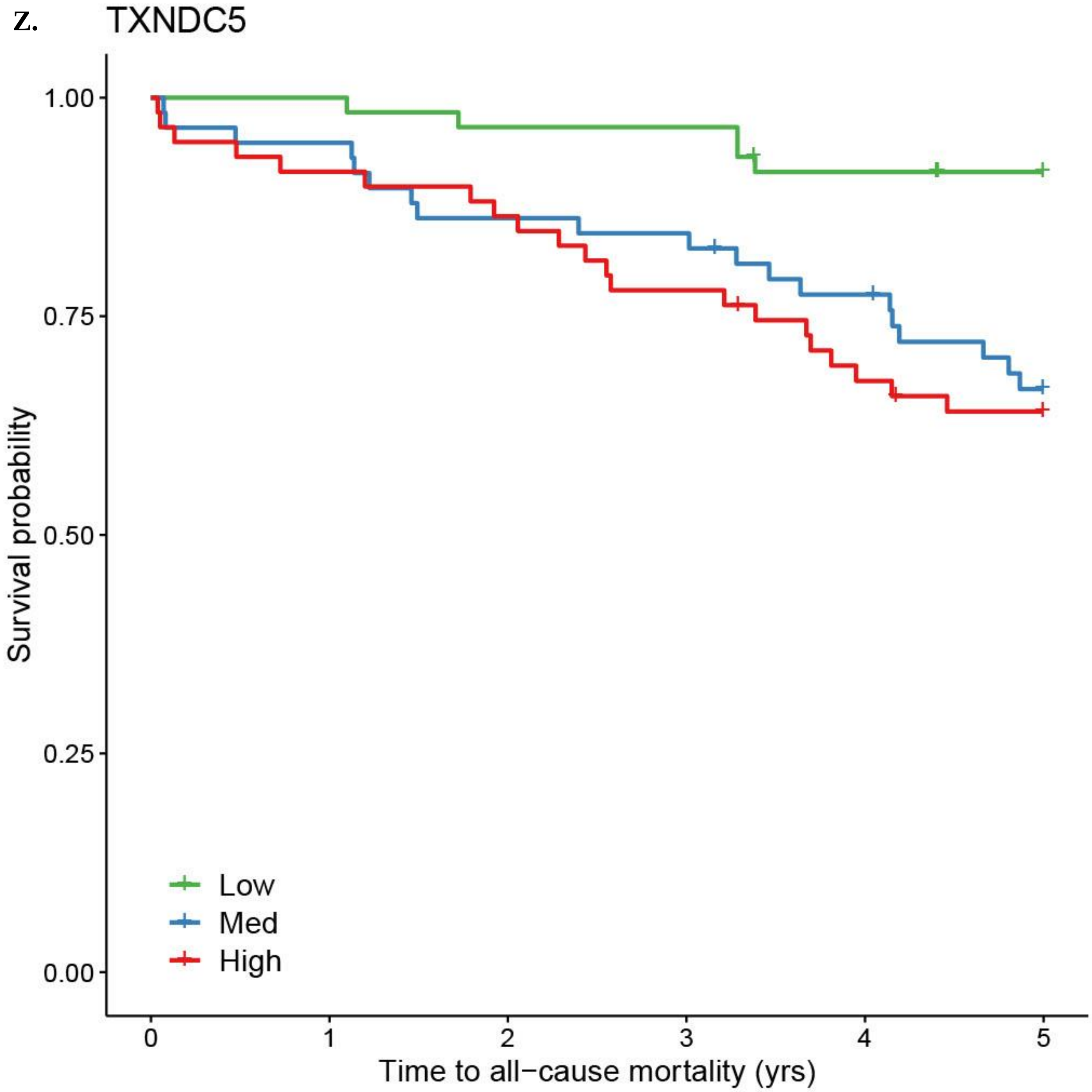
TXNDC5
Survival probability
1.00
0.75
0.50
0.25
0.00
0
1
2
3
4
5
Time to all−cause mortality (yrs)
Low
Med
High

AA.

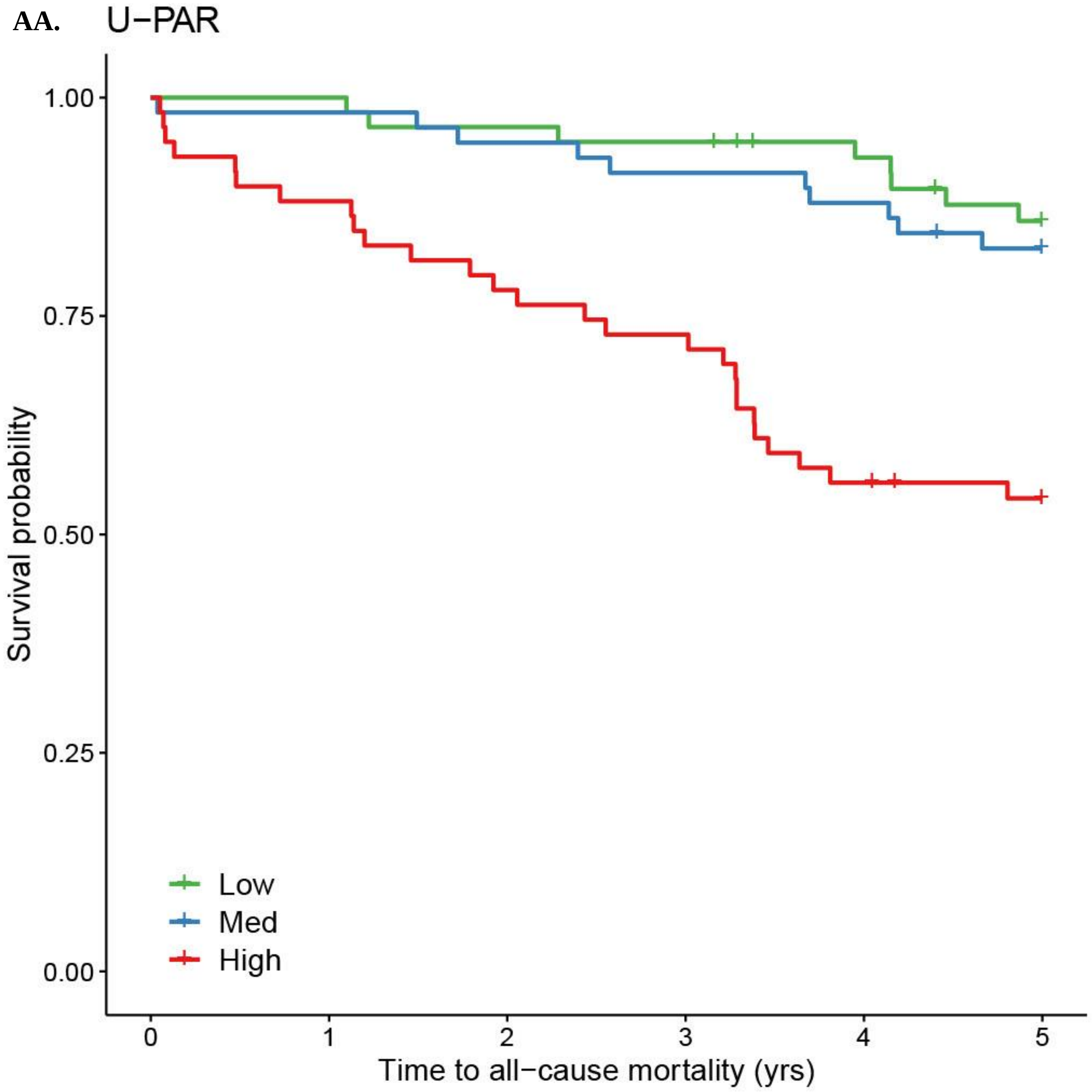
U−PAR
1.00
0.75
0.50
0.25
0.00
Survival probability
0
1
2
3
4
5
Time to all−cause mortality (yrs)
Low
Med
High

AB.

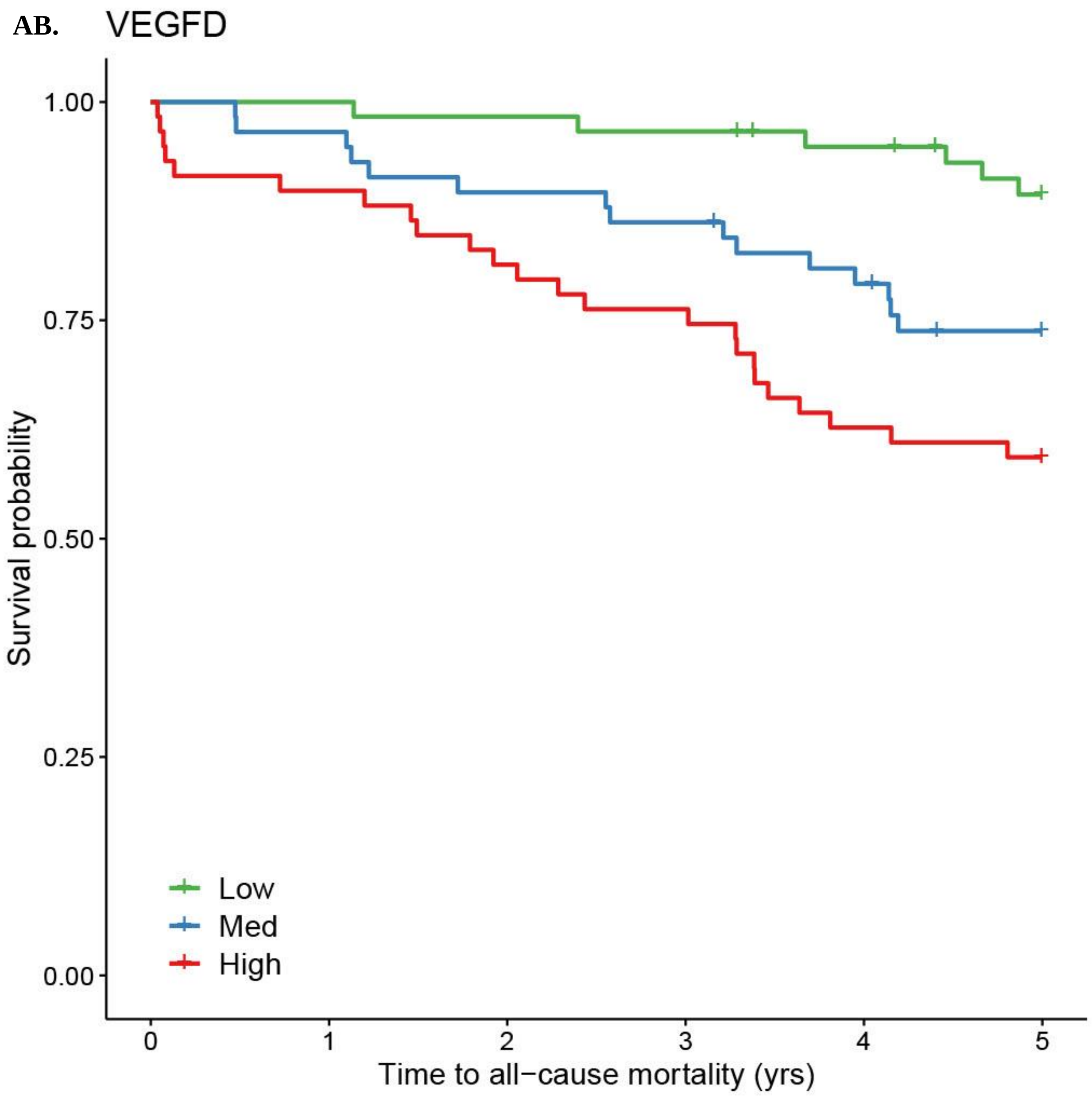
VEGFD
1.00
0.75
0.50
0.25
0.00
Survival probability
Low
Med
High
0
1
2
3
4
5
Time to all−cause mortality (yrs)

AC.

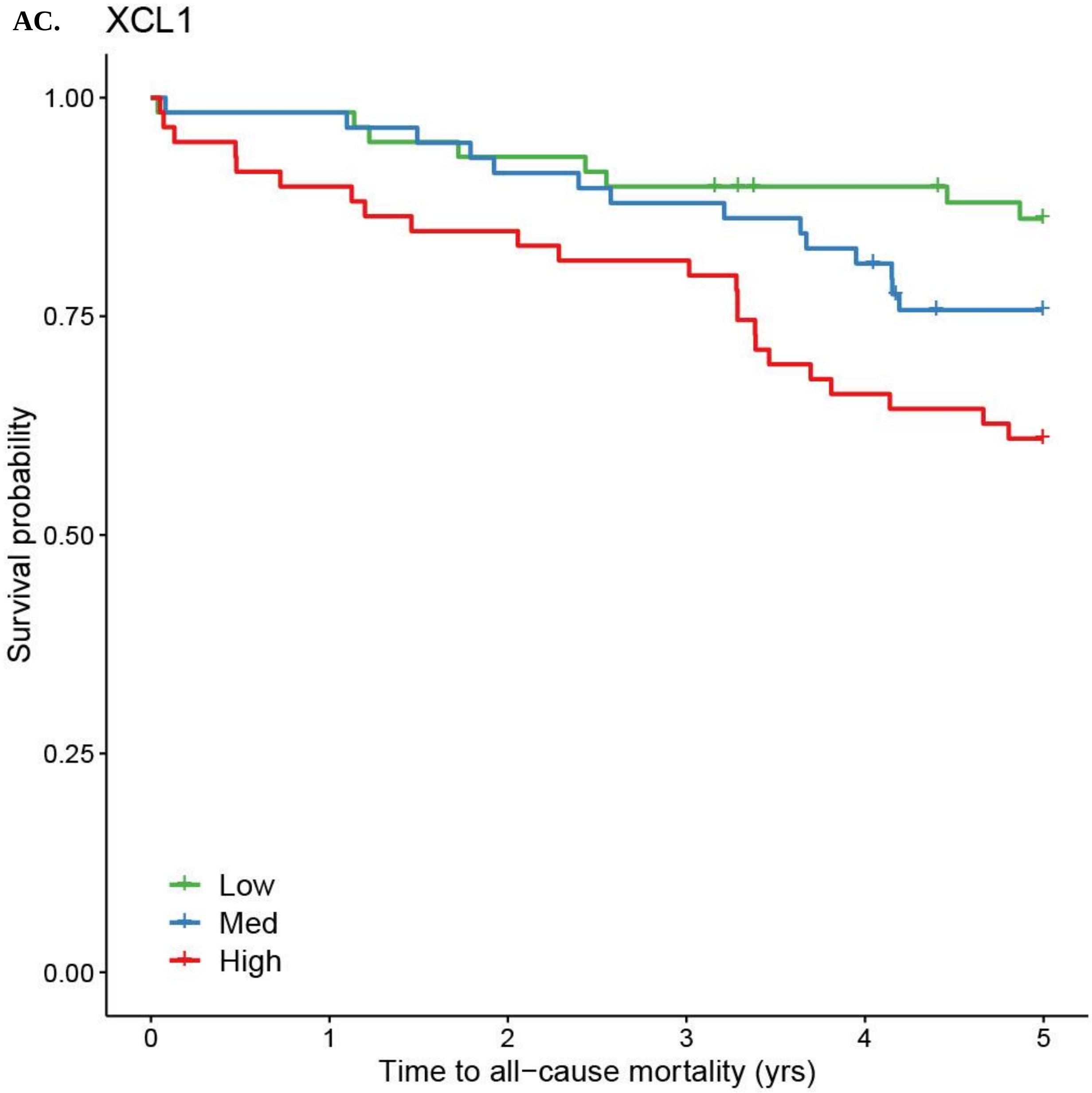
XCL1
Survival probability
1.00
0.75
0.50
0.25
0.00
0
1
2
3
4
5
Time to all−cause mortality (yrs)
Low
Med
High

Supplemental Table 1: Heart failure diagnosis codes queried for incident hospitalization for heart failure in CATHGEN
Supplemental Table 2: Comparison of cohorts included in analysis
Supplemental Table 3: Full results of 459 proteins tested in univariate and multivariate models
Supplemental Table 4: CATHGEN sensitivity analyses including hyperlipidemia and hemoglobin in multivariate model
Supplemental Table 5: GSEA Pathway Analysis
Supplemental Table 6: Protein associations for HFrEF versus non-HF in TECOS
Supplemental Table 7a: Area under the curve (AUC) for LASSO protein model and validating proteins for incremental discriminative capabilities
Supplemental Table 7b: Area under the curve (AUC) for LASSO protein model and validating proteins for discrimination of HFpEF cases vs. non-HF controls in sex-stratified analyses
Supplemental Table 8: Baseline characteristics of Jackson Heart Study participants included in all-cause mortality and incident HFpEF hospitalization analysis
Supplemental Table 9: HFpEF proteins associated with incident hospitalization for heart failure
Supplemental Table 10: HFpEF proteins associated with incident HFpEF hospitalization stratified by baseline HF status
Supplemental Table 11: HFpEF proteins associated with incident HFpEF hospitalization in JHS
Supplemental Table 12: Meta-analysis of HFpEF proteins associated with all-cause mortality
Supplemental Table 13: HFpEF proteins associated with all-cause mortality stratified by baseline HF status
Supplemental Table 14: Significant HFpEF-associated proteins across all analyses
Supplemental Table 15: Spearman correlation of significant proteins in 369 JHS participants on Olink 1200 and 1500 platforms
